# Alkyne-tagged imidazolium-based membrane cholesterol analogs for Raman imaging applications†

**DOI:** 10.1039/d4sc03155e

**Published:** 2024-08-07

**Authors:** Constanze Schultz, Tristan Wegner, Corinna Heusel, Tim Gallagher, Yanjun Zheng, Markus Werner, Seraphine V. Wegner, Tobias Meyer-Zedler, Oliver Werz, Michael Schmitt, Juergen Popp, Frank Glorius

**Affiliations:** a Leibniz Institute of Photonic Technology (Leibniz-IPHT), Member of Leibniz Health Technologies, Member of the Leibniz Center for Photonics in Infection Research (LPI) Albert-Einstein-Str. 9 07745 Jena Germany juergen.popp@leibniz-ipht.de; b University of Münster, Institute of Organic Chemistry Corrensstraße 40 48149 Münster Germany glorius@uni-muenster.de; c University of Münster, Institute of Physiological Chemistry and Pathobiochemistry Waldeyerstraße 15 48149 Münster Germany; d Department of Pharmaceutical/Medicinal Chemistry, Institute of Pharmacy, Friedrich-Schiller-University Jena Philosophenweg 14 07743 Jena Germany; e Institute of Physical Chemistry (IPC) and Abbe Center of Photonics (ACP), Member of the Leibniz Center for Photonics in Infection Research (LPI), Friedrich Schiller University Jena Helmholtzweg 4 07743 Jena Germany

## Abstract

Cholesterol is an important lipid playing a crucial role in mediating essential cellular processes as well as maintaining the basic structural integrity of biological membranes. Given its vast biological importance, there is an unabated need for sophisticated strategies to investigate cholesterol-mediated biological processes. Raman-tagged sterol analogs offer the advantage of being visualizable without the need for a bulky dye that potentially affects natural membrane integration and cellular interactions as it is the case for many conventionally used fluorescent analogs. Herein, we report a series of alkyne-tagged imidazolium-based cholesterol analogs (CHIMs) with large Raman scattering cross-sections that readily integrate into HEK cells and primary monocyte-derived macrophages and allow (multiplexed) cellular Raman imaging. We envision Raman-tagged CHIM analogs to be a powerful platform for the investigation of cholesterol-mediated cellular processes complementary to other established methods, such as the use of fluorescent analogs.

## Abstract 2

Headgroup-labelled alkyne-tagged cholesterol mimetics with imidazolium cores were designed and probed for their use as Raman probes in mammalian cells. The analogs feature high membrane integration and tunability of their vibrational signature.

## Graphical abstract

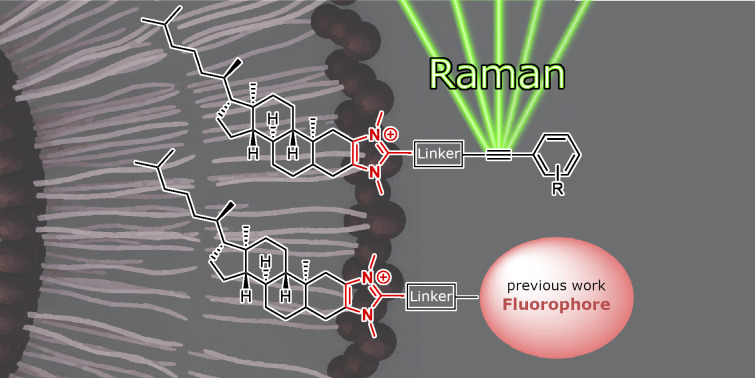

## Introduction

The sterol lipid cholesterol is an essential ingredient of mammalian cell membranes. It plays a vital role in regulating the cell membrane's structural integrity and fluidity as well as in mediating essential cellular processes such as signal transduction and membrane trafficking.^1,2^ Correspondingly, dysfunction of natural cholesterol homeostasis has been proposed to have extensive cellular implications and has been linked to the emergence of diseases such as Alzheimer's or cancer.^3–8^ To investigate and better understand cholesterol-mediated biological processes, the development of new and improved tools is of high relevance. A widely established, easily applicable, and very sensitive method for studying the spatial and temporal distribution of biomolecules in their natural environment is fluorescence microscopy. However, most lipids are intrinsically nonfluorescent, thus making it necessary to implement appropriate external labels for visualization purposes as it has been done for fluorescent cholesterol analogs such as BODIPY- or NBD-cholesterol.^9–13^ While such cholesterol mimetics have extensively been applied for the investigation of cholesterol-mediated processes *via* fluorescence microscopy, the implementation of a fluorescent moiety often affects the amphiphilic structure and natural membrane integration of the analogs, thus severely limiting their applicability as valid probes for the investigation of cholesterol-related cellular processes.^14–20^

**Fig. 1:**
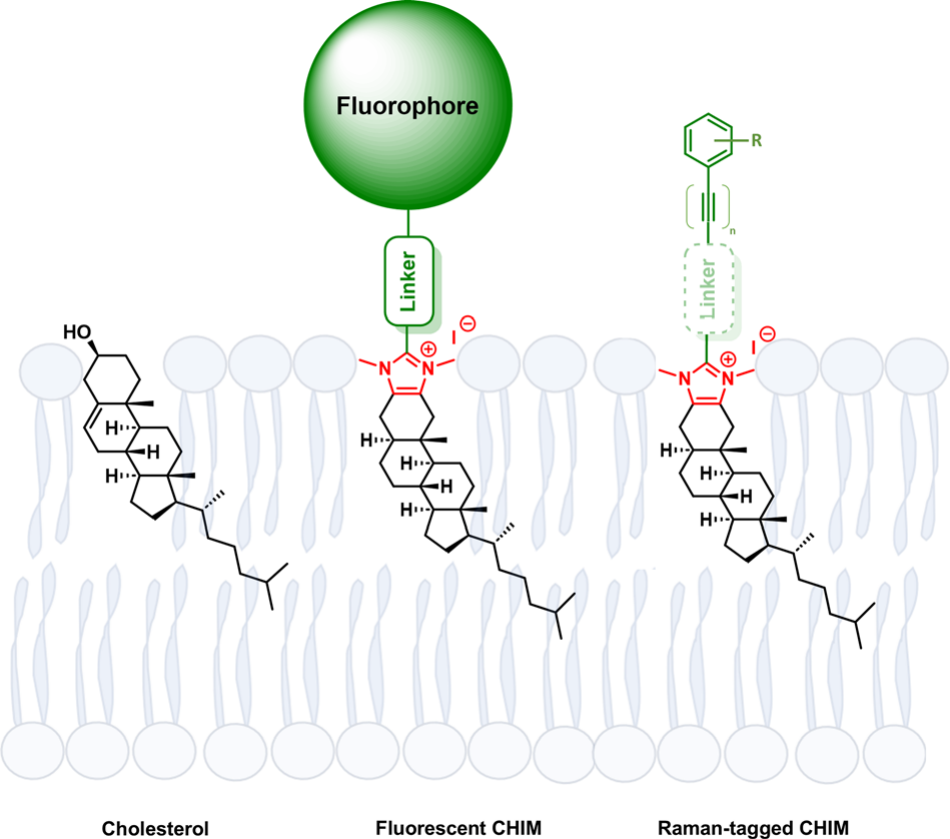
Molecular structure and schematic depiction of membrane integration of natural cholesterol, fluorescently labeled CHIM^28–31^ as well as of an herein investigated Raman-tagged CHIM derivative (red: imidazolium headgroup, green: labelling moiety).

We recently introduced a novel class of imidazolium-based lipid analogs that exhibit an amphiphilic structure similar to natural lipids and allow flexible molecular tuning of their function and biological properties.^21–27^ In particular, a series of cholesterol analogs (cholesterol-based imidazolium salts, CHIMs) was developed that have been shown to exhibit cholesterol-like properties, *e.g.* by readily integrating into biological membranes, and could be applied for fluorescence imaging of cholesterol dynamics and distribution in live cells.^28,29^ In contrast to many previously established fluorescent or clickable cholesterol mimetics the reported CHIM analogs were designed in a way that leads to a positioning of the attached fluorophore outside of the membrane while maintaining an overall amphiphilic molecular structure similar to natural cholesterol. In doing so, we envision the effect of the analogs' structural modifications on their proper membrane integration to be minimized (Fig. 1). However, even in this case, the attachment of a bulky fluorophore constitutes a significant structural change compared to the natural lipid and thus can potentially affect the analogs' faithful biological behavior.

A gentler approach to yield orthogonality to the enclosing matrix is the introduction of vibrational tags with frequencies in the wavenumber silent region for Raman-based applications. The relative merits of vibrational *versus* fluorescence tags have been extensively discussed over the past decade^32–37^ and nowadays find a multitude of specialized applications in bioimaging.^34,38–41^ Among the available tag options, alkynes are usually favored owing to their comparably high Raman intensity and simple band shape.^42–47^ However, exploiting wavenumber silent region tagging strategies for design of cholesterol mimetics, and particularly with alkyne tags, are rare so far.^37,48,49^

Previously, a Raman-tagged cholesterol analog has been reported in which a phenyl-capped diyne was implemented into the lipophilic tail of a cholesterol scaffold.^49^ While such an analog was shown to be readily internalized into cells and thus could be applied for assessing intracellular cholesterol storage, plasma membrane staining was less pronounced (in particular for longer incubation times).^49^ In that regard, the implementation of the Raman tag into the lipid backbone will necessarily position it deep inside the membrane, which might impede faithful membrane integration of the analog. Thus, we anticipate the incorporation of the tag as a part of the head group as more beneficial for the development of membrane cholesterol mimetics. A first approach to head-labelled alkyne cholesterol derivatives was introduced by Yamaguchi *et al.*^37^ A terminal alkyne moiety was created *via* Eschenmoser fragmentation under destruction of sterane ring A. The signal of the triple bond was then detected within granular structures and within the cytosol by Raman spectroscopic techniques under mildly acidic conditions that were required for alkyne formation.^37^ Despite the aim of positioning the tag close to the membrane surface to provide an easily accessible reaction point, evidence of probe accumulation in plasma membranes was lacking here. The implementation of more efficient (*e.g.* end-capped) tags was not shown but could be disadvantageous due to steric requirements within the precursor.

Against this backdrop, we rationalized that a modular approach to headgroup labelled cholesterol analogs under retention of the sterane core structure could empower the design of membrane cholesterol mimetics while simultaneously allowing careful tuning between the tag's scattering efficacy and cellular localization.

In this work, we thus sought to extend the CHIM family by modifying our previously reported CHIM analog in a way that allows its visualization *via* Raman imaging. By introducing the Raman tag as a part of the analog's imidazolium headgroup (Fig. 1), the lipophilic backbone was left mostly untouched to ensure a membrane integration behavior as faithful as possible. Additionally, along with the replacement of the polar head group to annulated imidazolium derivatives, downstream metabolic pathways of the natural cholesterol, such as droplet storage initiated by esterification of the cholesterol hydroxy group inside cells^50^ can be expected to be hindered. It is thus that our CHIM analogs can be envisioned to be a particularly valuable tool for tracking cholesterol in the context of membrane processes.

**Fig. 2:**
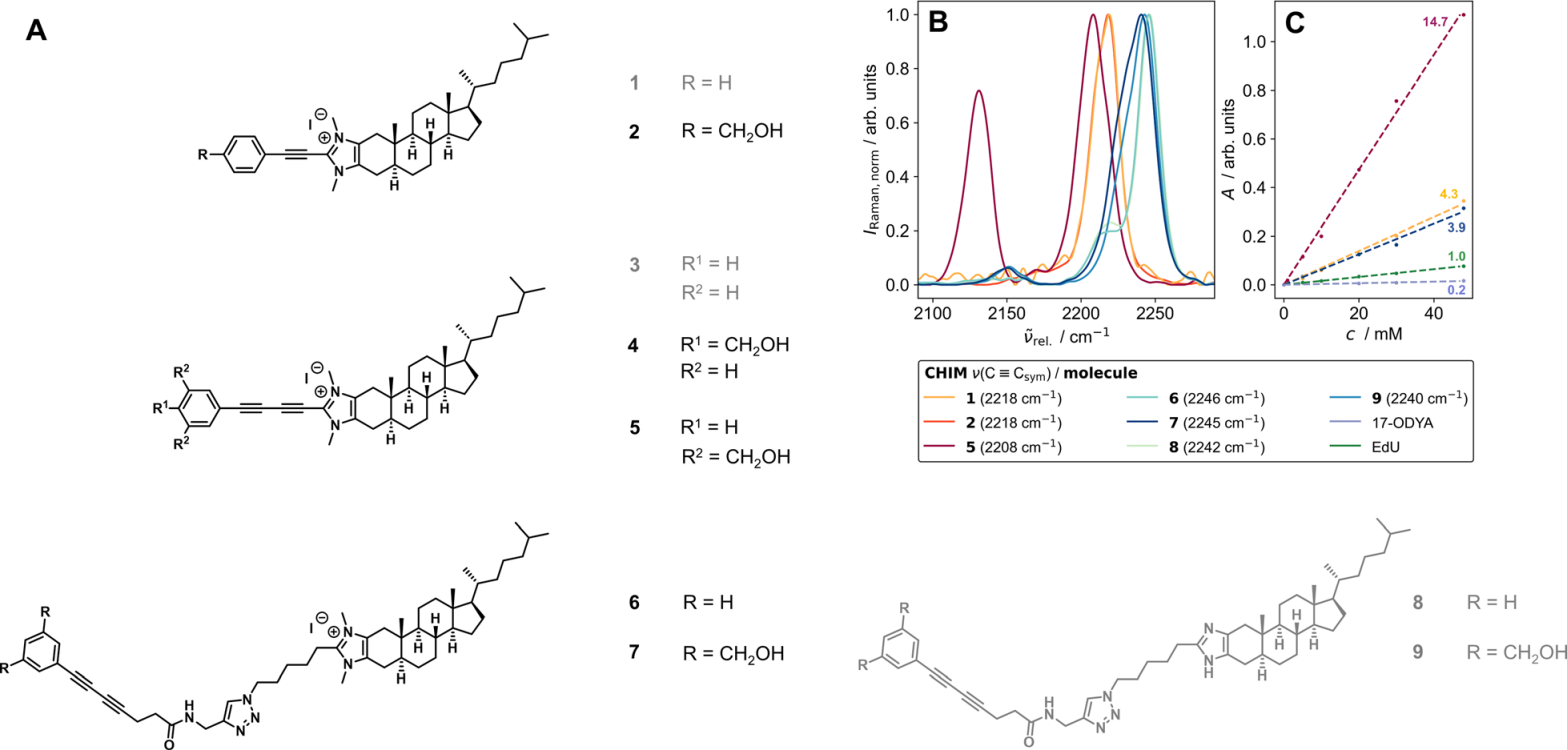
(A) Herein synthesized and investigated Raman-tagged CHIM analogs. The optimized substances of each design step that were later successfully applied in cell experiments are highlighted in black. Gray compounds could not be incorporated into cells. (B) Band positions of the triple bond stretching vibration of the alkynes (1 and 2), a diyne with both-sided conjugation extension (5), and diynes with a broken conjugated system (6–9) obtained from FT-Raman measurements of the solids at 1064 nm excitation. With increasing conjugation length, the band of the (symmetric) alkyne stretching vibration shifts to shorter relative wavenumbers. The spectra were corrected for water absorption bands and normalized to the highest band in the range 2100–2300 cm^−1^. (C) Comparison of the area under the triple bond's symmetric stretching band as a function of molecule concentration in DMSO for CHIMs and commercially available alkyne-tagged small molecules (17-ODYA and EdU) with RIE values.^42^ All synthesized CHIMs exhibit significantly higher molar Raman intensities (respectively scattering cross-sections) than both commercial references.

We synthesized a variety of different Raman-tagged CHIM analogs (Fig. 2A) and evaluated their Raman properties and applicability for the tracking of cholesterol distributions in cellular membranes. We show that five of our designed analogs are indeed readily integrating into the plasma membranes of HEK 293T cells and that their spatial distribution in the cell can be visualized by spontaneous Raman microspectroscopy. Additionally, we demonstrate the multiplexing capability of the designed CHIM analogs, both, in combination with themselves as well as with 5′-ethinyl-2′-deoxyuridine (EdU) accumulating in the cell nucleus. Overall, the presented investigations showcase the potential of the herein-reported class of alkyne-tagged CHIMs as potent Raman probes and useful tools for studying membrane-cholesterol-mediated cellular processes.

## Results and discussion

### Molecular design & tag properties

One crucial parameter in developing potent Raman tags is the size of their conjugated π-electron system. Both, increased polyyne chain length as well as the extent of the enclosing system of delocalized electrons significantly enhance the Raman intensity of the tag which especially favors aromatically capped polyynes.^42,44–47,49,51^ For this reason, a first set of compounds (1–5) was designed in which the cholesterol analogs were equipped with a phenyl-capped Raman tag containing either one or two triple bonds directly attached to the C2 position of the imidazolium ring. We envisioned this synthetic design to offer a sufficiently extended π-system size of the tag yielding high Raman intensities as well as to allow for a modular synthesis of different alkyne-tagged CHIM derivatives using Cu-mediated C–H-activation^52^ (Schemes S1–S5†) and a low perturbing orientation of the tag outside of the membrane as previously reported for other C2-modified CHIM analogs.^28–31^ However, it is important to note that by extensive elongation of both, the polyyne chain length and/or the surrounding π-electron system (as it has *e.g.* been reported for recently developed high-intensity polyyne probe palettes^51,53^) a growing, extremely rigid element is introduced that eventually might also affect properties and behavior of the tagged biomolecule. In that regard, we observed solubility issues even with rather small alkyne elements (1) in water as well as with diyne elements (3) in DMSO, potentially due to effective π-stacking of the elongated, rigid π-systems.^44,47^ We rationalized that addition of polar hydroxymethyl substituents (2, 4, 5, 7 and 9) could compensate for the observed poor solubility of fully conjugated diynes without largely affecting the Raman frequency (Fig. 2B). Additional details on the structural tuning of the vibrational frequency can be found in the ESI (Fig. S1).† Furthermore, it was hypothesized that a less rigid and more flexible design in which the Raman tag is attached to the CHIM analog *via* a clickable azide linker (6–9) would be less prone to π-stacking and thus could avoid particle formation and poor solubility of the compounds in aqueous medium. In that case, the diyne element, despite its broken π-conjugation, was still found to provide sufficient molar Raman intensity that can compete with the one of an alkyne in full conjugation but shows a significantly lower intensity in comparison to a fully conjugated diyne (Fig. 2C). Notably, all tested CHIMs showed significantly higher molar Raman intensities than frequently used and commercially available triple bond tagged biomolecules (Fig. 2C, for details on RIE ratios see ESI Table S1, Fig. S2 and 3†).

### Application of CHIMs for cell imaging

Next, we sought to investigate whether the herein synthesized and structurally optimized Raman-active CHIM analogs would indeed exhibit cholesterol-like properties, *e.g.* by readily integrating into cellular membranes, and thus could potentially serve as useful tools for the tracking of cholesterol-mediated cellular processes as it was previously shown for the respective fluorescent CHIM analogs.^28–31^ In analogy to our previous studies,^28–31^ we conducted a short-term treatment on ice for 20 min. The concentration within the treatment solution was 100 μM which aligns with the earlier studies on vibrationally tagged cholesterol mimetics in long term incubation.^38,48,49^

**Fig. 3:**
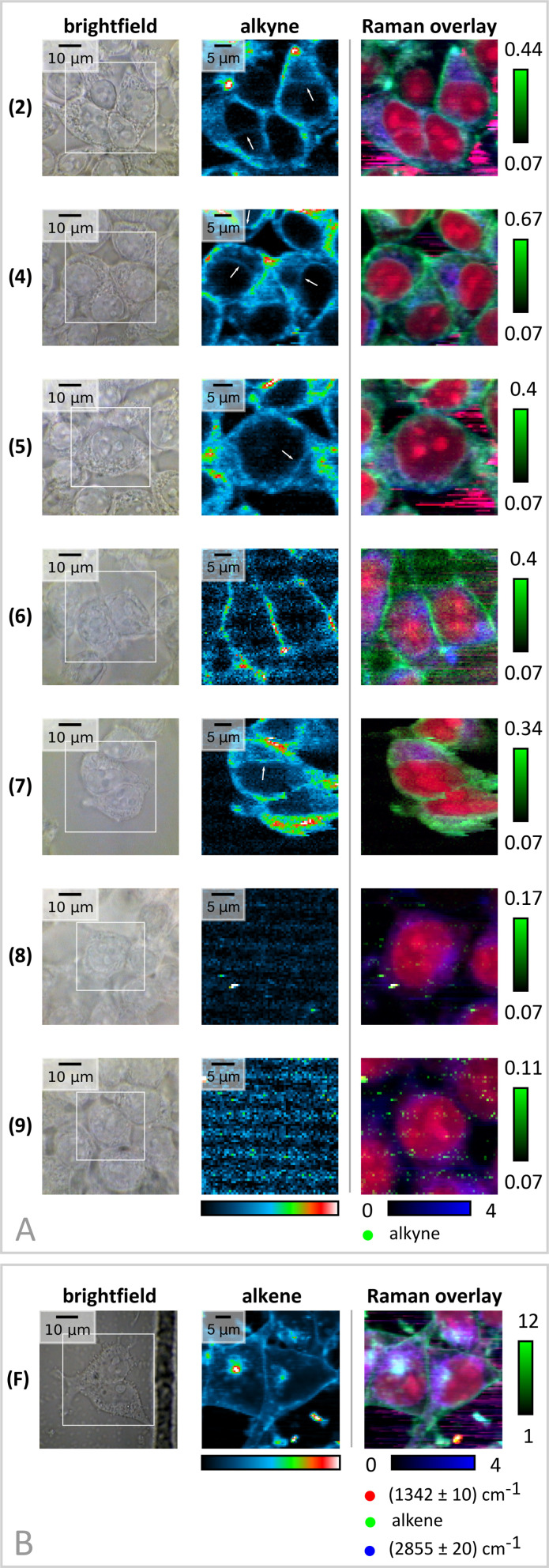
(A) Spatial cellular distribution of tagged CHIMs after treatment of starved HEK 293T cells with a 100 μM CHIM solution for 20 min on ice. The analogs 2, 4, 5, 6 and 7 are readily integrated into cells. While CHIM 6 seems to only reside in the cell membrane analogs 2, 4, 5 and 7 are spread in the cytoplasm or show prominent labelling of the nucleus' plasma membrane as well (white arrows). No cellular uptake was visible for the imidazole-based analogs 8 and 9. Panel (B) displays the distribution of filipin III (F), identified by its C=C double bond. The figure shows the brightfield camera image (left, scale bar: 10 μm, measured area indicated by the white square), the distribution image of the alkene/alkyne (middle, colors scaled individually within the measured area) and an RGB overlay image (right, red: nucleus, blue: CH_2_, green: alkyne/alkene). For color scaling of the RGB image please refer to the Experimental section.

By tracking the band associated with the C≡C stretching vibration, it was shown that analogs 2, 4, 5, 6, and 7 are incorporated into HEK cells under our short incubation conditions (Fig. 3A). Here, the integration maps of the expected alkyne spectral position (Fig. 3A, middle in each panel) visualize the distribution within the measured area. The RGB images (Fig. 3A, right in each panel) allow precise localization of the CHIM analogs in the cell based on color-coding of cell areas that can be uniquely identified by characteristic vibrational bands. The nucleus was segmented by integrating the band at 1342 cm^−1^, which is a marker for proteins and purine base nucleosides,^54,55^ while the symmetric CH_2_-stretching vibration at 2855 cm^−1^ was chosen for the identification of lipid-rich biomass outside the nucleus.^56^ All five incorporated analogs seem to be located mainly in the cell plasma membranes as well as to some extent inside the cell (Fig. 3, S7 and S14†).

This observation is in accordance with former investigations of fluorophore-conjugated CHIM derivatives that also showed a pronounced plasma membrane localization.^28–31^

Additionally, we compared the localization of our designed CHIM derivatives inside the cells with results obtained from a commercially available isotopically labeled cholesterol probe (Fig. S5 and 6†) and results from counterstaining experiments with the gold standard filipin III (Fig. 3B and S17–20†). Filipin III is a polyene macrolide and known to stain unesterified cholesterol in fixed cells, although the detailed mechanism of action is not yet fully understood.^57^ We analyzed the distribution of filipin III inside cells in detail with two modalities, Raman and fluorescence imaging (compare ESI Fig. S17–20†). Filipin III is typically excited in the UV A range giving rise to fluorescence between 400 nm to 600 nm.^29,57–59^ HEK cells were stained with filipin III as described in the Experimental section and analyzed by two photon excited fluorescence (TPEF) with excitation at 700 nm. A spectrally resolved scan of the recorded TPEF signal is displayed in Fig. S17.† The observed emission spectrum matches well with spectra reported elsewhere.^60,61^ Fluorescence imaging as the conventional way of filipin III localization enabled us to get a first visual impression of the distribution of cholesterol in the same area of the cell dish, where we acquired Raman images before filipin staining. Fluorescence imaging of filipin III confirmed the predominant localization of native cholesterol in cellular membranes of untreated and CHIM-treated HEK cells. A detailed study of filipin distribution in 2D and 3D space can be found in Fig. S18–S20,† where we also provide a comparison in position with our designed CHIMs.

Tracking of the polyene macrolide antibiotics filipin by Raman spectroscopy is possible as well when considering the band assigned to C=C double bonds, whose area under the curve significantly supersedes native levels in spots of filipin III accumulation (Fig. 3B and S20†). Hyperspectral Raman imaging eventually also facilitated a pixel-by-pixel colocalization analysis, which generally revealed a high degree of colocalization between filipin III and CHIM accumulation spots (Fig. S20†), allowing us to conclude that the CHIM analogs presented here reflect the behavior of natural cholesterol in mammalian cells that, being an essential membrane ingredient, has been found to mainly reside in the plasma membrane. Highly comparable results were also obtained for a deuterated cholesterol version (*cf.* cholesterol-d_6_ in Fig. S5 and 6†), although its scattering efficiency is much worse compared to our designed CHIMs.

Moreover, by referencing all individual RGB channels in Fig. 3 to the mean signal in the nuclei regions (excluding nucleoli) direct comparisons between different CHIMs become possible (Fig. 3A and S7†). First, the lipid-to-protein ratio in nuclei and cytoplasm aligned very well for the examples depicted in Fig. 3A, which is a first indicator on cell wellbeing (Fig. S7A–C†). Further proof is given by an MTT test for two differently designed CHIMs that overall show no substantial toxicity compared to the control cells subjected to the same treatment but without CHIM addition (Fig. S9†).

Second, within this first proof of concept (Fig. 3), CHIM 4 showed the highest and most uniform signal efficiency. The limit of detection (LOD) was estimated for this component within the cells with 20 μM ≤ LOD < 50 μM (Fig. S8†) which is well in agreement with the earlier published and vibrationally-tagged cholesterol mimetics.^37,48,49^ Third, most of the herein designed and incorporated CHIMs still seem to retain some mobility inside the cell as also labelling of the membrane surrounding the nucleus was observed (Fig. 3A, white arrows). As shown for CHIM 4, continued incubation after removal of the CHIM supply also resulted in accumulation in the inner cell structures (Fig. S13†), as observed for the previously published head-labelled alkyne cholesterol mimetic.^37^ Nevertheless, CHIM 4 still retained visible membrane labeling activity in those cases. CHIM 6, instead, is specifically confined to the cell plasma membrane area (Fig. 3A and S7E†) even for longer incubation times (Fig. S12†). Comparing the *N*-methylated imidazolium salts 6 and 7 with their non-ionic imidazole equivalents 8 and 9, no cellular uptake could be observed for these non-permanently charged compounds (Fig. 3). Instead, particle formation was observed for 8 and 9, indicating a lower solubility in the aqueous solution that might inhibit cellular uptake. In that regard, a well-established approach to shuttle hydrophobic, water-insoluble lipids into cells is their complexation with bovine serum albumin (BSA)^62,63^ or, particularly in the case of cholesterol, the complexation with methyl-β-cyclodextrin (MβCD).^64,65^ Both approaches however did not succeed for the imidazole analogs (Fig. S10 and S11†) which classifies the permanently charged imidazolium moiety as indispensable for cellular imaging applications.

At the same time, however, the absence of signals in the intensity images evaluating the expected peak range of CHIMs 8 and 9 and the presence of signals for the other 5 CHIMs (Fig. 3), confirm the biological and spectroscopic orthogonality of the designed tags.

Since there are no naturally occurring bands in the silent wavenumber region present in HEK 293T cells, our designed tags have a high specificity which validates the successful design of 5 analogs mimicking cholesterol properties. Due to the high signal strength allowing for fast and unambiguous detection, we outperform commercially available alternatives such as the deuterium-tagged analog (Fig. S5 and S6†).

### The potential of Raman-tagged CHIMs in multiplexing experiments

Besides their small size allowing for a more faithful biological behavior of the tagged compound, another benefit of vibrational tags in comparison to fluorescent labels is the significantly lower spectral width. In particular, the slowly dipping tails in the absorption and fluorescence spectra of fluorescent dyes demand a careful choice of markers to avoid crosstalk and bleed-through in multiplexing experiments. The application of several fluorescent markers thus usually poses the necessity to spread the detection windows to the whole visible range and limits the number of detectable dyes to a maximum of 3–4 in simultaneous imaging if no more elaborated imaging techniques such as FLIM,^66^ selectivity controlling excitation schemes,^67^ spectrally resolving detection^68^ and acquisition methods followed by image reconstruction^69^ or emitters with extremely narrow tunable spectra (*e.g.* quantum dots)^70,71^ are used.

**Fig. 4:**
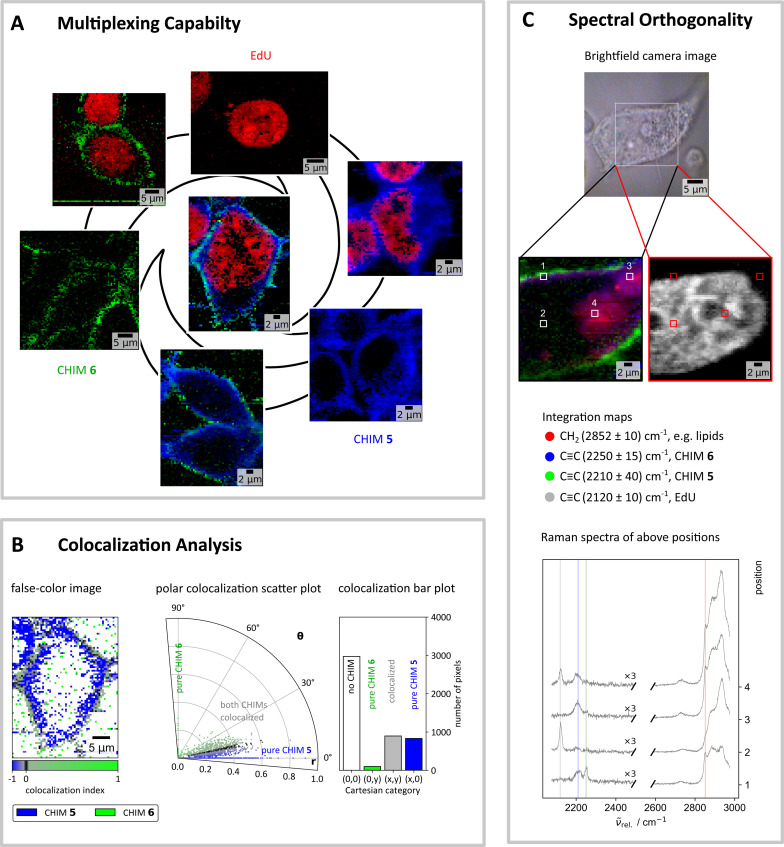
Spatial distribution of the band intensities of the up to three labelled compounds, simultaneously applied in HEK 293T cells. (A) The distribution of the tagged components was retrieved as the area under the peak by a fitting approach (see ESI†). The outer circle shows the results for single-component and double-component incubations (CHIM 6: 100 μM, CHIM 5: 100 μM, CHIM 5/CHIM 6: 50 μM/100 μM, EdU: 1 mM, EdU/CHIM 6: 1 mM/100 μM, EdU/CHIM 5: 1 mM/100 μM). The image in the middle was obtained after incubation of HEK cells with all three tagged compounds (EdU/CHIM 5/CHIM 6: 1 mM/50 μM/100 μM). The colors in the images refer to the specific components: red: EdU, blue: CHIM 5, and green: CHIM 6. (B) The colocalization analysis identifies both CHIMs to be partly colocalized (polar plot, middle, and bar plot, right). The areas of colocalization are spatially located at the cell plasma membrane's site. CHIM 5 shows higher mobility inside the cell than CHIM 6 as visible by the differing penetration depths within a focal area in the *x*–*y*-plane (left, false color colocalization image). CHIM 5 is shown in blue, CHIM 6 is shown in green. Areas of colocalization of both CHIMs are shown in black or grayish color, depending on the degree of colocalization. Pixels were none of both CHIMs was present are visualized by white color. (C) Spectra and distribution maps of a cell treated with three triple bond-tagged molecules (CHIM 5, CHIM 6, EdU) imaged with a better spatial (3 px per μm) and spectral (grating: 1800 g mm^−1^) resolution as compared to (A). Representative average spectra of the indicated positions depict the different relative distribution of the three tagged molecules in various cell regions. The selected positions are indicated in the distribution maps. The distribution maps (middle) were obtained after integration around the indicated center positions in the spectra (bottom; gray: EdU, blue: CHIM 5, green: CHIM 6, red: CH_2_).

In comparison, the small spectral width of alkyne vibrational bands strongly facilitates and increases their applicability for multiplexing. The basic concept of simultaneous detection of various alkyne tags was already demonstrated for up to 15 triple bond-tagged molecules on polystyrene beads and up to five triple bond-containing molecules for direct incubation in cells.^51^ Due to the well-defined band shapes in linear Raman spectroscopy, resolving partially overlapping bands is possible and straightforward. To demonstrate the aforementioned advantages of vibrational tags and to show the compatibility of our herein-designed CHIMs in combination with other vibrational markers a series of multiplexing experiments was performed (Fig. 4).

Overall, the successful detection of up to three alkyne-tagged molecules including the two diyne-tagged CHIMs 5 and 6 and the alkyne-modified nucleoside EdU by linear Raman microscopy in one cell with no significant cytotoxicity was demonstrated. In addition, we investigated all possible combinations of these three substances in simultaneous application to exclude potential changes in their distribution due to possible mutual influences that might occur in interbatch comparisons as shown in Fig. 3.

For multiplexing experiments, we chose CHIM 5 and 6 since, in addition to their different design approaches, they emerged as technically best suited for cell experiments.

For analyzing the spatial distribution of the applied probes, all bands arising in the wavenumber silent region were fitted by Voigt profiles and the corresponding area under the peak was extracted as the amplitude parameter (for details of band fitting procedure see ESI Fig. S15†). The obtained results demonstrate the validity of the fitting approach and confirm a spacing of 30 cm^−1^ between band centers to be sufficient to separate two different components, even when featuring different scattering cross-sections.

Fig. 4A shows the spatial distribution of the three tagged molecules (color-coded by substance and projected on an 8-bit scale by band area).

Demonstrating biorthogonality, no significant changes in the spatial distribution between the single tag and multiple tag experiments became apparent. EdU and any of the used CHIMs behave spatially orthogonal and can thus be applied for labelling different cell constituents when elucidating biological processes. Their bands are furthermore spectrally fully resolved (Fig. 4C). In comparison to already reported alkyne-labeled steroid analogs^37,49^ the herein investigated analogs showed a particularly pronounced localization at the cell membrane (as opposed to extensive internalization) thus complementing the palette of so far available sterol-derived labelling tools.

Although both investigated CHIMs label roughly the same region in the cell, their structural differences appear to induce slight variances in their distribution. The colocalization analyses (Fig. 4B and continued in more detail in Fig. S16†) confirm the different penetration depths of the two CHIMs that were already observable to a certain extent in the single CHIM incubation experiments (Fig. 3). The here-conducted simultaneous application also excludes artifacts potentially caused by the uncertainty factor in cell concentration between different dishes and therefore allows for more precise spatial comparison. Unlike its counterpart 6, CHIM 5 shows a spatially wider distribution also revealing internalization into the cytoplasm as inferred from the distribution image (Fig. 4B, left) and the bar plot (Fig. 4B, right). The colocalization zone of both CHIMs (Fig. 4B, grayish and black) is mainly confined to regions around the plasma membrane with the ratio of CHIM 5 : CHIM 6 increasing from the outside to the inside of the cell within a focal plane. Restricted or slowed-down mobility inside the cell could possibly be ascribed to the degree of modifications to hydrophilicity. Tag size may generally also be a crucial factor, however in this case the equally sized CHIM 7 showed no severely constrained distribution (Fig. 3). Overall, the obtained results showcase that the careful structural design of alkyne-tagged CHIMs seems to not only allow tuning of their Raman properties as shown before but potentially also of their precise intracellular behavior, thus eventually offering a toolbox of analogs for different labelling needs.

### Transferability of the designed CHIMs

Although HEK 293 cells are a widely established immortalized human cell line for studying cholesterol uptake, depletion and homeostasis and the effect of cholesterol levels on cellular functions such as protein synthesis, receptor functions and membrane mechanics,^72–77^ a transferability of results to native characteristics is limited. We thus decided to run another set of experiments, this time on human primary cells. Primary cells are directly derived from living human tissue or blood and thus more realistically mimic *in vivo* settings, including native cell heterogeneity, as it might be desired for future applications. Consequently, we repeated the short-term uptake experiment with CHIM 4 under more physiological conditions with macrophages derived within 6 days from primary blood monocytes of two different human donors.

**Fig. 5:**
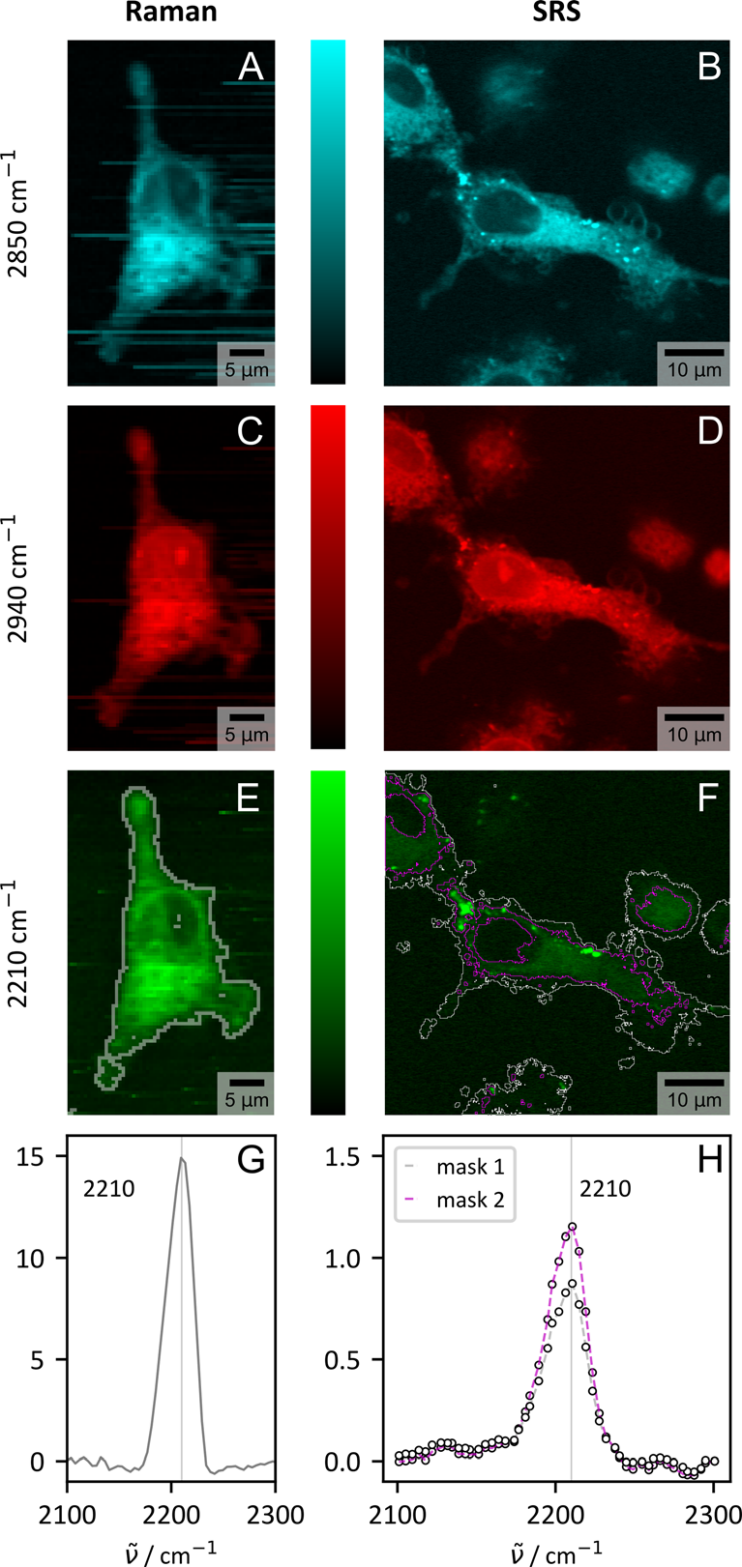
Visualization of CHIM 4 in primary human macrophages from one representative donor by spontaneous (Raman) and stimulated Raman scattering (SRS). Shown are the results for three selected wavenumbers which visualize the distribution of lipids (–CH_2_, 2850 cm^−1^, cyan, (A) and (B)) proteins (–CH_3_, 2940 cm^−1^, red, (C) and (D)) and the tagged CHIM (–C≡C–, 2210 cm^−1^, green, (E) and (F)). For spontaneous Raman scattering the images were generated from integrating the area under the spectrum in a range of ±20 cm^−1^. The tagged cholesterol analogue is clearly visible within the cell by both modalities. (G) and (H) show the spectral profile in the silent wavenumber region within the cell area (*ImageJ* thresholding, Huang:^78^ gray, Moments:^79^ purple, see (E) and (F) for mask borders). The spectra were normalized to the signal count in the non-peak area (spontaneous Raman: average signal 1800–2100 cm^−1^, SRS: signal at 2130 cm^−1^).

Also here, CHIM 4 was clearly identifiable within the cell for both donors by its C≡C stretching vibration using spontaneous Raman microspectroscopy (Fig. 5 and additional examples in ESI Fig. S21†). In the reference dishes without CHIM 4 addition, no remarkable signal within the silent wavenumber region appeared (Fig. S22†). The presented results (Fig. 5, S21 and S22†) thus confirm the biorthogonal targeted incorporation and detection of the vibrationally-tagged cholesterol analog, thereby providing evidence for the results' generalizability and transferability across different cell types.

Within a biomedical context, another aspect also emerges which is closely linked to the transferability of the CHIMs. While spontaneous Raman scattering is fully sufficient for stationary tasks, including the overall CHIM distribution within cells as evaluated in Fig. 3 and 4, imaging in a more complex context might necessitate faster imaging speeds for resolving time critical questions. Stimulated Raman scattering (SRS) can fill this gap.^80,81^Fig. 5 compares the results of spontaneous and stimulated Raman scattering for three selected Raman modes and confirms the fast detection of CHIM 4 by SRS within the cell when in resonance with the expected Raman mode (Fig. 5G–H and ESI S23†). Here we employed the significantly faster imaging speed that is required for biomedical issues (1–2 s per px for Raman and 120 μs per px including averages for SRS) for higher resolution images and expansions of the imaged area in *xy*- (Fig. S26†) and *z*-direction (Fig. S24†).

The ability to measure *z*-stacks with a digital spatial resolution of ≈0.2 μm in all directions is equivalent to what we outlined for filipin III detection *via* TPEF (Fig. S18†) and allows for a reconstruction of the cell based on the cholesterol signal (Fig. S24†). Here, SRS is superior in terms of practical application to spontaneous Raman scattering, where *z*-information could also be retrieved, but for time reasons only from a narrow cuboid (Fig. S25†). Both imaging modalities clearly confirm the uptake of the provided tagged CHIM as a full staining of the cells, but not the nucleus, was observed (Fig. S24 and 25†). The result aligns with earlier observations on HEK cells with CHIM 4 having the most uniform distribution but very intense uptake (Fig. 3 and S7†). Large area images (Fig. S26†) deliver insights in the uptake statistics in cells with native heterogeneity. We do observe some variations in signal intensity within the primary macrophages. However, no cell showed no uptake which makes the CHIMs a very reliable labelled cholesterol source.

## Conclusions

A series of alkyne-tagged imidazolium-based cholesterol analogs (CHIMs) was designed, synthesized, and investigated for their Raman properties as well as for their applicability for imaging of cellular cholesterol distributions in HEK cells and human primary macrophages. We demonstrated that five of our designed analogs are indeed readily integrating into the plasma membranes of HEK cells and that their spatial distribution in the cell can be visualized by linear Raman microscopy. Here, high molar Raman intensities combined with pronounced accumulation in cell plasma membranes enabled a facile identification of the tag. The used CHIM concentrations, less or equal to 100 μM in the cell treatment solution, that match the concentration range reported in the literature for nonlinear imaging of several other alkyne-tagged small molecules^43,82^ as well as the large scattering cross-sections compared to EdU and 17-ODYA demonstrate the competitiveness of the designed tags with commercially available molecules. In addition, the multiplexing capabilities of the designed CHIM analogs were showcased. It was shown that small structural modifications of the analogs can greatly affect their Raman properties as well as membrane integration and intracellular localization, thus potentially offering a toolbox for the tailor-made design of Raman-active cholesterol analogs depending on the respective labelling/imaging requirements. The observed impact of only subtle structural modifications on precise intracellular positioning underscores the imperative for considering vibrationally tagged cholesterol derivatives as a promising alternative to more extensively structurally perturbed fluorescently tagged cholesterols. For future applications, we regard tracking of the uptake or of distribution dynamics with fast image acquisition speed using our innovative cholesterol probes in combination with nonlinear vibrational imaging techniques, such as SRS or (broadband) CARS (Fig. S4†), as conceivable. An initial proof of concept concerning the transferability to coherent nonlinear techniques has already been successfully applied in CHIM-tagged cells for depth and large area screens. The experiments thereby also underline the competitiveness of in particular stimulated Raman scattering in combination with vibrationally-tagged CHIMs to the conventional fluorescence gold standard in terms of image acquisition performance.

Overall, the presented investigations demonstrate the potential of the herein reported class of alkyne-tagged CHIMs as potent and tunable cellular Raman probes that we envision to constitute a versatile platform for the investigation of cholesterol-mediated membrane processes in a way complementary to other established methods, such as the use of fluorescent analogs.

## Experimental

### FT-Raman measurements of solids and solutions

The Raman spectra of the CHIMs as a solid and in DMSO solutions were acquired with a Bruker MultiSpec Fourier transform (FT)-Raman spectrometer equipped with an external liquid nitrogen Ge-diode detector and a diode-pumped Nd:YAG laser (Klastech DeniCAFC-LC-3/40, Germany), emitting laser light at *λ* = 1064 nm as the excitation source. The excitation power was set to 1000 mW (500 mW to prevent sample burning in the case of the darker solid samples 1, 2 and 5). For Raman measurements of the solids, the laser beam was focused on a small amount of pressed crystal powder, while the spectra of the CHIM-DMSO solutions were obtained from measurements in a quartz glass cuvette backed by a mirror to increase the intensity of the epi-detected signal by reflection. The spectra were recorded with a spectral resolution of 4 cm^−1^ in the range from 0–4000 cm^−1^ with OPUS 6.5 (further settings: apodization: Blackman-Harris 4-term, zero-filling factor: 16, phase correction: power, no peak search, resolution: 4, aperture: 5 mm, mirror speed: 5 kHz). Post-processing was done in Python applying a removal algorithm for strong water absorption bands and a SNIP-background correction algorithm.^83,84^

### Hyperspectral Raman imaging of cells

A confocal Raman microscope (alpha300, WITec, Germany) with a 60×/1.0 NA water-dipping objective (Nikon NIR Apo, Japan) and diode laser excitation at 514.6 nm (Fandango, Cobolt AB, Sweden) was used for the hyperspectral Raman imaging of CHIM-treated cells. The incoupling of the laser light occurred *via* a single-mode optical fiber and the excitation power was set to 30 mW before the objective (21 mW at the objective's front lens). A 2D grid of spectra was measured using an integration time of 1 s per pixel and a digital lateral resolution of 2 pixel per μm in both, *x*- and *y*-direction for normal scans and an integration time of 2 s per pixel with 3 pixel per μm for the spatially and spectrally better-resolved scans. The Raman scattered light was collected in epi-direction by the objective and guided by a multimode optical fiber (100 μm) to the spectrometer (UHTS300, WITec, Germany) entrance. The collected light was dispersed by a blazed grating (600 g mm^−1^, BLZ: 500 nm, spectral center: 2099.745 cm^−1^ (580.327 nm) for spectra between −300 cm^−1^ and 3940 cm^−1^ or 1800 g mm^−1^, BLZ: 500 nm, spectral center: 2500.00 cm^−1^ (576.970 nm) for spectra of the silent region) and detected by a back-illuminated CCD camera (DV401A, Andor, UK). The individual spectra were processed in Python, despiked,^85^ background corrected by a SNIP algorithm,^83,84^ and trimmed. The wavenumber axis was calibrated with respect to *N*-(4-hydroxyphenyl)acetamide^86^ prior to further band evaluation.

### Stimulated Raman scattering in primary macrophages

SRS imaging on primary cells was performed using an inverse microscope (DMi8, Leica Microsystems GmbH, Germany) embedded into the commercial multimodal Leica Stellaris 8 CRS imaging system (Leica Microsystems GmbH, Germany). The excitation light was provided by a shot noise limited picoEmerald laser (APE, Germany) applicable for nonlinear imaging tasks including SRS. The Stokes beam emitted from the laser oscillator was fixed at 1031.1 nm with a pulse width of 2 ps while the tunable pump beam (80 MHz) could be adjusted between 700 nm and 990 nm. Spatio- and temporal overlap between both beams was ensured by the laser system. For SRS imaging the Stokes beam was modulated with a frequency of 20 MHz by an EOM included within the picoEmerald. The excitation light was focused onto the sample using a dedicated water immersion IR objective (HC PL IRAPO 40×/1.10 WATER, Leica, Germany). The signal from the sample was collected in the transmission direction by a 1.4 NA oil condenser and SRS was detected in forward direction as pump attenuation by a lock-in detector (gain 10%). Images were measured with a zoom of 5 and a speed of 100 Hz (15.4 μs per px when using 512 px × 512 px for Fig. 5 and the large area scan in Fig. S26†/30.8 μs per px when using 256 px × 256 px for the *z*-scan (Fig. S24†) or the *Λ*-excitation scan (Fig. 5H and S23†)) and a power after leaving the laser outlet of 15 mW for the pump and 30 mW for the Stokes beam. Large area images in the *xy*-plane were constructed using *ImageJ*'s grid stitching plugin.^87^

### Cell treatment

5-Ethinyl-2′-deoxyuridine (EdU) was purchased from Biomol (Caiman Chemicals). Dulbecco's Phosphate-Buffered Saline (DPBS) was obtained from gibco and phosphate-buffered formaldehyde solution for cell fixation (4% formaldehyde) from Roth. Fatty acid-free bovine serum albumin (FAF-BSA) was ordered at Merck. Human embryonic kidney 293 T cells were obtained from CAMR Centre for Applied Microbiology and Research (Porton Down, Salisbury, UK).

#### Single CHIM incubation experiments in HEK cells

Human embryonic kidney 293 T cells (≈35 000 cells per dish) were platted on 35 mm glass bottom dishes (ibidi, #1.5) and cultured in 1 : 1 Dulbecco's Minimal Eagles Medium and Ham's F12 medium (DMEM-F12, Thermo Fischer Scientific) supplemented with 10% fetal bovine serum (FCS) for 3–4 days at 37 °C, 5% CO_2_.

Prior to the treatment with the CHIMs, the cell medium was replaced by serum-free Dulbecco's phosphate-buffered saline (DPBS) and the cells were starved for 20 min on ice. The medium was then replaced by the treatment solution of the following composition.

A transparent 100 μM solution of the respective CHIM was obtained by mixing 10 μL of a 10 mM solution of the CHIM in DMSO with 1 μL MeOH followed by rapid addition of 990 μL of medium (DMEM-F12 + 10% FCS).

After incubating for 20 min on ice, the cells were washed with DPBS (3 × 1 mL) to remove the remaining non-incorporated CHIMs, fixed with formaldehyde solution (1 mL, 4% formaldehyde) for 15 min at room temperature, washed again with (1 × 1 mL DPBS) and stored in DPBS at 4 °C prior to the measurement.

#### Multiplexing experiments in HEK cells

For multiplexing experiments without EdU, the cell density given in the previous section was used. For the multiplexing experiments with EdU, the cell density was lowered (≈12 000 cells per dish) and cells were seeded in glass bottom dishes as described above. The cell medium was then replaced by a 1 mM solution of EdU in 1.4 mL medium and the cells were incubated for 51.5 h at 37 °C, 5% CO_2_.

For the EdU – single CHIM multiplexing experiments the EdU-treatment solution was then removed, the cells were washed (3 × 1 mL DPBS) and the CHIM-uptake method with starvation, incubation on ice, and fixation was followed (*c*_CHIM_ = 100 μM in the cell solution).

For the EdU – double CHIM multiplexing experiments the concentration of CHIM 5 was reduced to 50 μM due to its high scattering cross-section, while the concentration of CHIM 6 remained at 100 μM (starvation, incubation on ice and fixation see above).

#### Experiments with primary macrophages

Macrophages were prepared as previously described.^88^ Briefly, monocytes were isolated from leukocyte concentrates obtained from freshly withdrawn peripheral blood of two human healthy male volunteers (27 years and 54 years old) provided by the Institute of Transfusion Medicine, University Hospital Jena, Germany. Informed consent was obtained from all human subjects. The experimental protocol was approved by the ethical committee of the University Hospital Jena. These subjects had no apparent inflammatory conditions, infections, or current allergic reactions, and had not taken antibiotics or anti-inflammatory drugs for at least 10 days prior to blood collection. Leukocytes were separated using dextran sedimentation of erythrocytes, followed by centrifugation on lymphocyte separation medium (Histopaque®-1077, Sigma-Aldrich). Peripheral blood mononuclear cells were seeded in PBS containing 1 mM CaCl_2_ and 0.5 mM MgCl_2_ in a cell culture flask (650 mL, Greiner Bio-one) and stored in the incubator (37 °C, 5% CO_2_) for 1 h to enrich monocytes by adherence at the flask bottom. All non-adherent cells were removed. RPMI 1640 (Sigma-Aldrich), supplemented with 10% FCS, 2 mmol per L l-glutamine, 100 U per mL penicillin, 100 μg per mL streptomycin (Biochrom/Merck), referred as cell culture medium, was added. Subsequently, 20 ng per mL M-CSF (Peprotech) was added, and cells were incubated for a 6-days differentiation period (37 °C, 5% CO_2_) into M0_M-CSF_ macrophages. Macrophages were harvested with PBS containing 5 mM EDTA (AppliChem), counted, and seeded (0.75 Mio cells/1 mL cell culture medium) on 35 mm glass bottom dishes (ibidi, #1.5). Cells were kept in an incubator (37 °C, 5% CO_2_) for 2 h until further treatment. In total, 4 dishes (2 of each donor) were prepared. One dish of each set was dedicated to the CHIM treatment, while the second dish served as a negative control. 1.22 mg of CHIM 4 were dissolved in 35 μL DMSO resulting in a 48.3 mM stock solution. To prepare a treatment solution with an approximate concentration of 100 μM of CHIM 4 per dish, 3 μL of the stock solution were mixed with 7 μL DMSO and 1 μL MeOH followed by the addition of 1.5 mL cell culture medium. For a treatment under more physiological conditions, cells were starved for 20 min in PBS at 37 °C with 5% CO_2_. After starvation, the cells were incubated with the prepared treatment solution in the incubator (37 °C, 5% CO_2_) for 20 min. The cells were then washed (3×) with PBS, fixed for 15 min with a formaldehyde-containing cell fixing solution (4% formaldehyde), washed again with PBS, and stored in PBS. Cell dishes of the negative controls underwent the same treatment procedure, except that the amount of stock solution was replaced by DMSO in these cases.

### Fluorescence imaging of filipin III-labelled cells

For counterstaining experiments, cells were prepared as described in the subsection “Single CHIM incubation experiments” and stained with filipin following a variation of the procedure of Mukherjee *et al.*^59^ Treated cells were fixed (2 mL, 4% paraformaldehyde) for 20 min at room temperature. Untreated cells were fixed with no additional ice treatment for 1 h at room temperature. The cells were then washed with DPBS, (3 × 1 mL, 1×), the unreacted paraformaldehyde was quenched with glycine (1.5 mL, 1.5 mg mL^−1^) for 20 min at room temperature and the cells were washed (3 × 1 mL, 1×) again and stored in DPBS for Raman imaging.

After Raman imaging, the cells were incubated for 2 h at room temperature in the dark with a solution of 50 μM filipin III in DPBS (prepared from a stock of 25 mg mL^−1^ in DMSO). The cells were washed with DPBS (3 × 1 mL, 1×) and fluorescence images were recorded immediately.

Fluorescence images of filipin III-labelled cells were recorded using a Leica SP8 Falcon and Leica Stellaris 8, both equipped with an inverted microscopy setup (DMi8, Leica Microsystems, Germany). Filipin III was excited by two-photon absorption, either provided by the fully integrated multiphoton laser (Leica Stellaris 8, MP. power: 1.78 W, used: 2.5%) or an external tunable laser (deltaEmerald, APE, Germany coupled to Leica SP8 Falcon, 200 mW output). The excitation light was focused onto the sample with a 63× objective (SP8 Falcon: HC PL APO 63×/1.40 OIL CS2, Leica Microsystems, Germany; Stellaris 8: HC PL APO 63×/1.20 WATER CS2, Leica Microsystems, Germany) The emitted signal (TPEF) was registered with a confocal single photon counting hybrid detector (HyD SMD, 390–600 nm, Leica SP8 Falcon + SP 665 + MP 1680 sp) or an ultrasensitive external photomultiplier tubes (PMT-NDD, 420–558 nm, Leica Stellaris 8 + SP667 + SP680).

If not stated otherwise, images (fluorescence and Raman) shown in this paper are based on at least 2 biological replicates with at least 3 imaged areas each. Incubation experiments with no storage in lipid droplet regions intended were the main focus of this manuscript. Additionally, we checked for different treatment conditions (concentration, temperature, shuttling agents), which we decided not to discuss as a major part of this paper as no severe differences compared to the short-term ice incubation were observed.

Our Raman-tagged CHIM analogs have led to reproducible results for 3 years both as a solid and in solution which is a major benefit compared to filipin III that cannot be stored as an aliquot as the staining quality was already largely reduced after some weeks.

### Cell viability assay

The cell toxicity was assessed using the MTT assay. 1 × 10^4^ HEK 293 cells per well (total volume 100 μL) were seeded on a 96-well plate containing 100 μL DMEM supplemented with 10% FBS for 24 h at 37 °C, 5% CO_2_. Before the treatment with CHIMs (2 or 5), the cell medium was replaced by PBS and the cells were starved for 20 min on ice. The treatment solution of the following composition then replaced the medium.

A transparent 100 μM, 50 μM or 20 μM solution of the respective CHIM was obtained by mixing 20 μL/10 μL/4 μL of a 1 mM stock solution of the CHIM in DMSO with 2 μL/1 μL/0.4 μL MeOH followed by rapid addition of 178 μL/189 μL/195.6 μL of medium (DMEM + 10% FBS), respectively.

After incubating for 20 min on ice, the cells were washed with PBS (3 × 100 μL) to remove the remaining non-incorporated CHIMs. Then, 10 μL of 5 mg mL^−1^ MTT was added to each well and the cells were incubated for 3 h under standard culture conditions. 100 μL of DMSO was added to each well to solubilize the formazan crystals and the absorbance at 570 nm was measured using a multimode plate reader (Spark, Tecan Life Science). After background subtraction, the viability in the absence of CHIMs under otherwise same treatment conditions was set to 100%.

### Integration map scaling

For Fig. 3 and various figures in the ESI† a common scaling was implemented within the RGB images which ensures cross-image comparisons. For this, the area of nuclei (excluding nucleoli) was segmented and the average signal in the segmented areas was calculated. The nuclei channel (red) was then linearly scaled between the value 0.07 and 1.7× the mean signal in the segmented nuclei region (upper border). The retrieved integrated area in the lipid (blue) and alkyne (green) channel was then divided by the mean signal in the segmented nuclei region. The assignment of the dynamic range of the blue colormap (lipids) was done between 0 and 4. The dynamic range of the alkyne colormap is given next to each image.

## Author contributions

C. S. and T. W. contributed equally to this work; synthesis: T. W. C. H., T. G.; Raman measurements: C. S.; cytotoxicity assay: Y. Z.; primary cell treatment: M. W.; writing – original draft: C. S., T. W.; interpretation of measurement data: C. S., M. S; T. M. Z., T. W., Y. Z.; supervision: F. G., M. S., J. P., S. V. W., O. W.; conceptualization: F. G., J. P.; writing – review & editing: all authors, funding acquisition: F. G., J. P.

## Conflicts of interest

There are no conflicts to declare.

## Supplementary Material

SC-015-D4SC03155E-s001

## Data Availability

The data supporting this article have been included as part of the ESI.†
