# Supplementary material for: Alkyne-tagged imidazolium-based membrane cholesterol analogs for Raman imaging applications
Source: Chem Sci. 2024 Aug 7;15(35):14323–35. doi: 10.1039/d4sc03155e (PMC11325193; doi:10.1039/d4sc03155e)
Supplement: SC-015-D4SC03155E-s001 [file SC-015-D4SC03155E-s001.pdf]

Supplementary Information (SI) for Chemical Science.
This journal is © The Royal Society of Chemistry 2024

Alkyne-tagged imidazolium-based membrane cholesterol analogs for Raman imaging applications

Constanze Schultz [a†], Tristan Wegner [b†], Corinna Heusel [b], Tim Gallagher [b], Yanjun Zheng [c], Markus Werner [d], Seraphine V. Wegner [c], Tobias Meyer-Zedler [a,e], Oliver Werz [d], Michael Schmitt [e], Juergen Popp [*a,e] and Frank Glorius [*b]

[a]Leibniz Institute of Photonic Technology (IPHT), Member of Leibniz Health Technologies, Member of the Leibniz Center for Photonics in Infection Research (LPI), Albert-Einstein-Str. 9, 07745 Jena, Germany

[b]University of Münster, Institute of Organic Chemistry, Corrensstraße 40, 48149 Münster, Germany

[c]University of Münster, Institute of Physiological Chemistry and Pathobiochemistry, Waldeyerstraße 15, 48149 Münster, Germany

[d]Department of Pharmaceutical/Medicinal Chemistry, Institute of Pharmacy, Friedrich-Schiller-University Jena, Philosophenweg 14, 07743, Jena, Germanyd

[e]Institute of Physical Chemistry (IPC) and Abbe Center of Photonics (ACP), Member of the Leibniz Center for Photonics in Infection Research (LPI), Friedrich Schiller University Jena, Helmholtzweg 4, 07743 Jena, Germany

† These authors contributed equally to this work.

* Correspondence may be addressed to Frank Glorius (glorius@uni-muenster.de, synthesis) and Juergen Popp (juergen.popp@leibniz-ipht.de, spectroscopy)

CONTENT

- **SUPPORTING METHODS**

I. Synthesis
- 1.1 General information
- 1.2 Synthesis
- 1.3 NMR spectra
- 1.4 References

II. Raman measurements
- 2.1 Additional details on the tags' Raman properties
- 2.2 BCARS measurements
- 2.3 Remarks on the prediction of the vibrational Raman spectra
- 2.4 Labeling experiments with a commercially available tagged cholesterol
- 2.5 Supporting information for Figure 3
- 2.6 Determination of the in-situ limit of detection
- 2.7 Cell viability of selected CHIMs
- 2.8 Selected additional data on incubation experiments with different treatment conditions
- 2.9 Additional data concerning the spatial distribution of CHIMs
- 2.10 Counterstaining experiments with filipin III
- 2.11 Experiments with primary cells
- 2.12 References

SUPPORTING METHODS

I. Synthesis and characterization of Raman-tagged CHIMs

1.1 General information

All reactions were performed under argon. Reaction temperatures are referred to the ones of the heating/cooling media (heating block, cryogenic bath). Reagents were purchased from Sigma-Aldrich, Merck, ACROS Organics, Alfa Aesar, Fluorochem, Combiblocks, TCI and used without further purification. Reactions were stirred using PTFE-coated magnetic stirring bars at ~ 1000 rpm. Low boiling solvents (<110 °C) were removed by rotary evaporation under reduced pressure, heating the solution with a water bath at 50 °C. High boiling solvents (>110 °C) were removed in vacuo (<1 mbar) at room temperature or under mild heating (<60 °C). Identity and purity of synthesized compounds was verified by means of ^{1}H NMR, ^{13}C NMR or ESI-MS (accurate mass determination). Literature known compounds were compared with the available analytical data to confirm their identity.

Thin layer chromatography (TLC) was performed using Merck silica gel 60 F254 aluminum plates and visualization was accomplished with UV light (254 nm) and/or staining with basic $KMnO_4$ solution (4 g of $KMnO_4$, 10 g K_2CO_3, 1 g NaOH in 200 ml of distilled water). ^{1}H and ^{13}C spectra were recorded at room temperature on a Bruker Avance 400 (^{1}H: 400.13 MHz; ^{13}C: 100.62 MHz), Avance Neo 400 (^{1}H: 400.23 MHz; ^{13}C: 100.65 MHz), Varian 500 MHz INOVA (^{1}H: 499.83 MHz; ^{13}C: 125.70 MHz) or Varian Unity plus 600 (^{1}H: 599.31 MHz; ^{13}C: 150.71 MHz) in deuterated solvent (> 99.5 Deuteration) purchased from Sigma-Aldrich ($CDCl_3$). Chemical shifts (*δ*) for ^{1}H and ^{13}C NMR spectra are given in parts per million (ppm) relative to tetramethylsilane (TMS) using the residual solvent signals as references for ^{1}H and ^{13}C NMR spectra ($CDCl_3$: δ_H= 7.26 ppm, δ_C= 77.16 ppm; CD_3OD: δ_H= 3.31 ppm, δ_C= 49.00 ppm). NMR signals multiplicities that can be analyzed as first order multiplets are reported using the following abbreviations (or combination thereof): s = singlet, d = doublet, t = triplet, q = quartet, m = multiplet, b = broad signal. All spectra were processed using MestReNova 14 using standard phase and baseline correction automations. ESI accurate mass spectra were recorded on a TQ Orbitap LTQ XL (Thermo-Fisher Scientific, Bremen) with loop injection or an Orbitrap Velos Pro (Thermo-Fisher Scientific, Bremen) with nanospray injection.

Compounds were purified by flash column chromatography using ACROS Organics silica (0.035-0.070 mm, 60 Å) and the specified solvent system under 0.3–0.5 bar argon overpressure. Pentane, ethyl acetate, dichloromethane, diethyl ether (Et_2O) and acetone used for chromatography or extraction were purchased of technical grade and preliminary purified by atmospheric pressure distillation. Dichloromethane (DCM) used as reaction solvent was purified using a custom SPS with activated alumina columns (built by the "Feinmechanische Werkstatt des Organisch-Chemischen Instituts, WWU Münster") and collected under positive argon pressure. EtOH, MeOH, chloroform ($CHCl_3$), 1,4-dioxane, acetonitrile (MeCN) and DMSO were purchased of extra pure/analytical grade (>99% purity) and used without further purification.

1.2 Synthesis

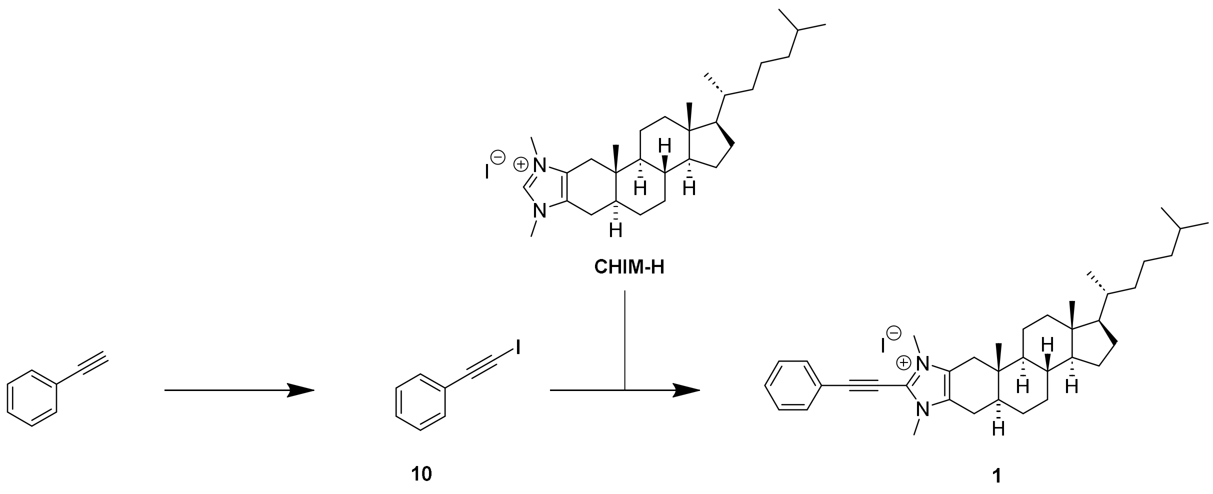

Scheme S1. Synthesis of CHIM derivative **1**.

CHIM-H

CHIM-H was synthesized according to a literature procedure.[1]

(Iodoethynyl)benzene (10)

Compound **10** was synthesized according to a literature procedure starting from commercially available ethynylbenzene.[2]

CHIM derivative 1

Compound **1** was synthesized based on a modified literature procedure.[3] **CHIM-H** (90 mg, 0.16 mmol, 1.0 equiv.), alkyne **10** (81 mg, 0.36 mmol, 2.3 equiv.), Cu_2O (5 mg, 0.03 mmol, 0.2 equiv.), NaOAc (14 mg, 0.17 mmol, 1.1 equiv.) and 1,4-dioxane (1.8 mL) were added to a Schlenk tube and stirred at 120 °C overnight. The solvent was removed under reduced pressure and the crude product was subjected to flash column chromatography (DCM/MeOH : 100/0 → 90/10) to yield compound **1** as a brown solid (22 mg, 0.033 mmol, 21 %).

^{1}H-NMR (600 MHz, $CDCl_3$): δ (ppm) = 7.68–7.63 (m, 2H), 7.54–7.49 (m, 1H), 7.46–7.41 (m, 2H), 3.93–3.82 (m, 6H), 2.76–2.69 (m, 1H), 2.65–2.58 (m, 1H), 2.48–2.41 (m, 1H), 2.32–2.24 (m, 1H), 2.05–2.00 (m, 1H), 1.85–1.76 (m, 2H), 1.73–1.66 (m, 2H), 1.62–0.94 (m, 27H), 0.91 (d, J = 6.5 Hz, 4H), 0.88–0.84 (m, 11H), 0.67 (s, 3H).

^{13}C{^{1}H}-NMR (150 MHz, $CDCl_3$): δ (ppm) = 132.6, 131.6, 131.3, 130.3, 129.0, 128.1, 118.9, 106.8, 70.5, 56.3, 56.1, 53.1, 42.5, 41.3, 39.8, 39.6, 37.0, 36.3, 35.9, 35.5, 34.6, 34.2, 34.1, 31.3, 28.6, 28.3, 28.1, 25.1, 24.3, 24.0, 23.0, 22.7, 21.5, 18.8, 12.9, 12.1.

ESI-MS: *m/z* calculated $[C_{38}H_{55}N_2]^+$: 539.4360, found: 539.4369.

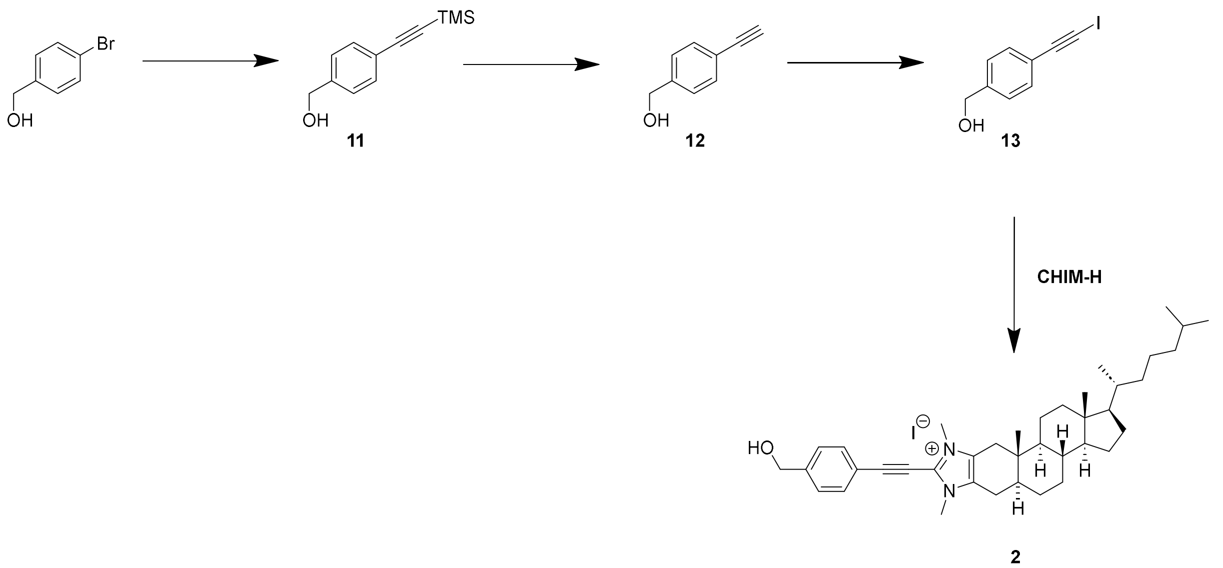

Scheme S2. Synthesis of CHIM derivative **2**.

(4-(Iodoethynyl)phenyl)methanol (13)

Compound **11** and **12** were synthesized according to a literature procedure starting from commercially available (4-bromophenyl)methanol.[4] Compound **13** was synthesized was synthesized based on a literature procedure.[2]

CHIM derivative 2

Compound **2** was synthesized based on a modified literature procedure.[3] **CHIM-H** (80 mg, 0.14 mmol, 1.0 equiv.), diyne **13** (80 mg, 0.31 mmol, 2.2 equiv.), Cu_2O (4 mg, 0.03 mmol, 0.2 equiv.), NaOAc (12 mg, 0.15 mmol, 1.0 equiv.) and 1,4-dioxane (1.6 mL) were added to a Schlenk tube and stirred at 120 °C overnight. The solvent was removed under reduced pressure and the crude product was subjected to flash column chromatography (DCM/MeOH : 100/0 → 90/10) to yield compound **2** as a yellow/brown solid (22 mg, 0.032 mmol, 23 %).

^{1}H-NMR (500 MHz, $CDCl_3$): δ (ppm) = 7.64 (d, J = 8.0 Hz, 2H), 7.44 (d, J = 7.9 Hz, 2H), 4.76 (s, 2H), 3.91–3.81 (m, 6H), 2.74–2.67 (m, 1H), 2.64–2.56 (m, 1H), 2.46–2.42 (m, 1H), 2.31–2.23 (m, 1H), 2.05–2.00 (m, 1H), 1.87–1.77 (m, 2H), 1.73–1.67 (m, 2H), 1.61–0.95 (m, 28H), 0.92 (d, J = 6.5 Hz, 3H), 0.88–0.84 (m, 10H), 0.68 (s, 3H).

^{13}C{^{1}H}-NMR (125 MHz, $CDCl_3$): δ (ppm) = 145.2, 132.8, 131.2, 130.2, 128.3, 127.2, 117.6, 107.1, 70.5, 64.4, 56.3, 56.1, 53.1, 42.5, 41.3, 39.8, 39.7, 37.0, 36.3, 35.9, 35.6, 34.6, 34.2, 34.1, 31.3, 29.8, 28.6, 28.3, 28.2, 25.1, 24.4, 24.0, 23.0, 22.7, 21.5, 18.8, 12.9, 12.1.

ESI-MS: m/z calculated $[C_{39}H_{57}N_2O]^+$: 569.4465, found: 569.4483.

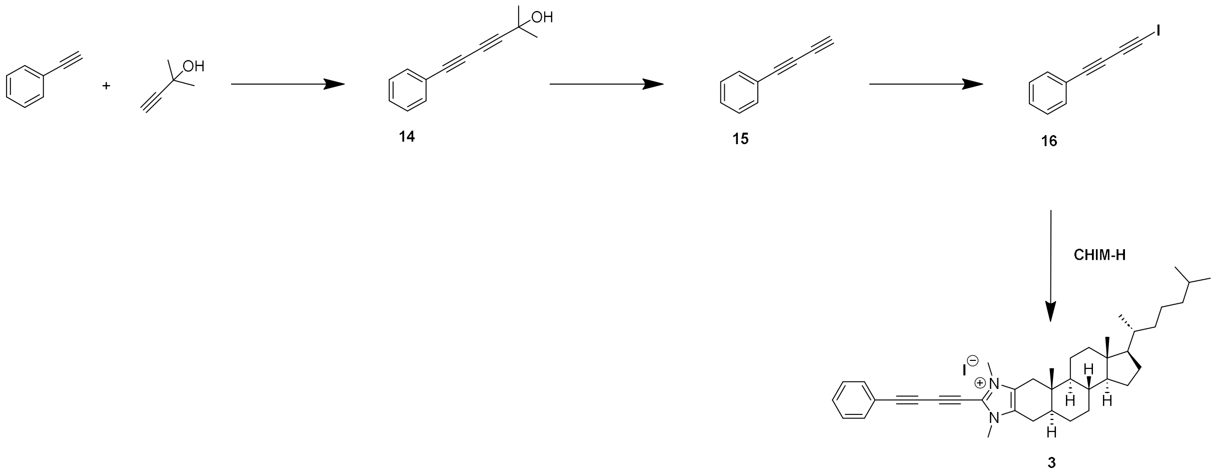

Scheme S3. Synthesis of CHIM derivative 3.

(Iodobuta-1,3-diyn-1-yl)benzene (16)

Compounds **14**, **15** and **16** were synthesized according to literature procedures starting from commercially available ethynylbenzene and 2-methylbut-3-yn-2-ol.[5][6][7]

CHIM derivative 3

Compound **3** was synthesized based on a modified literature procedure.[3] **CHIM-H** (80 mg, 0.14 mmol, 1.0 equiv.), diyne **16** (80 mg, 0.32 mmol, 2.3 equiv.), Cu_2O (5 mg, 0.03 mmol, 0.2 equiv.), NaOAc (14 mg, 0.15 mmol, 1.1 equiv.) and 1,4-dioxane (1.6 mL) were added to a Schlenk tube and stirred at 120 °C overnight. The solvent was removed under reduced pressure and the crude product was subjected to flash column chromatography (DCM/MeOH : 100/0 → 90/10) to yield compound **3** as a brown solid (29 mg, 0.042 mmol, 30 %).

^{1}H-NMR (600 MHz, $CDCl_3$): *δ* (ppm) = 7.63–7.56 (m, 2H), 7.51–7.47 (m, 1H), 7.43–7.38 (m, 2H), 3.96–3.78 (m, 6H), 2.78–2.69 (m, 1H), 2.64–2.56 (m, 1H), 2.51–2.44 (m, 1H), 2.30–2.22 (m, 1H), 2.04–2.00 (m, 1H), 1.86–1.80 (m, 2H), 1.73–1.66 (m, 2H), 1.60–0.96 (m, 29H), 0.92 (d, *J* = 6.5 Hz, 4H), 0.89–0.84 (m, 11H), 0.67 (s, 3H).

^{13}C{^{1}H}-NMR (150 MHz, $CDCl_3$): *δ* (ppm) = 133.3, 132.2, 131.1, 131.1, 128.9, 127.4, 119.6, 91.3, 90.5, 72.0, 61.0, 56.3, 56.1, 52.8, 42.5, 41.1, 39.7, 39.7, 37.0, 36.3, 35.9, 35.6, 34.7, 34.5, 34.4, 31.2, 29.8, 28.6, 28.3, 28.2, 25.2, 24.4, 24.0, 23.0, 22.7, 21.5, 18.8, 13.1, 12.1.

ESI-MS: *m/z* calculated $[C_{40}H_{55}N_2]^+$: 563.4360, found: 563.4355.

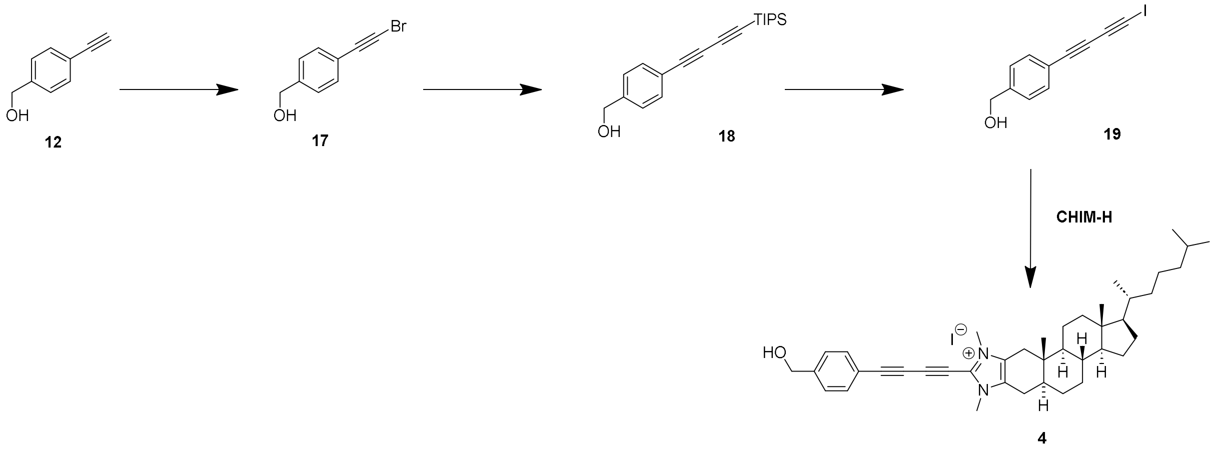

Scheme S4. Synthesis of CHIM derivative **4**.

(4-(Bromoethynyl)phenyl)methanol (17)

Alkyne **12** (1.1 g, 8.5 mmol, 1.0 equiv.), $AgNO_3$ (145 mg, 0.854 mmol, 0.1 equiv.) and dry acetone (28 mL) were added to a Schlenk tube wrapped in aluminum foil. After stirring at room temperature for 1 h *N*-bromo succinimide (2.3 g, 13 mmol, 1.5 equiv.) was added. After additional 30 minutes of stirring another 0.1 equiv. of $AgNO_3$ was added. The reaction mixture was stirred for another 3 hours at rt. Afterwards, the solvent was removed under removed pressure and the crude product was subjected to flash column chromatography (pentane/EtOAc : 5/2) to yield compound **17** as a yellow solid (1.3 g, 6.2 mmol, 72 %).

^{1}H-NMR (400 MHz, $CDCl_3$): δ (ppm) = 7.50–7.27 (m, 4H), 4.69 (d, J = 4.0 Hz, 2H), 1.77–1.68 (m, 1H).

^{13}C{^{1}H}-NMR (101 MHz, $CDCl_3$): δ (ppm) = 141.6, 132.3, 126.9, 122.0, 80.0, 65.0, 49.9.

(4-((Triisopropylsilyl)buta-1,3-diyn-1-yl)phenyl)methanol (18)

CuCl (13 mg, 0.13 mmol, 0.02 equiv.) was added to a solution of isopentylamine (1.7 mL) in dest. H_2O (4.5 mL) resulting in a blue solution. Subsequently, a spatula tip of [(NH_3OH)Cl] was added resulting in a change of the color of the solution from blue to colorless. Triisopropyl acetylene (1.7 mL, 7.6 mmol, 1.2 equiv.) was added and the reaction mixture was cooled to 0 °C (ice bath). Bromoalkyne **17** (1.3 g, 6.2 mmol, 1.0 equiv.) was added and the ice bath was removed. Another spatula tip of [(NH_3OH)Cl] was added and the reaction mixture was stirred for 30 minutes at rt. The reaction mixture was extracted with Et_2O and the combined organic phases were dried over $MgSO_4$. Excess solvent was removed under reduced pressure and the crude product was subjected to flash column chromatography (pentane/EtOAc : 5/2) to yield compound **18** as a yellow oil (1.0 g, 4.7 mmol, 76 %).

^{1}H-NMR (400 MHz, $CDCl_3$): δ (ppm) = 7.56–7.27 (m, 4H), 4.70 (d, J = 4.8 Hz, 2H), 1.81–1.70 (m, 1H), 1.11 (s, 18H).

4-(Iodobuta-1,3-diyn-1-yl)phenyl)methanol (19)

TIPS-protected diyne **18** (1.0 g, 3.2 mmol, 1.0 equiv.) was dissolved in MeCN (75 mL) and *N*-iodo succinimide (870 mg, 3.84 mmol, 1.2 equiv.) was added. The reaction vessel was wrapped in aluminum foil and AgF (510 mg, 4.02 mmol, 1.3 equiv.) was added. The reaction mixture was stirred for 5 h at rt. 50 mL CH_2CL_2 were added and the reaction mixture was filtered over Celite. Excess solvente was removed under reduced pressure and the crude product was subjected to flash column chromatography (pentane/EtOAc : 5/2) to yield compound **19** as an off-white solid (554 mg, 1.96 mmol, 61 %).

^{1}H-NMR (400 MHz, $CDCl_3$): δ (ppm) = 7.53–7.27 (m, 4H), 4.70 (s, 2H), 1.78 (bs, 1H).

^{13}C{^{1}H}-NMR (101 MHz, $CDCl_3$): δ (ppm) = 142.5, 133.4, 126.9, 120.1, 78.4, 75.1, 73.7, 64.9.

CHIM derivative 4

Compound **4** was synthesized based on a modified literature procedure.[3] **CHIM-H** (160 mg, 0.282 mmol, 1.0 equiv.), diyne **19** (160 mg, 0.567 mmol, 2.0 equiv.), Cu_2O (8 mg, 0.06 mmol, 0.2 equiv.), NaOAc (24 mg, 0.29 mmol, 1.0 equiv.) and 1,4-dioxane (3.2 mL) were added to a Schlenk tube and stirred at 120 °C overnight. The solvent was removed under reduced pressure and the crude product was subjected to flash column chromatography (DCM/MeOH : 100/0 → 90/10) to yield compound **4** as a brown solid (33 mg, 0.046 mmol, 16 %).

^{1}H-NMR (500 MHz, $CDCl_3$): δ (ppm) = 7.56 (d, J = 8.0 Hz, 2H), 7.40 (d, J = 7.9 Hz, 2H), 4.73 (s, 2H), 3.86–3.81 (m, 6H), 2.76–2.67 (m, 1H), 2.63–2.57 (m, 1H), 2.49–2.43 (m, 1H), 2.30–2.22 (m, 1H), 2.04–1.99 (m, 1H), 1.84–1.77 (m, 2H), 1.73–1.66 (m, 2H), 1.60–0.96 (m, 32H), 0.91 (d, J = 6.5 Hz, 4H), 0.88–0.84 (m, 12H), 0.67 (s, 3H).

^{13}C{^{1}H}-NMR (125 MHz, $CDCl_3$): δ (ppm) = 144.8, 133.3, 132.1, 131.0, 127.4, 127.2, 118.3, 91.2, 90.6, 71.8, 64.4, 61.0, 56.3, 56.1, 53.0, 42.5, 41.2, 39.7, 39.7, 37.0, 36.3, 35.9, 35.5, 34.6, 34.4, 34.3, 31.2, 28.6, 28.3, 28.2, 25.2, 24.4, 24.0, 23.0, 22.7, 21.5, 18.8, 13.0, 12.1.

ESI-MS: *m/z* calculated $[C_{41}H_{57}N_2O]^+$: 593.4465, found: 593.4474.

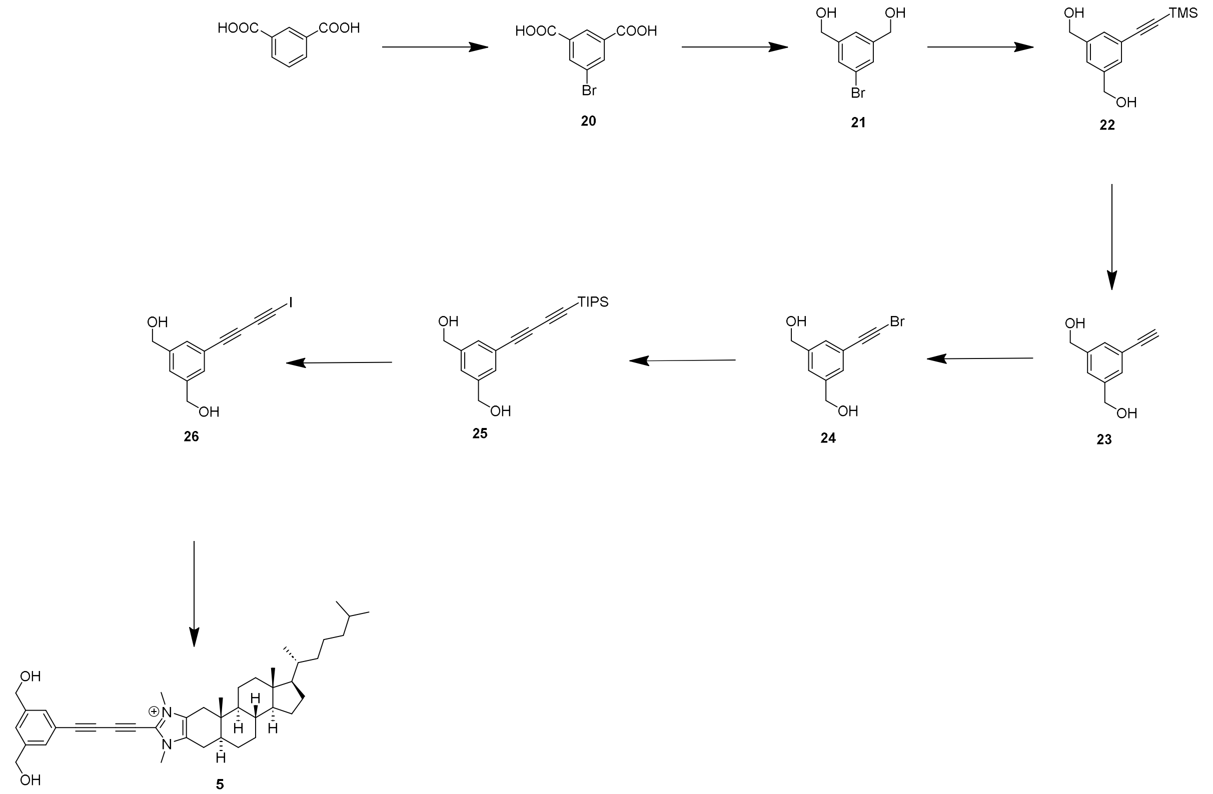

Scheme S5. Synthesis of CHIM derivative **5**.

(5-Bromo-1,3-phenylene)dimethanol (21)

Compound **20** and **21** were synthesized according to literature procedures starting from commercially available isophthalic acid.[8][9]

(5-((Trimethylsilyl)ethynyl)-1,3-phenylene)dimethanol (22)

Compound **22** was synthesized based on a literature procedure.[4]

^{1}H-NMR (400 MHz, CD_3OD): δ (ppm) = 7.32 (s, 3H), 4.57 (s, 4H), 0.22 (s, 9H).

^{13}C{^{1}H}-NMR (101 MHz, CD_3OD): δ (ppm) = 143.4, 130.0, 126.7, 124.5, 106.4, 94.1, 64.6, 0.0.

(5-Ethynyl-1,3-phenylene)dimethanol (23)

Compound **23** was synthesized based on a literature procedure.[4]

^{1}H-NMR (400 MHz, CD_3OD): δ (ppm) = 7.39–7.32 (m, 3H), 4.59 (s, 4H), 3.45 (s, 1H).

^{13}C{^{1}H}-NMR (101 MHz, CD_3OD): δ (ppm) = 143.4, 130.2, 126.8, 123.8, 84.5, 78.3, 64.6.

(5-(Bromoethynyl)-1,3-phenylene)dimethanol (24)

Compound **24** was synthesized as described for compound **17**.

^{1}H-NMR (400 MHz, CD_3OD): *δ* (ppm) = 7.37–7.26 (m, 3H), 4.57 (s, 4H).

^{13}C{^{1}H}-NMR (101 MHz, CD_3OD): *δ* (ppm) = 143.5, 130.0, 126.9, 124.0, 80.9, 64.5, 50.6.

(5-((Triisopropylsilyl)buta-1,3-diyn-1-yl)-1,3-phenylene)dimethanol (25)

Compound **25** was synthesized as described for compound **18**.

^{1}H-NMR (400 MHz, CD_3OD): *δ* (ppm) = 7.42–7.33 (m, 3H), 4.57 (s, 4H), 1.11 (s, 21H).

^{13}C{^{1}H}-NMR (101 MHz, CD_3OD): *δ* (ppm) = 143.8, 130.7, 127.6, 122.5, 90.9, 87.9, 76.8, 74.6, 64.5, 19.0, 12.5.

(5-(Iodobuta-1,3-diyn-1-yl)-1,3-phenylene)dimethanol (26)

Compound **26** was synthesized as described for compound **19**.

^{1}H-NMR (400 MHz, CD_3OD): *δ* (ppm) = 7.35 (s, 3H), 4.55 (s, 4H).

^{13}C{^{1}H}-NMR (101 MHz, CD_3OD): *δ* (ppm) = 143.7, 130.9, 127.6, 122.2, 77.9, 75.3, 73.8, 64.4, 9.5.

CHIM derivative 5

Compound **5** was synthesized based on a modified literature procedure.[3] **CHIM-H** (50 mg, 0.09 mmol, 1.0 equiv.), diyne **26** (210 mg, 0.673 mmol, 7.5 equiv.), Cu_2O (9 mg, 0.06 mmol, 0.7 equiv.), NaOAc (32 mg, 0.39 mmol, 4.3 equiv.) and 1,4-dioxane (1.5 mL) were added to a Schlenk tube and stirred at 90 °C overnight. The solvent was removed under reduced pressure and the crude product was subjected to flash column chromatography (DCM/MeOH : 100/0 → 90/10) to yield compound **5** as a yellow/brown solid (29 mg, 0.046 mmol, 51 %).

^{1}H-NMR (600 MHz, $CDCl_3$): *δ* (ppm) = 7.36 (s, 2H), 7.32 (s, 1H), 4.52 (s, 4H), 3.81–3.72 (m, 6H), 2.68–2.53 (m, 2H), 2.36–2.29 (m, 1H), 2.29–2.18 (m, 1H), 2.05–1.98 (m, 1H), 1.85–1.79 (m, 1H), 1.72–0.96 (m, 27H), 0.92–0.89 (m, 4H), 0.87–0.83 (m, 10H), 0.66 (s, 3H).

^{13}C{^{1}H}-NMR (150 MHz, $CDCl_3$): *δ* (ppm) = 142.6, 131.9, 130.9, 130.0, 128.1, 127.2, 119.0, 90.9, 71.8, 63.3, 61.3, 56.4, 56.2, 53.1, 42.5, 41.3, 39.8, 39.7, 37.0, 36.3, 36.0, 35.5, 34.6, 34.5, 32.1, 31.3, 29.8, 28.6, 28.4, 28.2, 25.2, 24.4, 24.1, 23.0, 22.7, 21.5, 18.9, 14.2, 13.0, 12.2.

ESI-MS: *m/z* calculated $[C_{42}H_{59}N_2O_2]^+$: 623.45711, found: 523.45641.

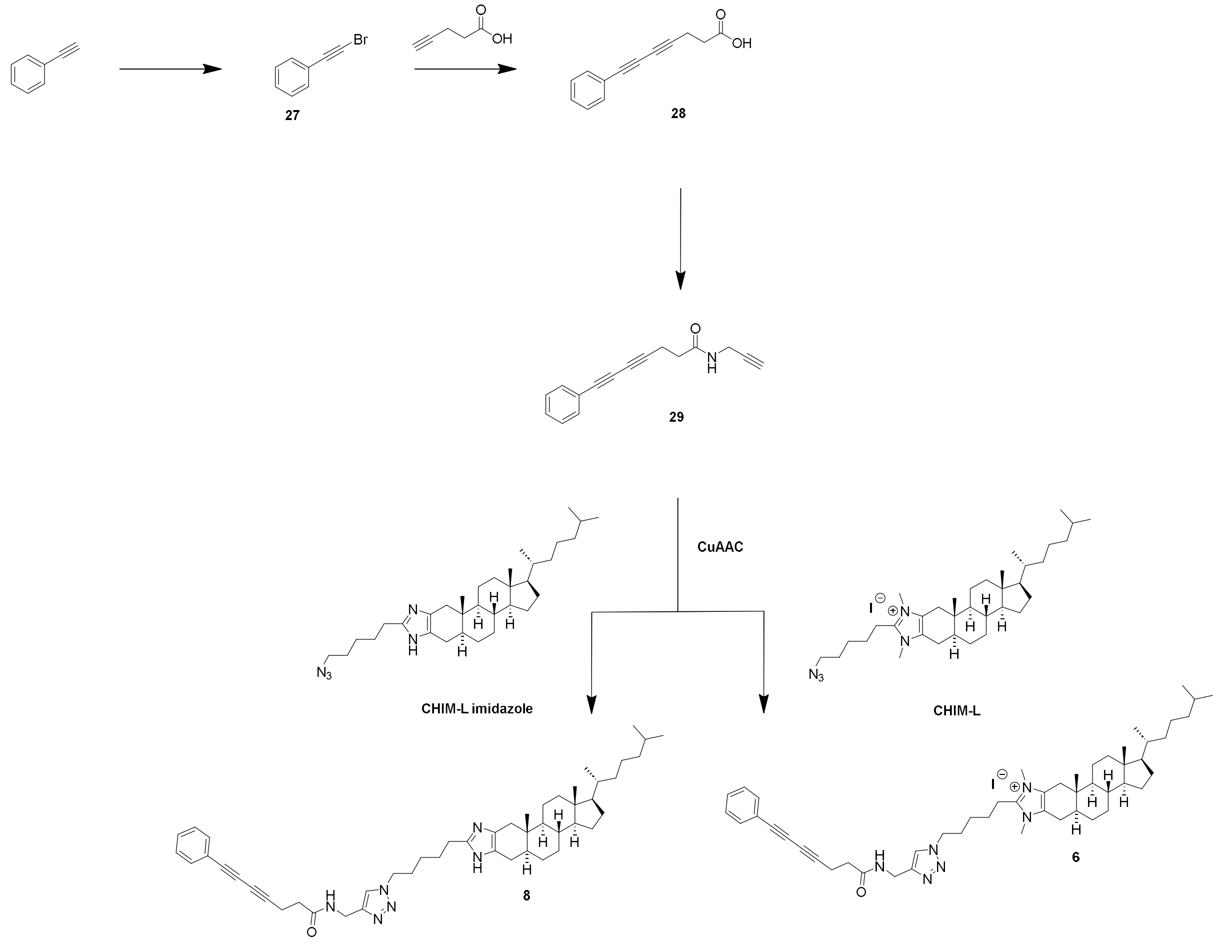

Scheme S6. Synthesis of CHIM derivatives **6** and **8**.

(Bromoethynyl)benzene (27)

Compound **27** was synthesized according to a literature procedure.[10]

7-Phenylhepta-4,6-diynoic acid (28)

Compound **28** was synthesized according to a literature procedure.[11]

7-Phenyl-N-(prop-2-yn-1-yl)hepta-4,6-diynamide (29)

Acid **28** (150 mg, 0.757 mmol, 1.0 equiv) was dissolved in DCM (5 mL). Propargylamine (146 µL, 2.28 mmol, 3.0 equiv.) and 1-Ethyl-3-(3-dimethylaminopropyl)carbodiimide (EDC) (219 mg, 1.14 mmol, 1.5 equiv) were added and the reaction mixture was stirred overnight at room temperature. The solvent was removed under reduced pressure and the crude product was subjected to flash column chromatography (pentane/EtOAc : 2/1) to yield compound **29** as an off-white solid (139 mg, 0.591 mmol, 78 %).

^{1}H-NMR (400 MHz, $CDCl_3$): δ (ppm) = 7.49–7.44 (m, 2H), 7.37–7.27 (m, 3H), 5.92 (s, 1H), 4.11–4.05 (m, 2H), 2.73 (t, J = 7.3 Hz, 2H), 2.48 (t, J = 7.3 Hz, 2H), 2.25 (t, J = 2.6 Hz, 1H).

^{13}**C{^{1}H}-NMR** (101 MHz, $CDCl_3$): δ (ppm) = 170.3, 132.6, 129.2, 128.5, 121.9, 82.5, 79.4, 75.6, 74.1, 71.9, 66.2, 34.8, 29.5, 16.0.
ESI-MS: *m/z* calculated $[C_{16}H_{13}NONa]^+$: 258.08894, found: 258.08879.

CHIM derivative 6

CHIM-L (40 mg, 0.059 mmol, 1.0 equiv.), $CuSO_4{\cdot}5H_2O$ (2.5 mg, 0.010 mmol, 0.17 equiv.) and sodium ascorbate (6 mg, 0.030 mmol, 0.52 equiv.) were added to a mixture of $CHCl_3$ (1.6 mL) and dest. H_2O (2.4 mL). Compound **29** (21 mg, 0.09 mmol, 1.5 equiv.) was added and the reaction mixture was stirred at room temperature overnight. DCM (20 mL) and sat. NH_4Cl solution (20 mL) were added and the phases separated. The aqueous phase was extracted with 2x 20 mL DCM. The combined organic phases were washed with 2x 40 mL 0.1 M EDTA-Na_4 solution and the solvent was removed under reduced pressure. The crude product was subjected to flash column chromatography (DCM/MeOH : 100/0 → 90/10) to yield compound **6** as an off-white solid (13 mg, 0.014 mmol, 24 %).

^{1}**H-NMR** (400 MHz, $CDCl_3$): δ (ppm) = 7.86 (s, 1H), 7.53–7.46 (m, 1H), 7.45–7.41 (m, 2H), 7.35–7.27 (m, 3H), 4.54 (d, *J* = 5.5 Hz, 1H), 4.35 (t, *J* = 6.4 Hz, 2H), 3.75–3.63 (m, 6H), 3.06 (t, *J* = 7.8 Hz, 1H), 2.75–2.68 (m, 2H), 2.64–2.58 (m, 2H), 2.53–2.45 (m, 2H), 2.24–2.11 (m, 2H), 2.04–0.97 (m, 37H), 0.92–0.84 (m, 12H), 0.79 (s, 3H), 0.66 (s, 3H).

^{13}**C{^{1}H}-NMR** (101 MHz, $CDCl_3$): δ (ppm) = 171.2, 145.5, 145.2, 132.6, 129.1, 128.7, 128.6, 127.7, 123.2, 122.0, 83.7, 75.1, 74.6, 65.7, 56.3, 53.4, 49.8, 42.5, 41.7, 39.8, 39.6, 37.0, 36.2, 35.9, 35.6, 35.5, 34.7, 34.2, 32.9, 32.8, 31.4, 29.8, 29.3, 28.6, 28.3, 28.1, 26.4, 26.0, 24.6, 24.3, 24.3, 24.0, 23.0, 22.7, 21.5, 18.8, 16.2, 12.4, 12.1.
ESI-MS: *m/z* calculated $[C_{51}H_{73}N_6O]^+$: 785.58404, found: 785.58428.

CHIM-H imidazole

CHIM-H imidazole was synthesized according to a literature procedure.[1]

CHIM derivative 8

CHIM-L imidazole (31 mg, 0.059 mmol, 1.0 equiv.), $CuSO_4{\cdot}5H_2O$ (2.5 mg, 0.010 mmol, 0.17 equiv.) and sodium ascorbate (6 mg, 0.030 mmol, 0.52 equiv.) were added to a mixture of $CHCl_3$ (1.6 mL) and dest. H_2O (2.4 mL). Compound **29** (21 mg, 0.09 mmol, 1.5 equiv.) was added and the reaction mixture was stirred at room temperature overnight. DCM (20 mL) and sat. NH_4Cl solution (20 mL) were added and the phases separated. The aqueous phase was extracted with 2x 20 mL DCM. The combined organic phases were washed with 2x 40 mL 0.1 M EDTA-Na_4 solution and the solvent was removed under reduced pressure. The crude product was subjected to flash column chromatography (DCM/MeOH : 100/0 → 90/10) to yield compound **8** as an off-white solid (30 mg, 0.040 mmol, 68 %).

^{1}**H-NMR** (400 MHz, $CDCl_3$): δ (ppm) = 8.10 (t, *J* = 5.8 Hz, 1H), 7.78 (s, 1H), 7.43–7.27 (m, 5H), 4.52 (d, *J* = 5.6 Hz, 1H), 4.25 (t, *J* = 6.7 Hz, 2H), 2.85 (t, *J* = 7.5 Hz, 2H), 2.73–2.67 (m, 2H), 2.64–2.50 (m, 4H), 2.28–2.15 (m, 2H), 2.01–1.93 (m, 1H), 1.84–0.91 (m, 34H), 0.91–0.80 (m, 12H), 0.74 (s, 3H), 0.64 (s, 3H).

^{13}C{^{1}H}-NMR (101 MHz, $CDCl_3$): δ (ppm) = 171.7, 145.5, 145.2, 132.6, 129.3, 128.6, 126.7, 125.6, 123.1, 121.7, 83.2, 75.4, 74.3, 65.9, 56.3, 53.6, 50.0, 42.5, 42.1, 39.9, 39.6, 36.9, 36.3, 35.9, 35.6, 35.0, 34.6, 31.6, 29.8, 29.4, 28.8, 28.3, 28.1, 27.3, 25.9, 25.7, 25.4, 24.3, 23.9, 23.2, 23.0, 22.7, 21.4, 18.9, 16.2, 12.1, 12.0.

ESI-MS: *m/z* calculated $[C_{49}H_{68}N_6OH]^+$: 757.55274, found: 757.55331.

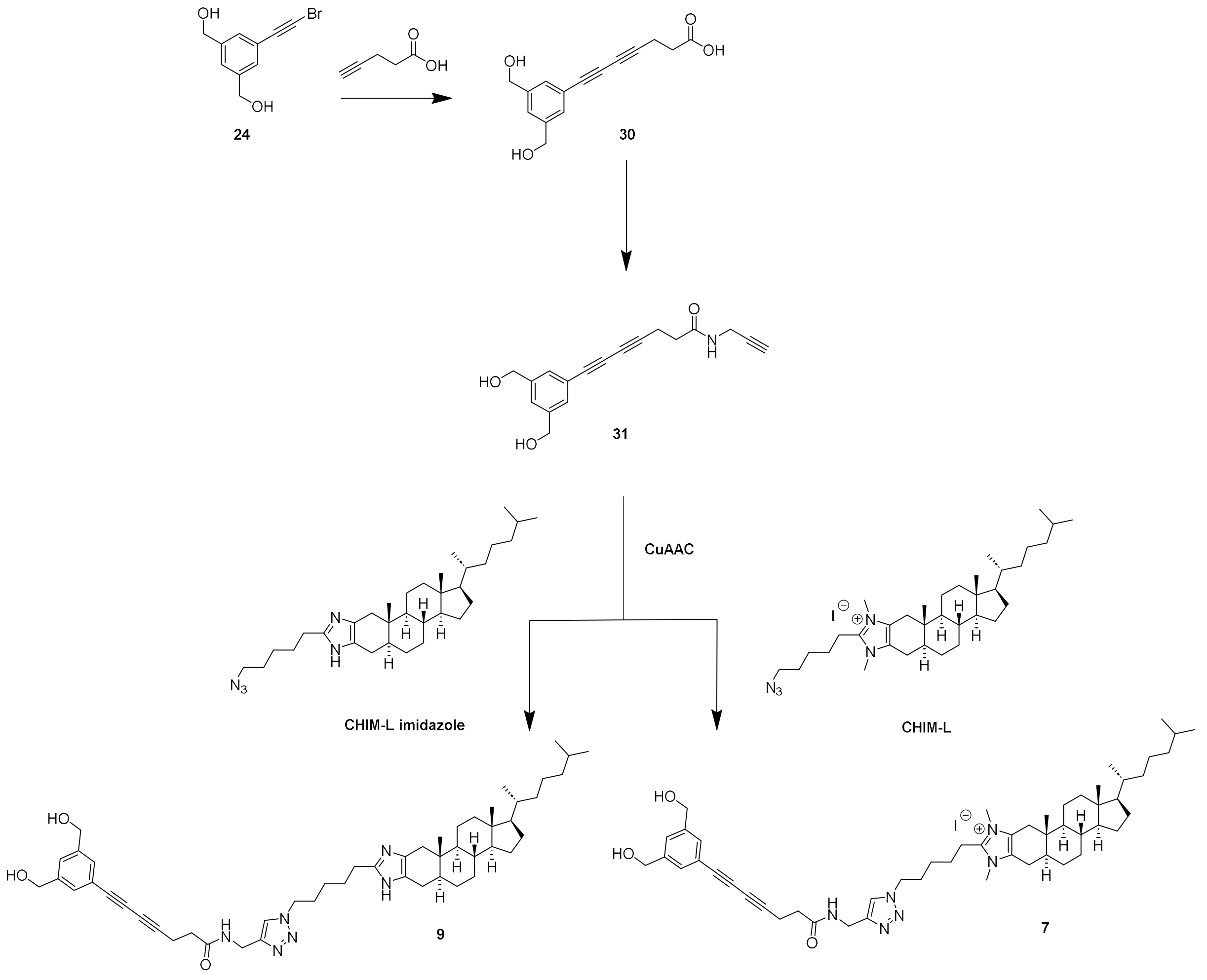

Scheme S7. Synthesis of CHIM derivatives **7** and **9**.

7-(3,5-Bis(hydroxymethyl)phenyl)hepta-4,6-diynoic acid (30)

Compound **30** was synthesized based on a literature procedure.[11]

^{1}H-NMR (400 MHz, CD_3OD): δ (ppm) = 7.35 (s, 3H), 4.58 (s, 4H), 2.74–2.45 (m, 2H).

^{13}C{^{1}H}-NMR (101 MHz, CD_3OD): δ (ppm) = 143.6, 130.5, 127.2, 123.1, 83.7, 75.9, 74.6, 66.1, 64.5, 16.3.

ESI-MS: *m/z* calculated $[C_{15}H_{14}O_4Na]^+$: 281.07843, found: 281.07841.

7-(3,5-Bis(hydroxymethyl)phenyl)-N-(prop-2-yn-1-yl)hepta-4,6-diynamide (31)

Compound **31** was synthesized as described for compound **29**.

^{1}H-NMR (400 MHz, CD_3OD): *δ* (ppm) = 7.35 (s, 3H), 4.58 (s, 4H), 3.98 (d, *J* = 2.6 Hz, 2H), 2.67 (t, *J* = 7.0 Hz, 2H), 2.58 (t, *J* = 2.6 Hz, 1H), 2.46 (t, *J* = 7.2 Hz, 2H).

^{13}C{^{1}H}-NMR (101 MHz, CD_3OD): *δ* (ppm) = 173.2, 143.6, 130.5, 127.2, 123.1, 83.4, 76.0, 74.6, 72.2, 66.4, 64.5, 35.2, 29.5, 16.5.

ESI-MS: *m/z* calculated $[C_{18}H_{17}NO_3Na]^+$: 318.11006, found: 318.10990.

CHIM derivative 7

CHIM-L (40 mg, 0.059 mmol, 1.0 equiv.), $CuSO_4{\cdot}5H_2O$ (2.5 mg, 0.010 mmol, 0.17 equiv.) and sodium ascorbate (6 mg, 0.030 mmol, 0.52 equiv.) were added to a mixture of $CHCl_3$ (1.6 mL) and dest. H_2O (2.4 mL). Compound **31** (18 mg, 0.061 mmol, 1.0 equiv.) was added and the reaction mixture was stirred at room temperature overnight. DCM (20 mL) and sat. NH_4Cl solution (20 mL) were added and the phases separated. The aqueous phase was extracted with 2x 20 mL DCM. The combined organic phases were washed with 2x 40 mL 0.1 M EDTA-Na_4 solution and the solvent was removed under reduced pressure. The crude product was subjected to flash column chromatography (DCM/MeOH : 100/0 → 90/10) to yield compound **7** as an off-white solid (13 mg, 0.013 mmol, 22 %).

^{1}H-NMR (500 MHz, CD_3OD): *δ* (ppm) = 7.89 (s, 1H), 7.39 (s, 3H), 4.62 (s, 4H), 4.50 (s, 2H), 4.42 (t, *J* = 6.8 Hz, 2H), 3.71–3.61 (m, 6H), 2.99 (t, *J* = 7.8 Hz, 2H), 2.76–2.60 (m, 4H), 2.53 (t, *J* = 7.0 Hz, 1H), 2.33–2.22 (m, 2H), 2.15–2.10 (m, 1H), 1.97–1.86 (m, 3H), 1.83–1.77 (m, 1H), 1.76–1.03 (m, 31H), 0.99 (d, *J* = 6.5 Hz, 4H), 0.92 (dd, *J* = 6.6, 1.9 Hz, 7H), 0.86 (s, 3H), 0.77 (s, 3H).

^{13}C{^{1}H}-NMR (125 MHz, CD_3OD): *δ* (ppm) = 173.6, 146.6, 146.4, 143.8, 130.4, 129.7, 128.8, 127.3, 124.2, 122.9, 83.9, 76.1, 74.7, 66.5, 64.4, 57.6, 57.6, 54.8, 50.9, 43.6, 43.0, 41.2, 40.7, 37.9, 37.3, 37.1, 36.8, 35.9, 35.3, 35.0, 32.6, 32.2, 32.1, 30.6, 29.7, 29.5, 29.3, 29.1, 26.9, 26.7, 25.2, 25.2, 24.9, 24.0, 23.2, 22.9, 22.4, 19.2, 16.8, 12.4, 12.3.

ESI-MS: *m/z* calculated $[C_{53}H_{77}N_6O_3]^+$: 845.60517, found: 845.60423.

CHIM derivative 9

CHIM-L imidazole (36 mg, 0.069 mmol, 1.0 equiv.), $CuSO_4{\cdot}5H_2O$ (2.9 mg, 0.011 mmol, 0.16 equiv.) and sodium ascorbate (6 mg, 0.036 mmol, 0.52 equiv.) were added to a mixture of $CHCl_3$ (1.9 mL) and dest. H_2O (2.8 mL). Compound **31** (21 mg, 0.076 mmol, 1.1 equiv.) was added and the reaction mixture was stirred at room temperature overnight. DCM (20 mL) and sat. NH_4Cl solution (20 mL) were added and the phases separated. The aqueous phase was extracted with 2x 20 mL DCM. The combined organic phases were washed with 2x 40 mL 0.1 M EDTA-Na_4 solution and the solvent was removed under reduced pressure. The crude product was subjected

to flash column chromatography (DCM/MeOH : 100/0 → 80/20) to yield compound **9** as a white solid (27 mg, 0.033 mmol, 48 %).

^{1}H-NMR (400 MHz, CD_3OD): *δ* (ppm) = 7.84 (s, 1H), 7.37 (s, 3H), 4.60 (s, 4H), 4.47 (s, 2H), 4.36 (t, *J* = 6.9 Hz, 2H), 2.80 (t, *J* = 7.6 Hz, 2H), 2.71 (t, *J* = 7.0 Hz, 2H), 2.61–2.45 (m, 4H), 2.61–2.45 (m, 4H), 2.30–2.18 (m, 2H), 2.13–2.04 (m, 1H), 1.96–0.98 (m, 33H), 0.96 (d, *J* = 6.5 Hz, 4H), 0.90 (dd, *J* = 6.6, 1.5 Hz, 7H), 0.83 (s, 3H), 0.74 (s, 3H).

^{13}C{^{1}H}-NMR (101 MHz, CD_3OD): *δ* (ppm) = 173.7, 147.1, 146.5, 143.8, 130.5, 128.0, 127.3, 127.0, 124.2, 122.9, 83.8, 76.1, 74.7, 66.5, 64.4, 57.7, 57.6, 54.8, 51.0, 43.6, 43.3, 41.2, 40.7, 37.8, 37.4, 37.1, 36.9, 35.9, 35.8, 35.3, 32.7, 30.6, 29.8, 29.3, 29.1, 27.8, 26.8, 26.6, 26.1, 25.3, 25.0, 23.2, 23.0, 22.3, 19.2, 16.8, 12.4, 12.1.

ESI-MS: *m/z* calculated $[C_{51}H_{72}N_6O_3H]^+$: 817.57387, found: 817.57338.

1.3 NMR spectra

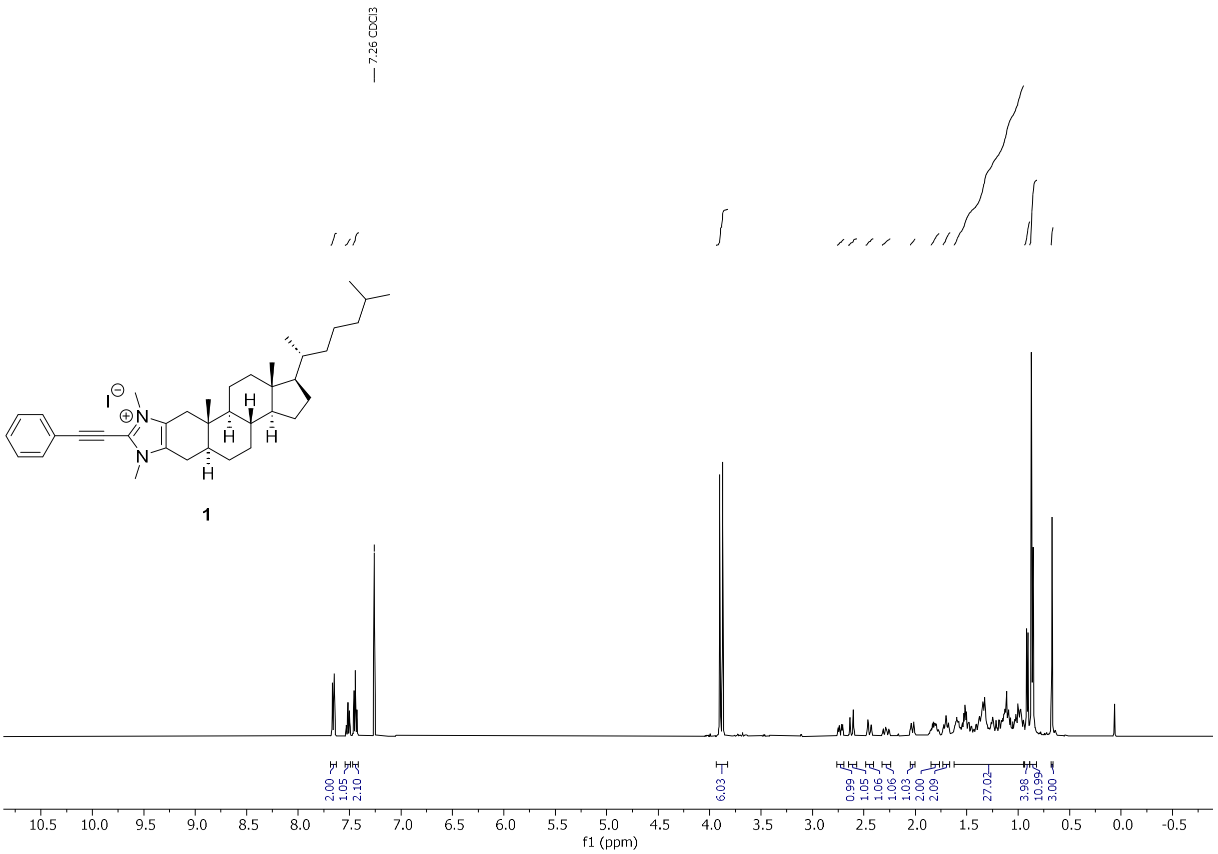

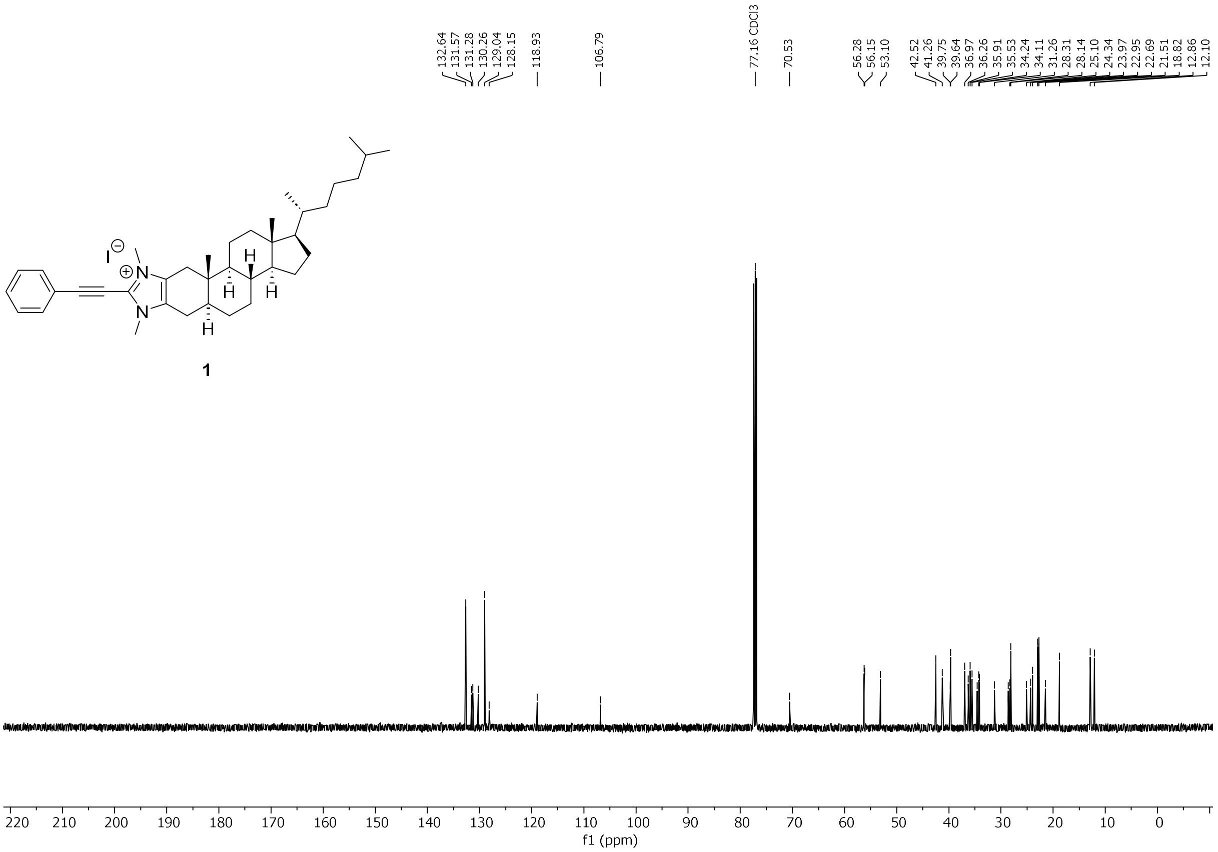

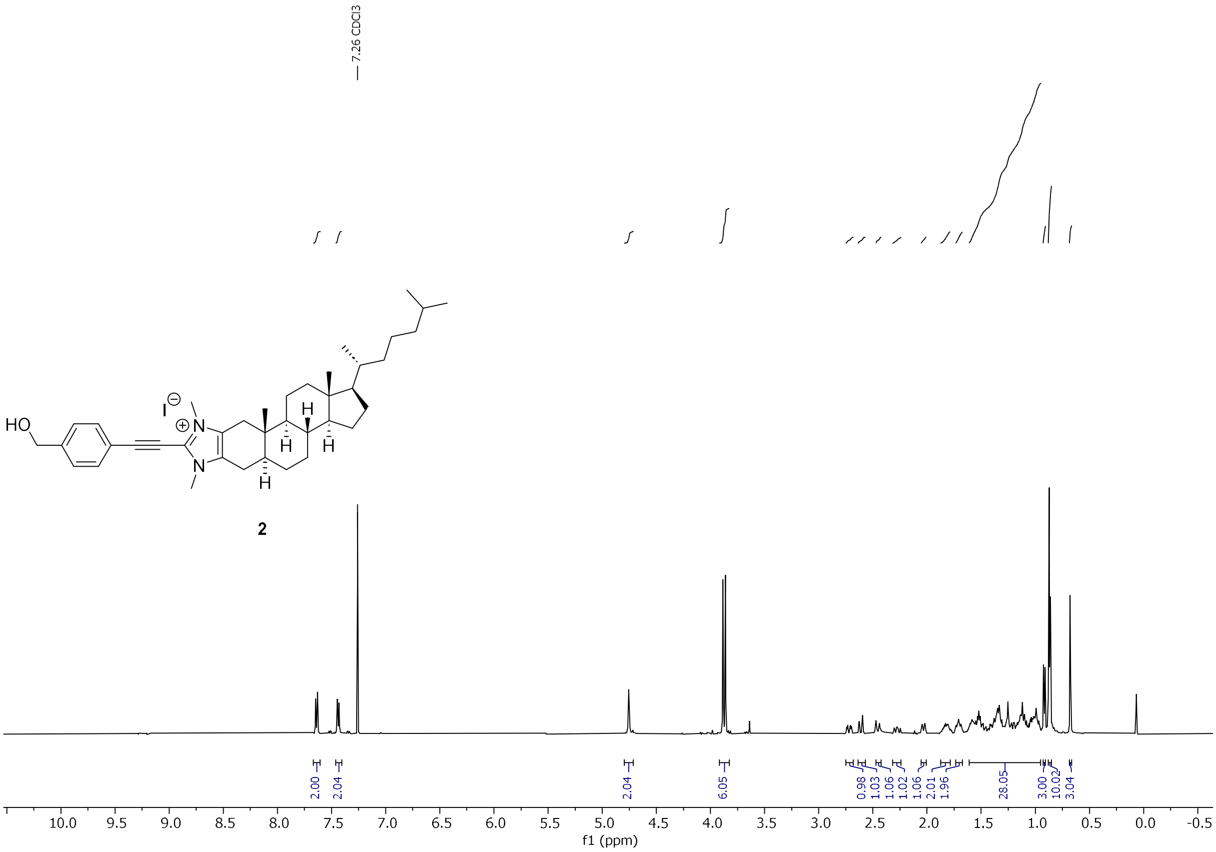
7.26 CDCl3
HO
I
N
N
H
H
H
H
2.00
2.04
2.04
6.05
0.98
1.03
1.06
1.02
1.06
2.01
1.96
28.05
3.00
10.02
3.04
10.0 9.5 9.0 8.5 8.0 7.5 7.0 6.5 6.0 5.5 5.0 4.5 4.0 3.5 3.0 2.5 2.0 1.5 1.0 0.5 0.0 -0.5
f1 (ppm)

2

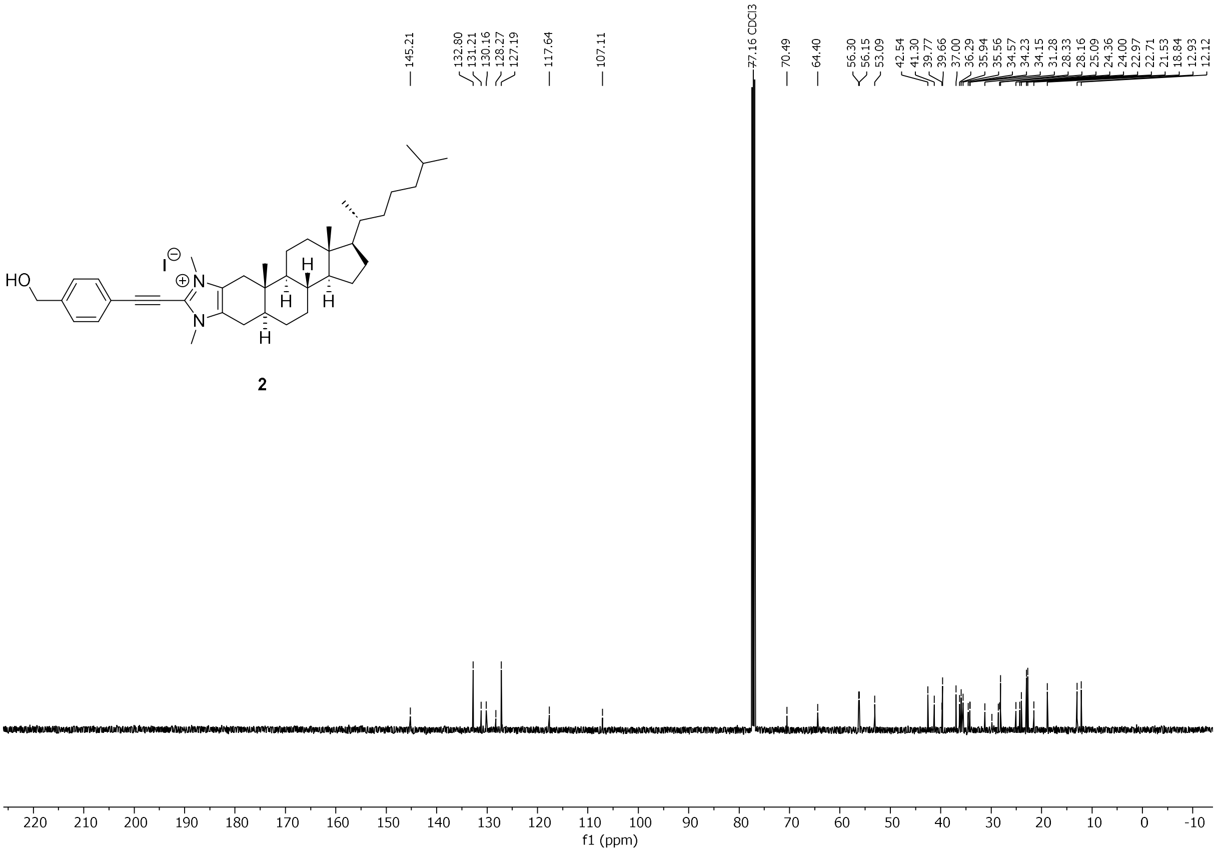
145.21
132.80
131.21
130.16
128.27
127.19
117.64
107.11
77.16 CDCl3
70.49
64.40
56.30
56.15
53.09
42.54
41.30
39.77
39.66
37.00
36.29
35.94
35.56
34.57
34.23
34.15
31.28
28.33
28.16
25.09
24.36
24.00
22.97
22.71
21.53
18.84
12.93
12.12
HO
I
N
N
H
H
H
H
220 210 200 190 180 170 160 150 140 130 120 110 100 90 80 70 60 50 40 30 20 10 0 -10
f1 (ppm)

2

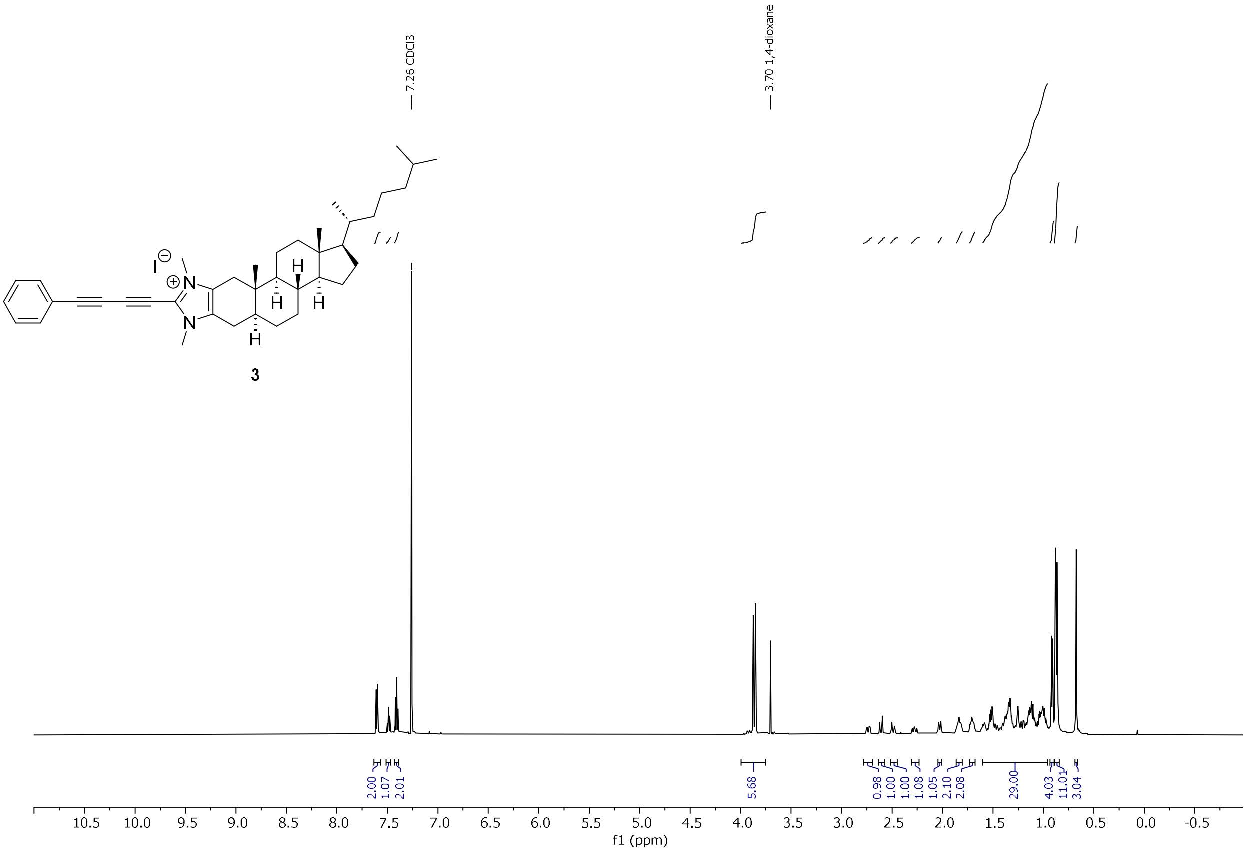
7.26 CDCl3
3.70 1,4-dioxane
3
2.00
1.07
2.01
5.68
0.98
1.00
1.00
1.08
1.05
2.10
2.08
29.00
4.03
11.01
3.04
10.5 10.0 9.5 9.0 8.5 8.0 7.5 7.0 6.5 6.0 5.5 5.0 4.5 4.0 3.5 3.0 2.5 2.0 1.5 1.0 0.5 0.0 -0.5
f1 (ppm)

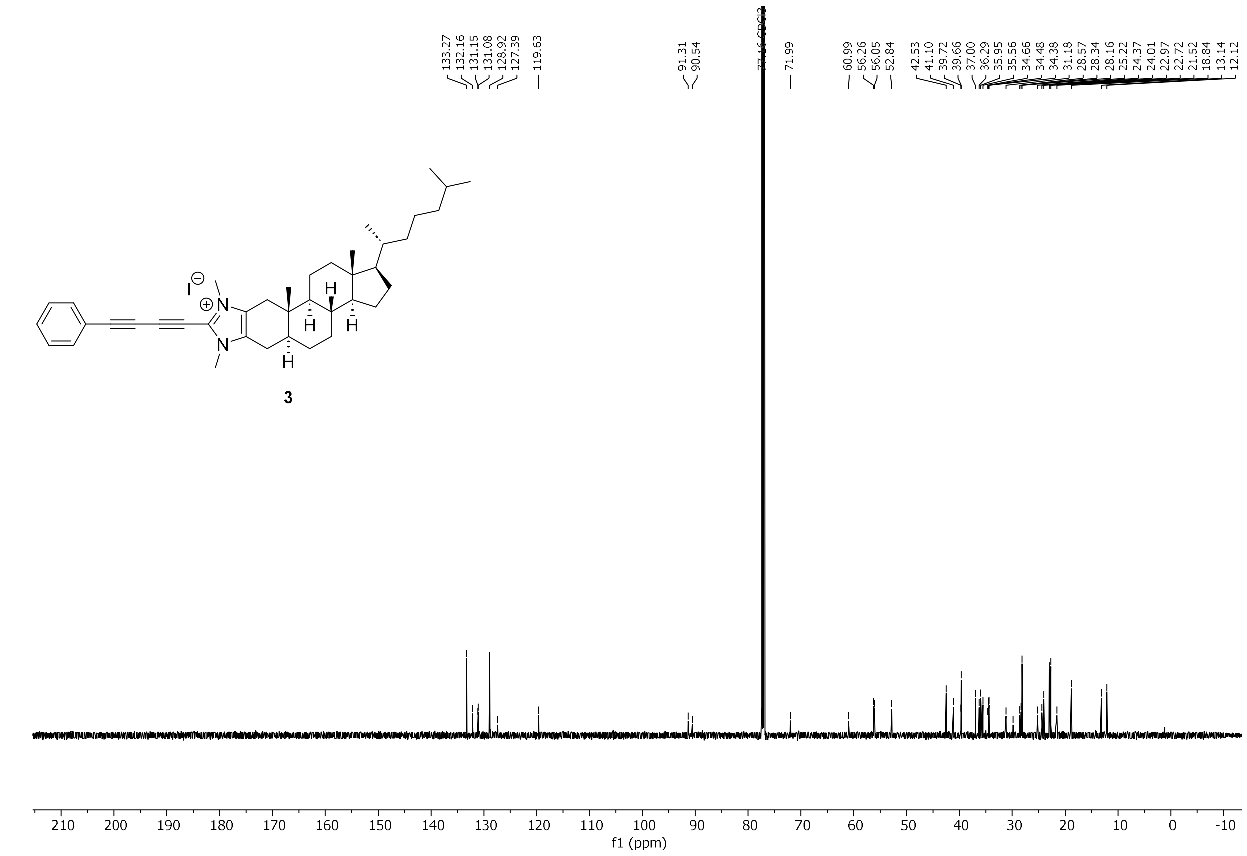
133.27
132.16
131.15
131.08
128.92
127.39
119.63
91.31
90.54
77.16 CDCl3
71.99
60.99
56.26
56.05
52.84
42.53
41.10
39.72
39.66
37.00
36.29
35.95
35.56
34.66
34.48
34.38
31.18
28.57
28.34
28.16
25.22
24.37
24.01
22.97
22.72
21.52
18.84
13.14
12.12
3
210 200 190 180 170 160 150 140 130 120 110 100 90 80 70 60 50 40 30 20 10 0 -10
f1 (ppm)

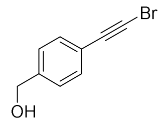
Br
OH

17

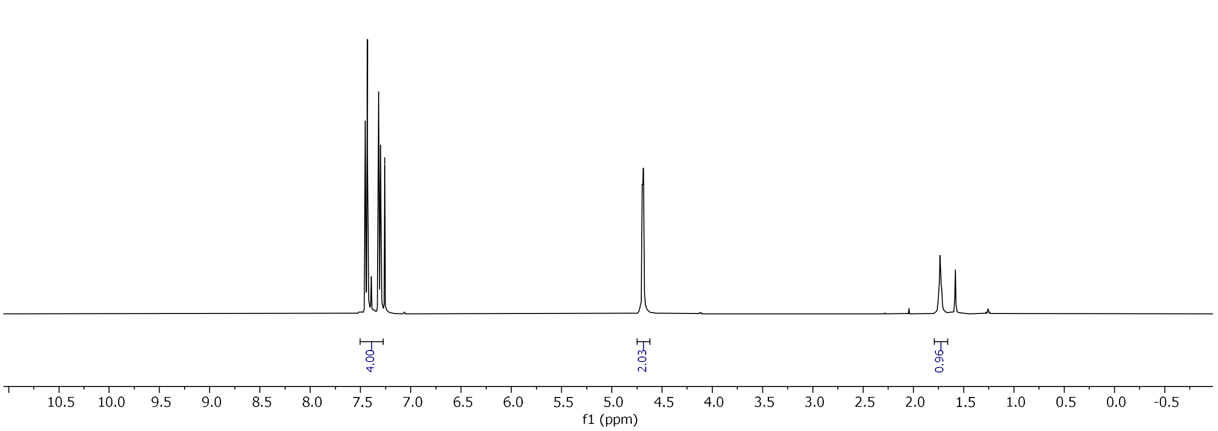
7.26 CDCl3
4.00
2.03
0.96
f1 (ppm)

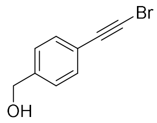
Br
OH

17

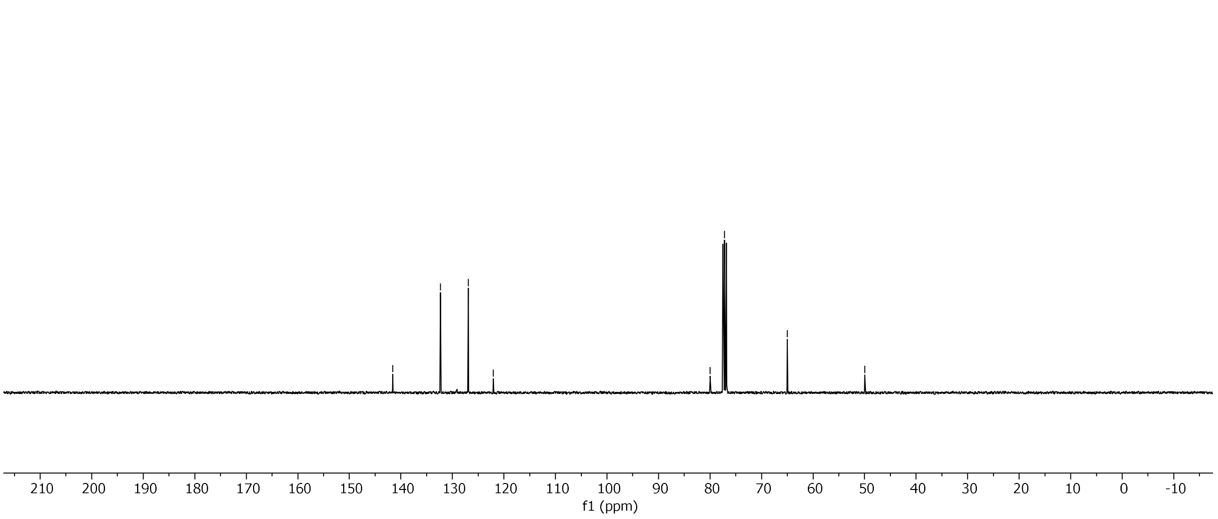
141.59
132.33
126.90
122.05
79.99
77.16 CDCl3
65.02
49.94
f1 (ppm)

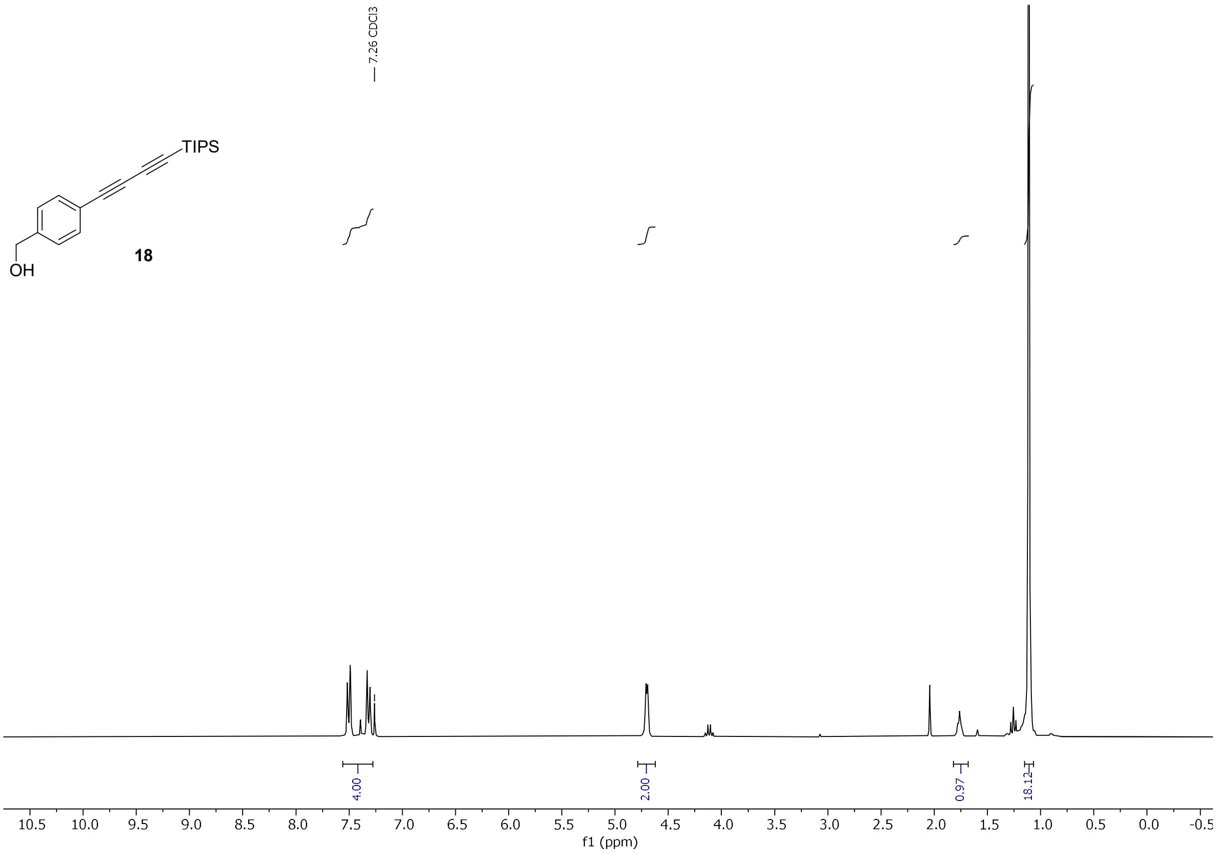
TIPS
OH
18
7.26 CDCl3
4.00
2.00
0.97
18.12
10.5 10.0 9.5 9.0 8.5 8.0 7.5 7.0 6.5 6.0 5.5 5.0 4.5 4.0 3.5 3.0 2.5 2.0 1.5 1.0 0.5 0.0 -0.5
f1 (ppm)

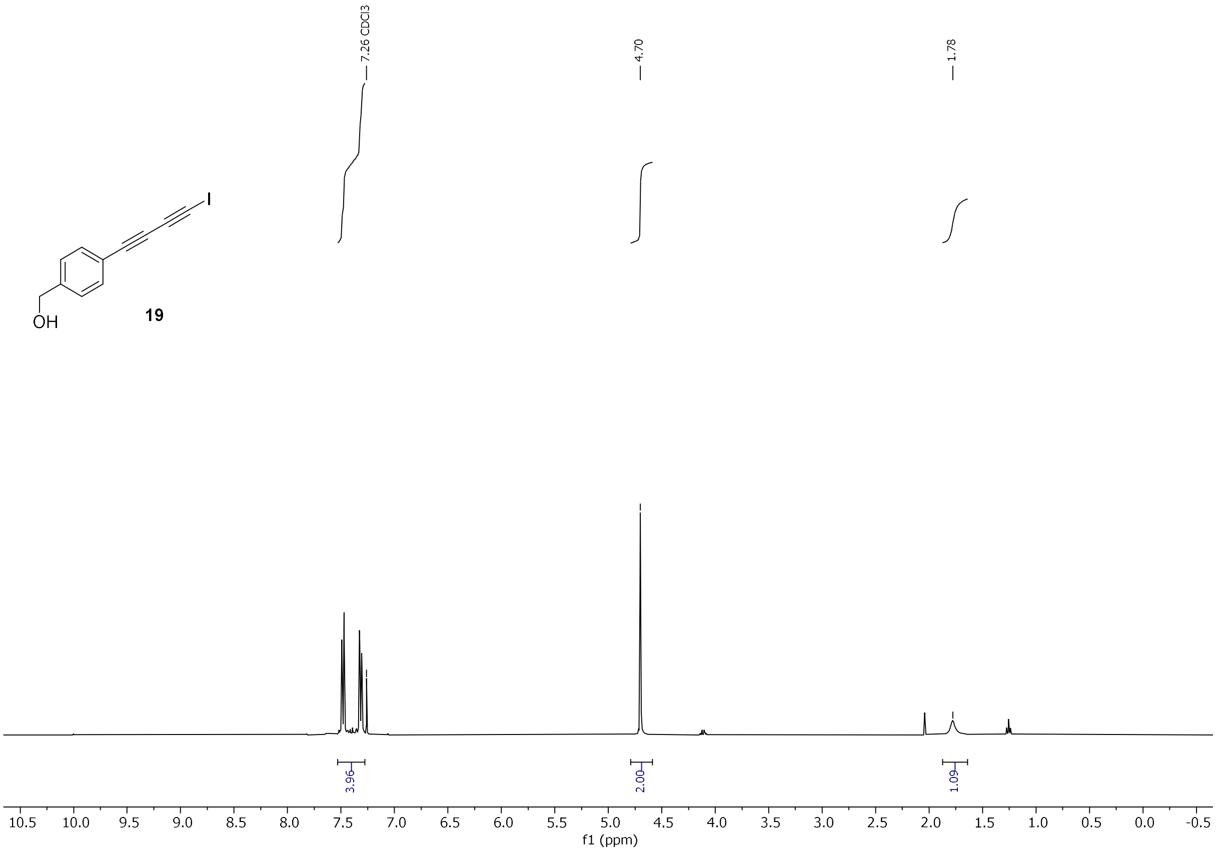
7.26 CDCl3
4.70
1.78
3.96
2.00
1.09
OH
I
19
10.5 10.0 9.5 9.0 8.5 8.0 7.5 7.0 6.5 6.0 5.5 5.0 4.5 4.0 3.5 3.0 2.5 2.0 1.5 1.0 0.5 0.0 -0.5
f1 (ppm)

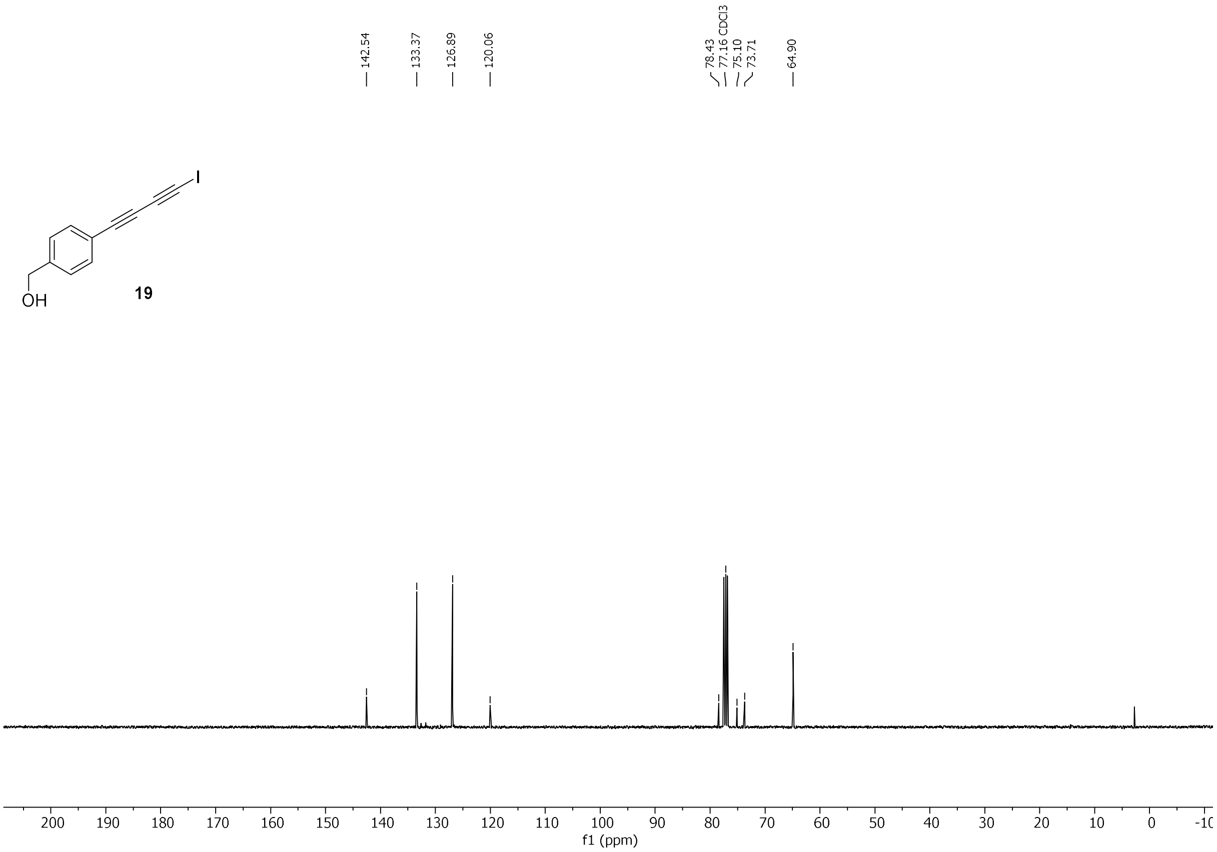
142.54
133.37
126.89
120.06
78.43
77.16 CDCl3
75.10
73.71
64.90
OH
I
19
200 190 180 170 160 150 140 130 120 110 100 90 80 70 60 50 40 30 20 10 0 -10
f1 (ppm)

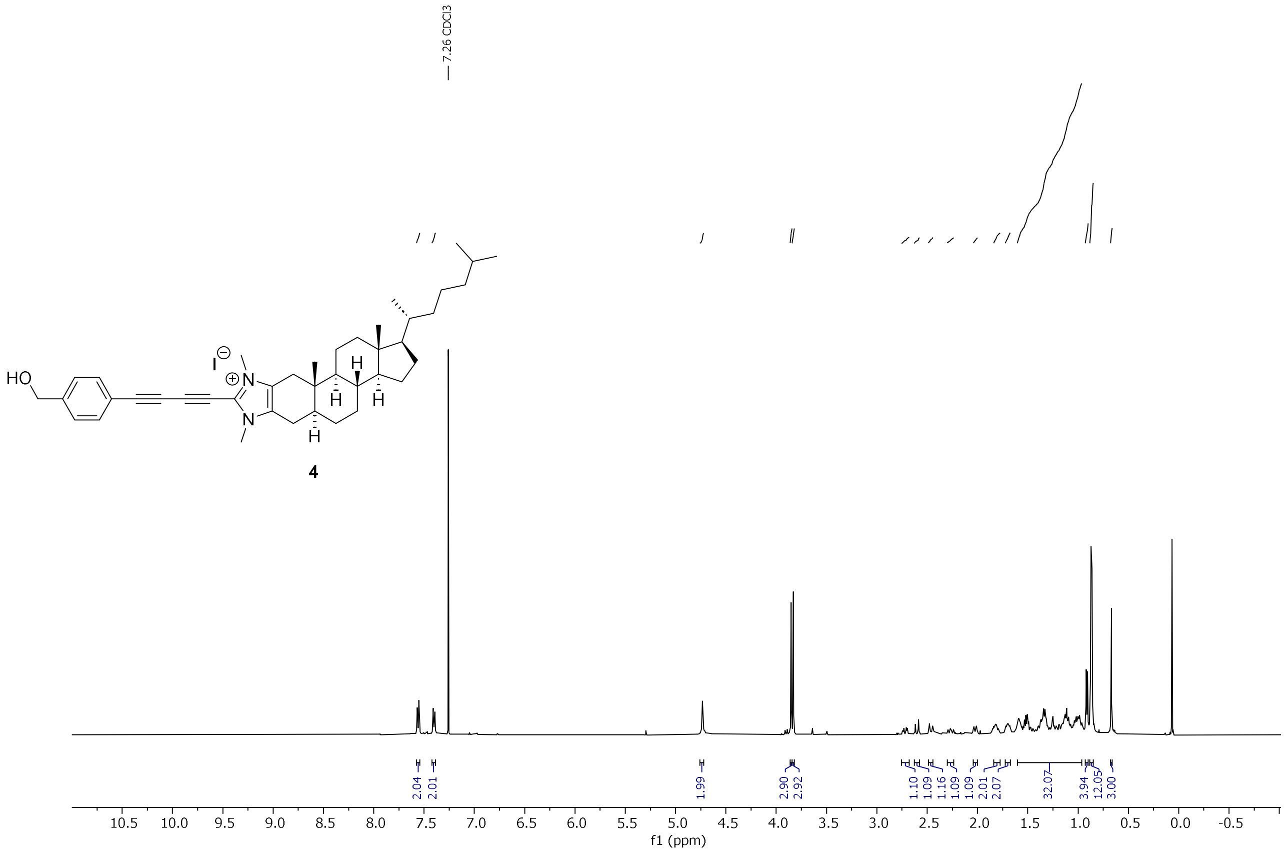
7.26 CDCl3
HO
I
N
H
4
2.04
2.01
1.99
2.90
2.92
1.10
1.09
1.16
1.09
2.01
2.07
32.07
3.94
12.05
3.00
10.5 10.0 9.5 9.0 8.5 8.0 7.5 7.0 6.5 6.0 5.5 5.0 4.5 4.0 3.5 3.0 2.5 2.0 1.5 1.0 0.5 0.0 -0.5
f1 (ppm)

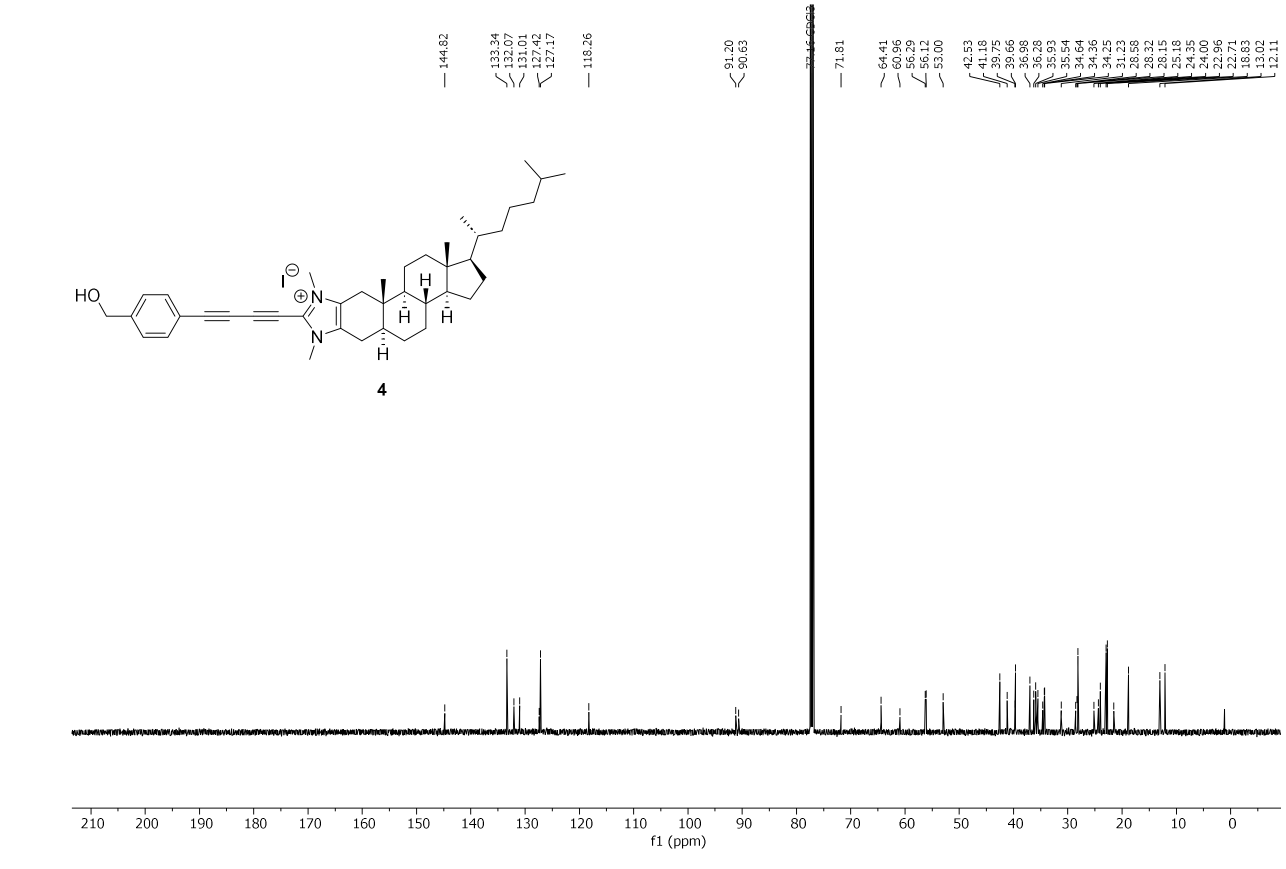
144.82
133.34
132.07
131.01
127.42
127.17
118.26
91.20
90.63
71.81
64.41
60.96
56.29
56.12
53.00
42.53
41.18
39.75
39.66
36.98
36.28
35.93
35.54
34.64
34.36
34.25
31.23
28.58
28.32
28.15
25.18
24.35
24.00
22.96
22.71
18.83
13.02
12.11
HO
I
N
H
4
210 200 190 180 170 160 150 140 130 120 110 100 90 80 70 60 50 40 30 20 10 0
f1 (ppm)

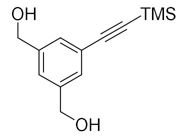
OH
TMS
OH

22

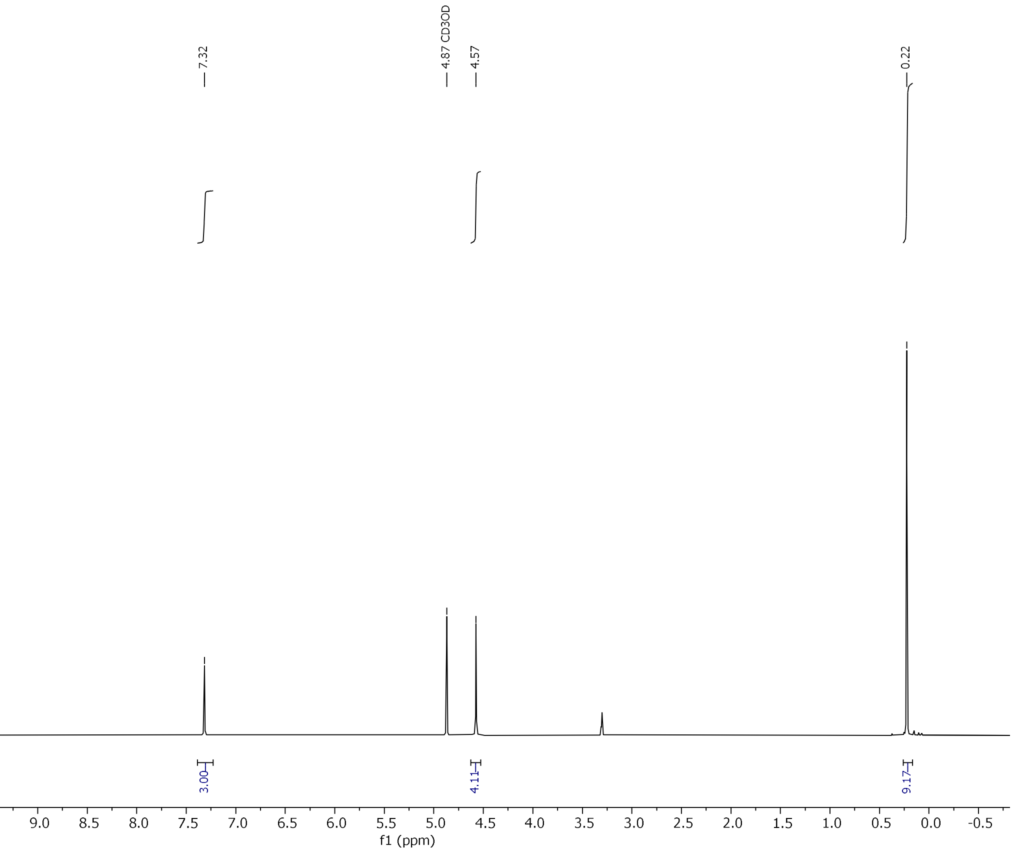
7.32
4.87 CD3OD
4.57
0.22
3.00
4.11
9.17
11.0 10.5 10.0 9.5 9.0 8.5 8.0 7.5 7.0 6.5 6.0 5.5 5.0 4.5 4.0 3.5 3.0 2.5 2.0 1.5 1.0 0.5 0.0 -0.5
f1 (ppm)

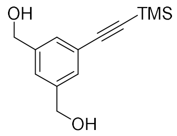
OH
TMS
OH

22

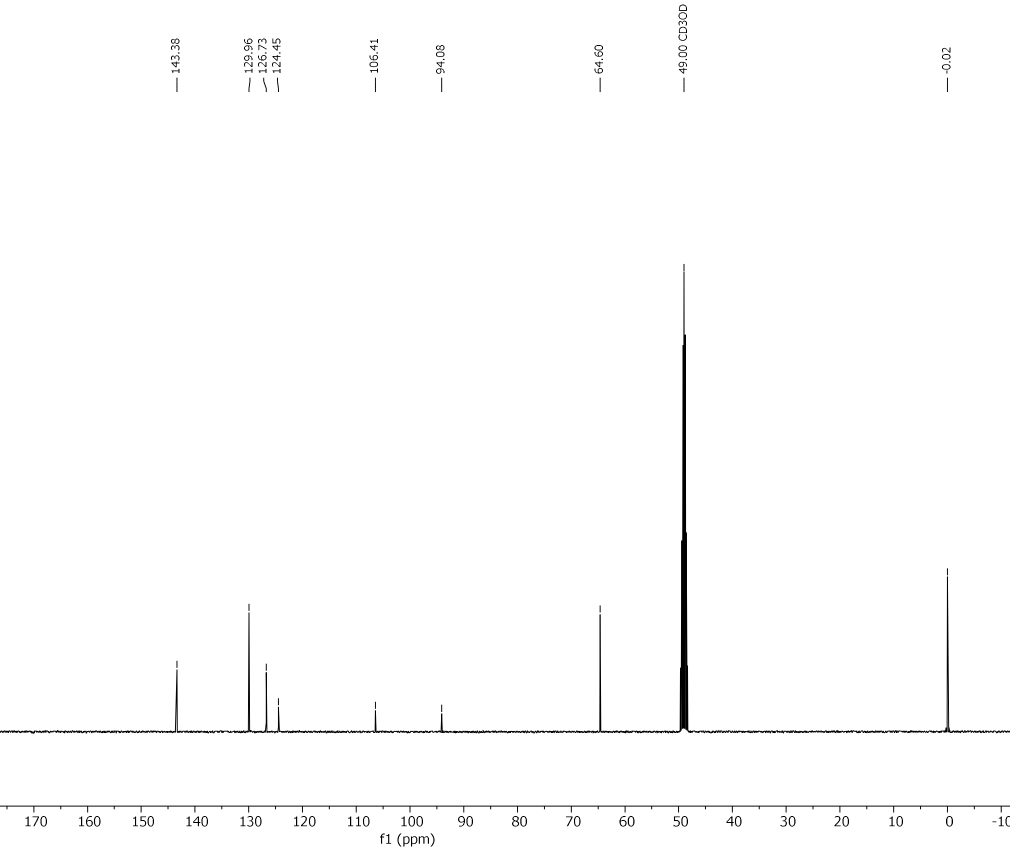
143.38
129.96
126.73
124.45
106.41
94.08
64.60
49.00 CD3OD
-0.02
210 200 190 180 170 160 150 140 130 120 110 100 90 80 70 60 50 40 30 20 10 0
f1 (ppm)

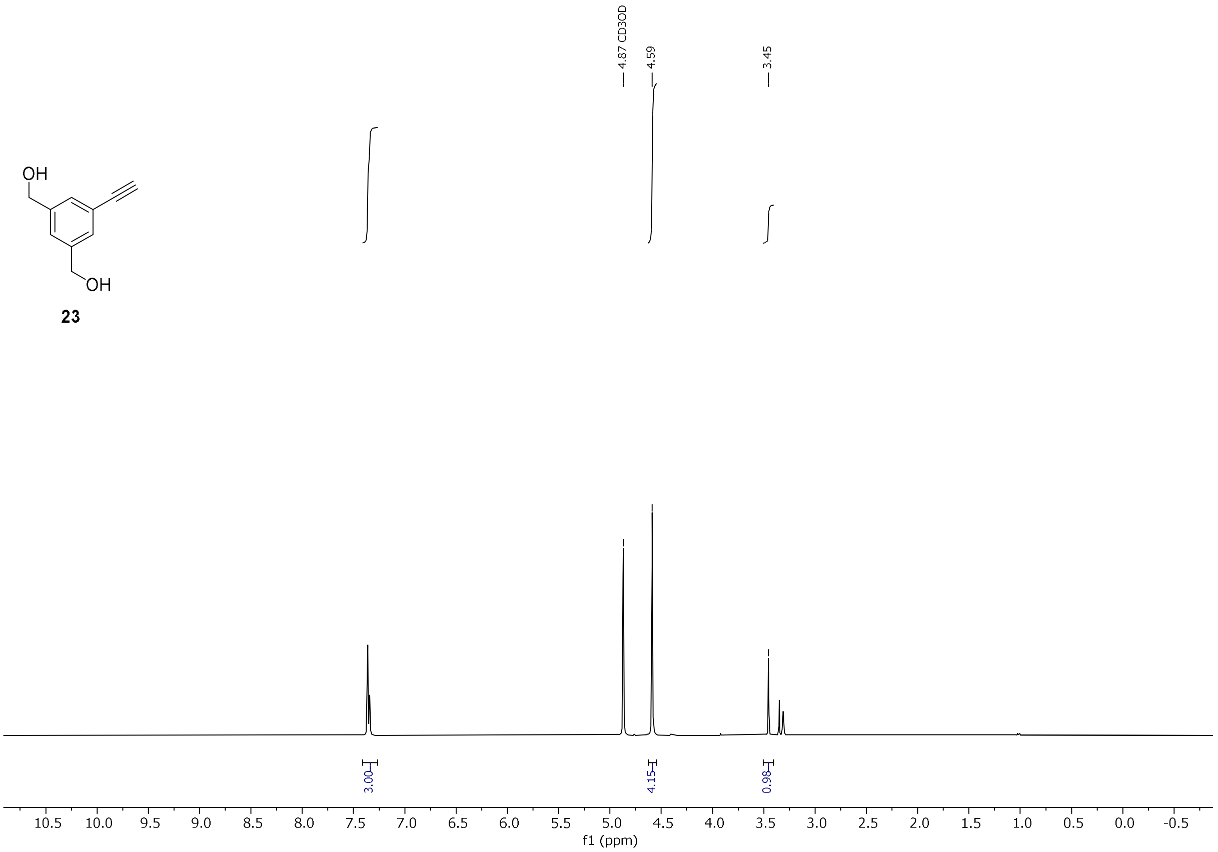

4.87 CD3OD
4.59
3.45
3.00
4.15
0.98
10.5 10.0 9.5 9.0 8.5 8.0 7.5 7.0 6.5 6.0 5.5 5.0 4.5 4.0 3.5 3.0 2.5 2.0 1.5 1.0 0.5 0.0 -0.5
f1 (ppm)

23

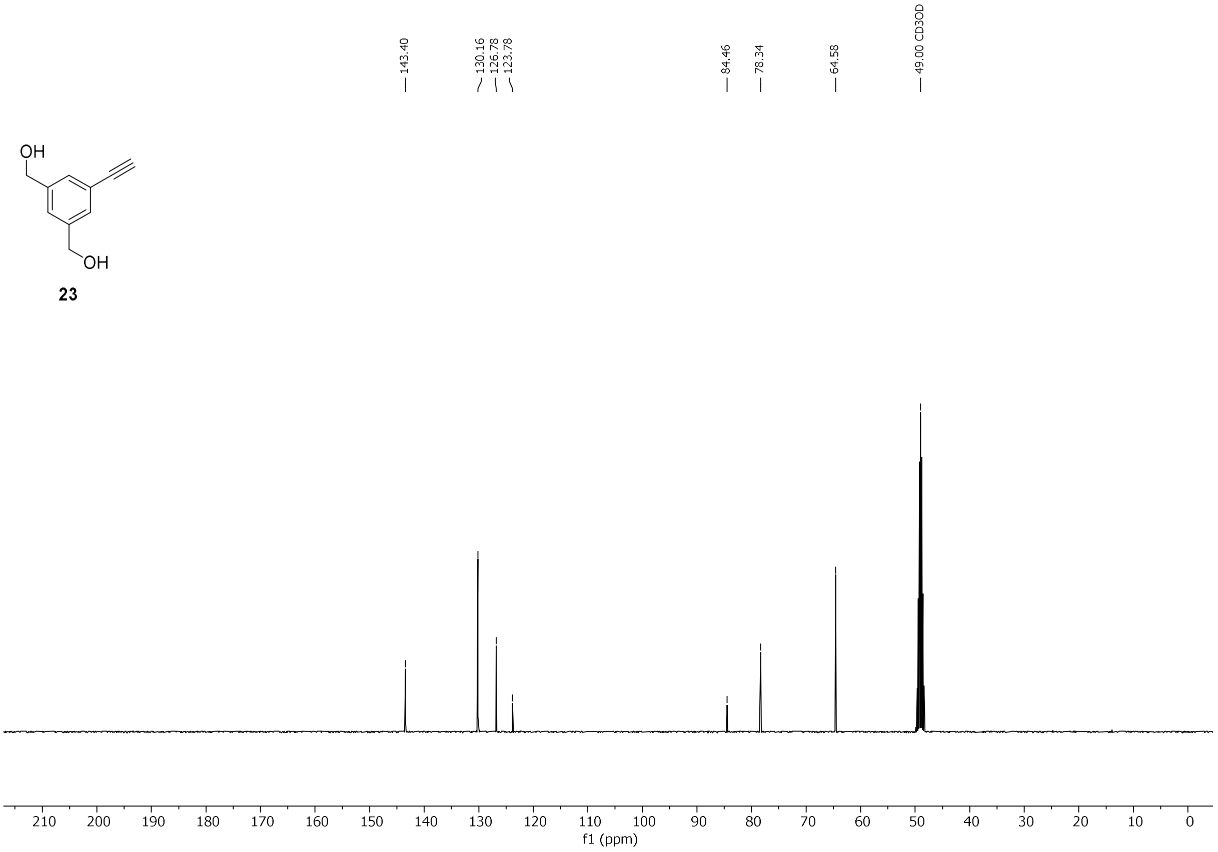

143.40
130.16
126.78
123.78
84.46
78.34
64.58
49.00 CD3OD
210 200 190 180 170 160 150 140 130 120 110 100 90 80 70 60 50 40 30 20 10 0
f1 (ppm)

23

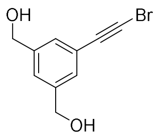

OH
Br
OH

24

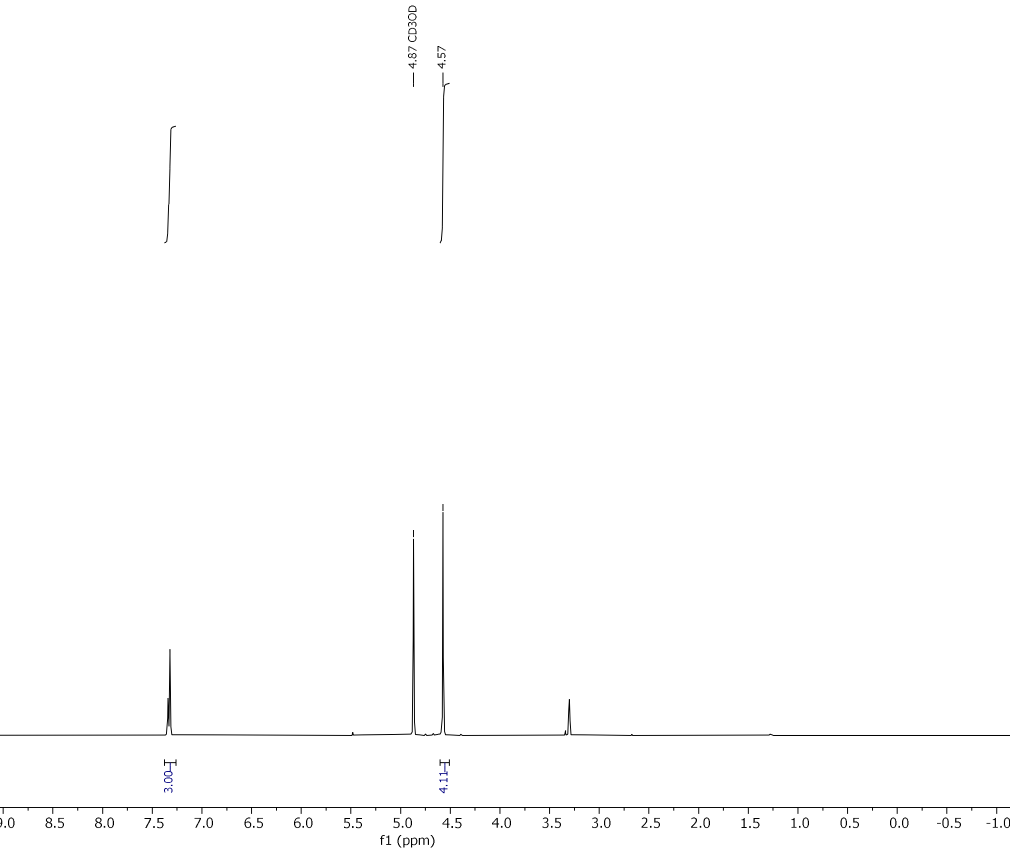

4.87 CD3OD
4.57
3.00
4.11
10.5 10.0 9.5 9.0 8.5 8.0 7.5 7.0 6.5 6.0 5.5 5.0 4.5 4.0 3.5 3.0 2.5 2.0 1.5 1.0 0.5 0.0 -0.5 -1.0
f1 (ppm)

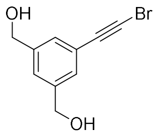

OH
Br
OH

24

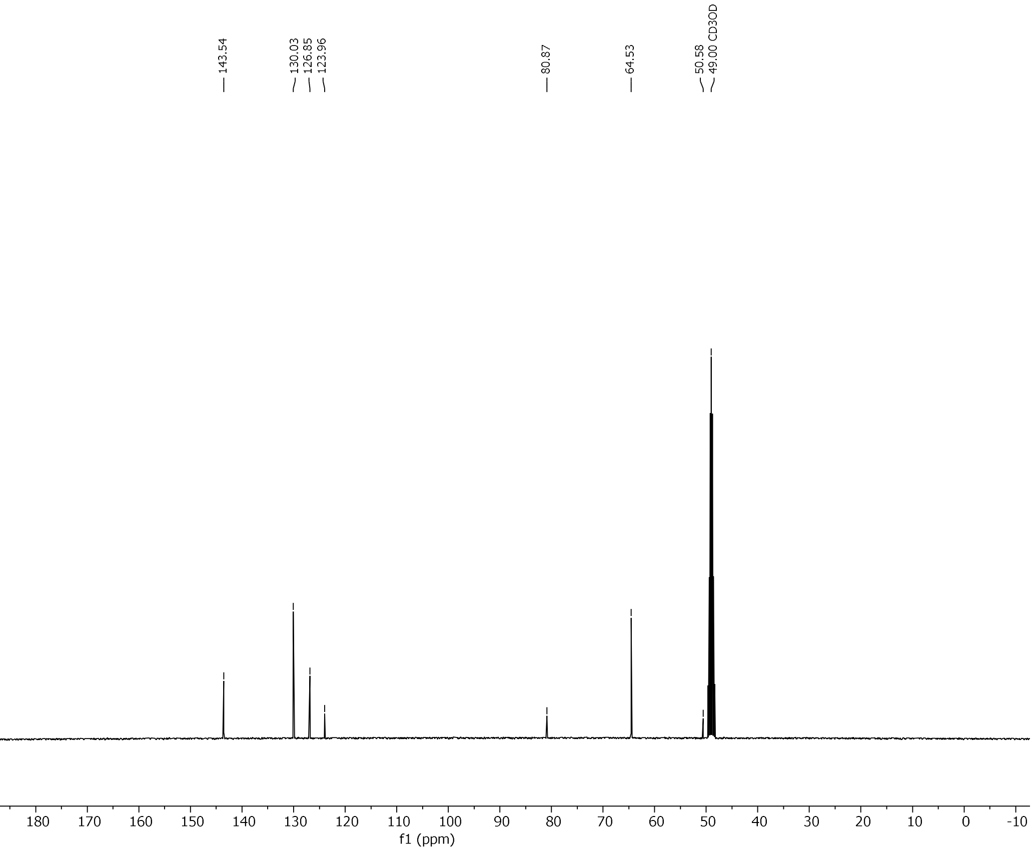

143.54
130.03
126.85
123.96
80.87
64.53
50.58
49.00 CD3OD
220 210 200 190 180 170 160 150 140 130 120 110 100 90 80 70 60 50 40 30 20 10 0 -10
f1 (ppm)

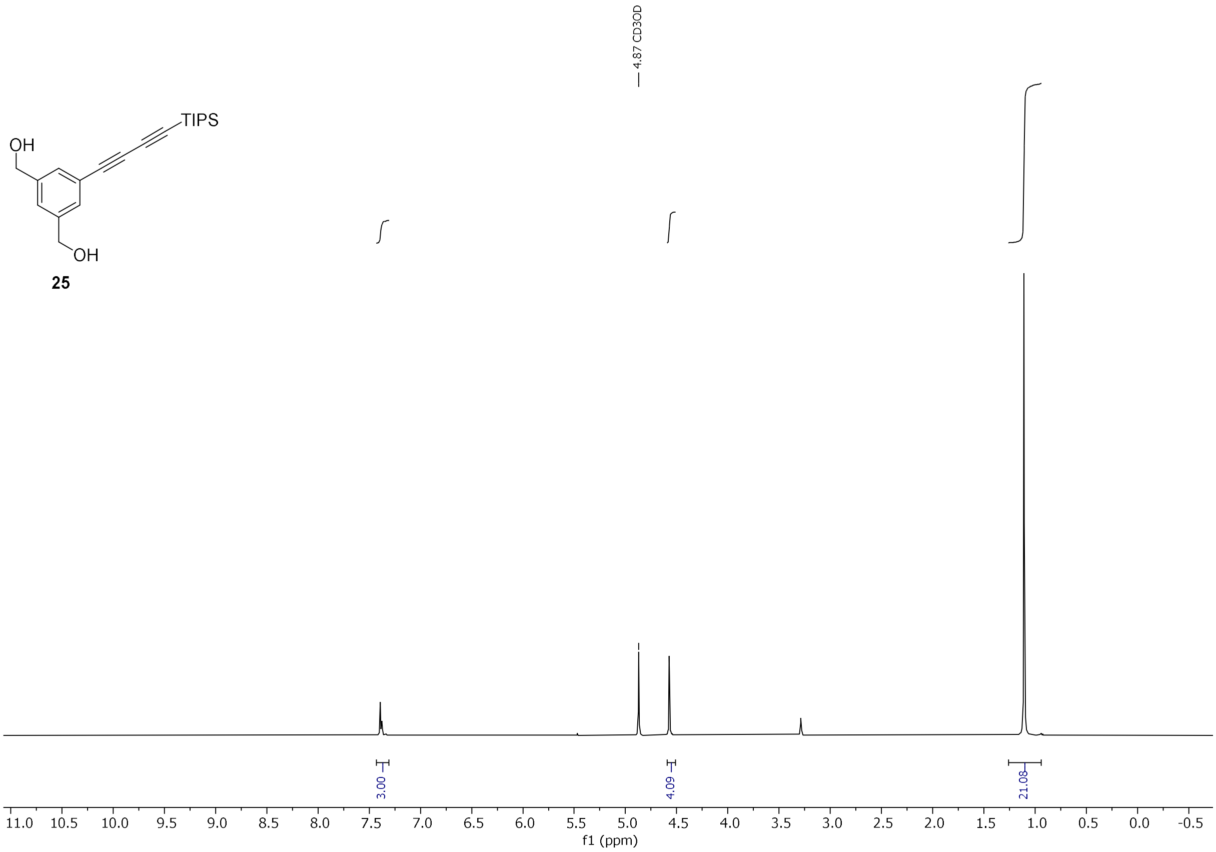

4.87 CD3OD
TIPS
OH
OH
25
3.00
4.09
21.08
11.0 10.5 10.0 9.5 9.0 8.5 8.0 7.5 7.0 6.5 6.0 5.5 5.0 4.5 4.0 3.5 3.0 2.5 2.0 1.5 1.0 0.5 0.0 -0.5
f1 (ppm)

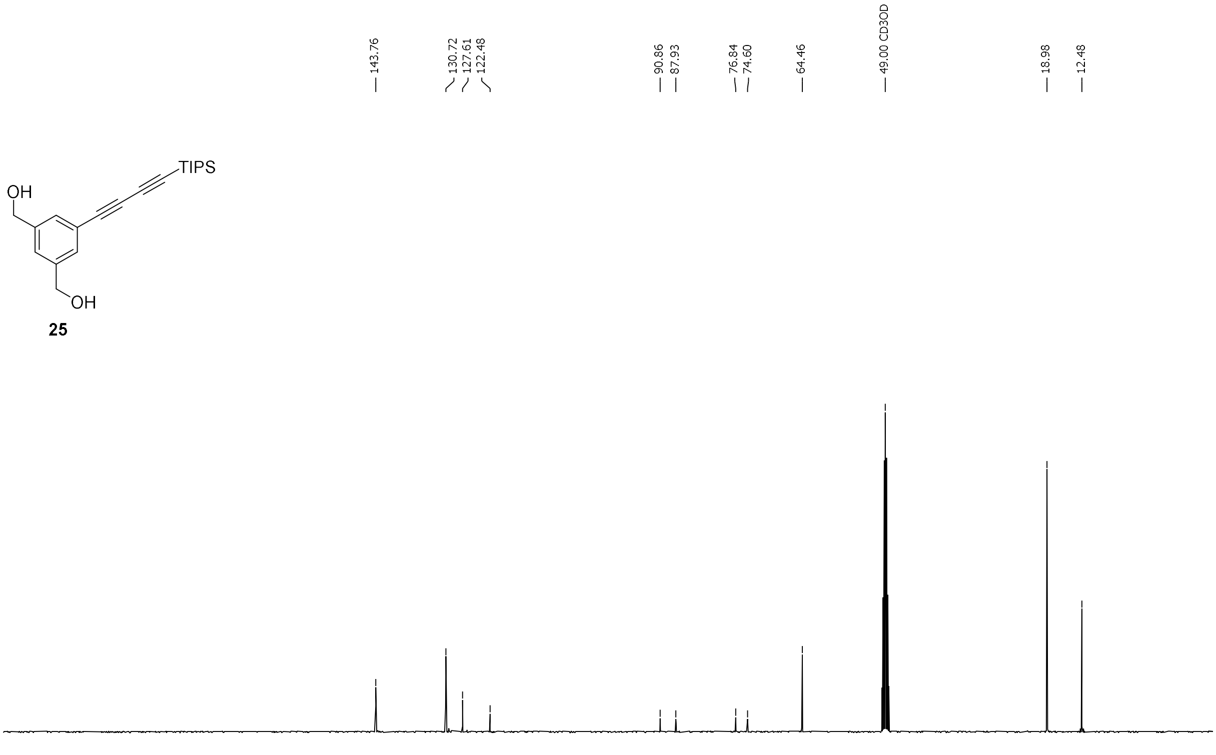

143.76
130.72
127.61
122.48
90.86
87.93
76.84
74.60
64.46
49.00 CD3OD
18.98
12.48
TIPS
OH
OH
25
210 200 190 180 170 160 150 140 130 120 110 100 90 80 70 60 50 40 30 20 10 0 -10
f1 (ppm)

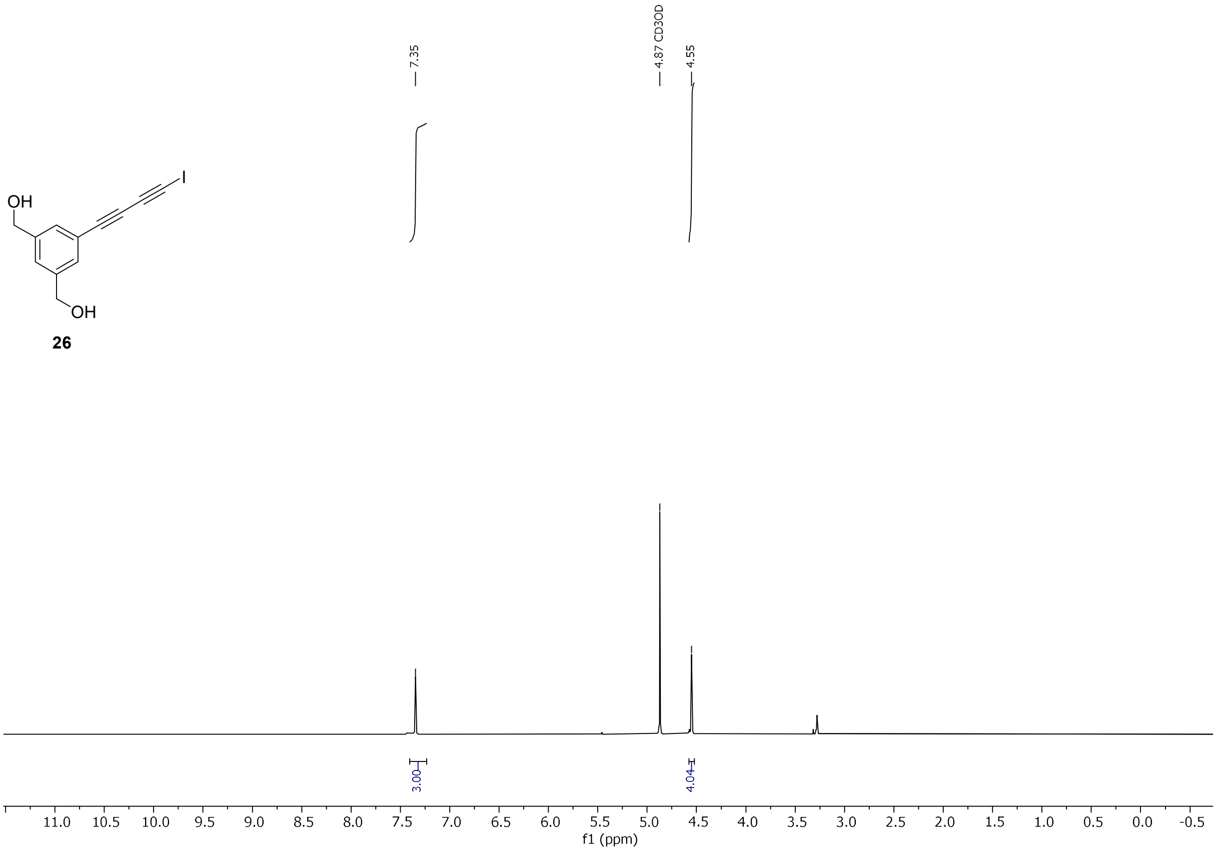
7.35
4.87 CD3OD
4.55
OH
I
OH
26
3.00
4.04
f1 (ppm)

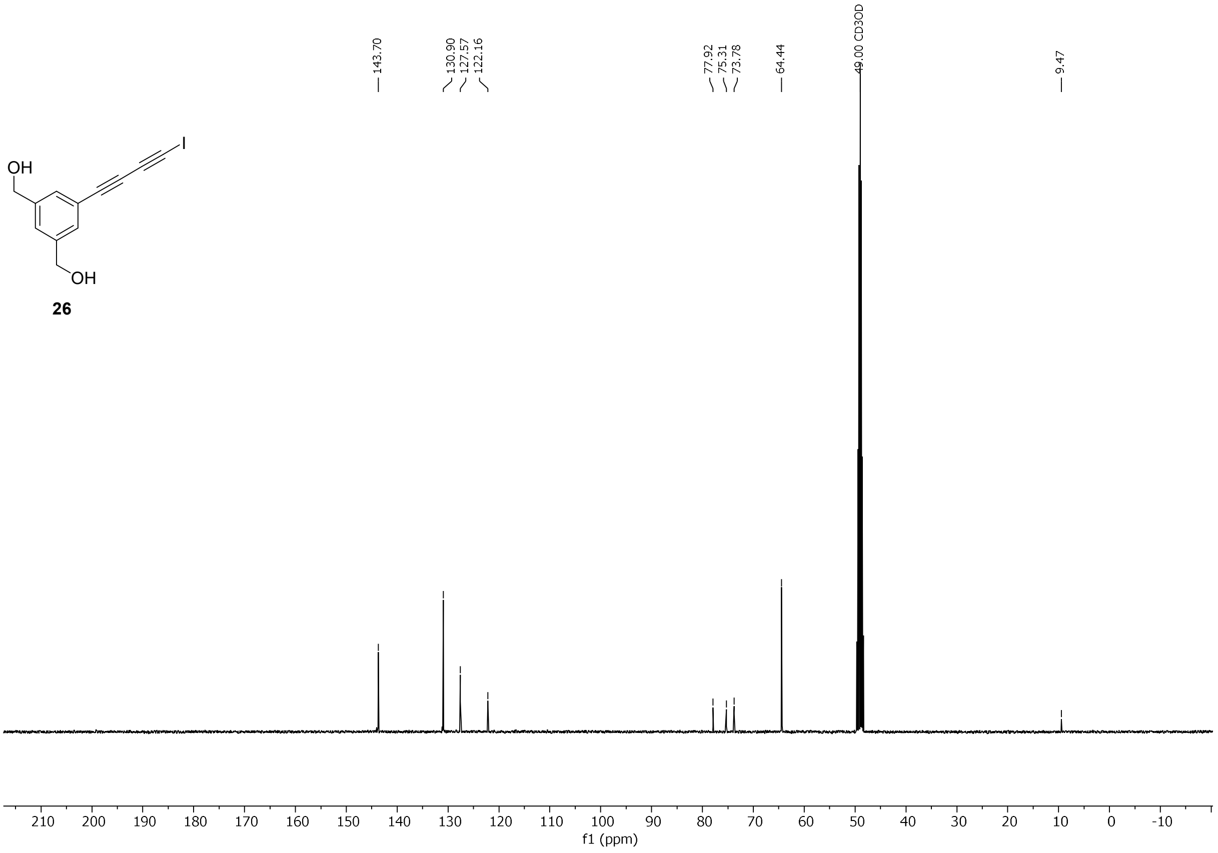
143.70
130.90
127.57
122.16
77.92
75.31
73.78
64.44
49.00 CD3OD
9.47
OH
I
OH
26
f1 (ppm)

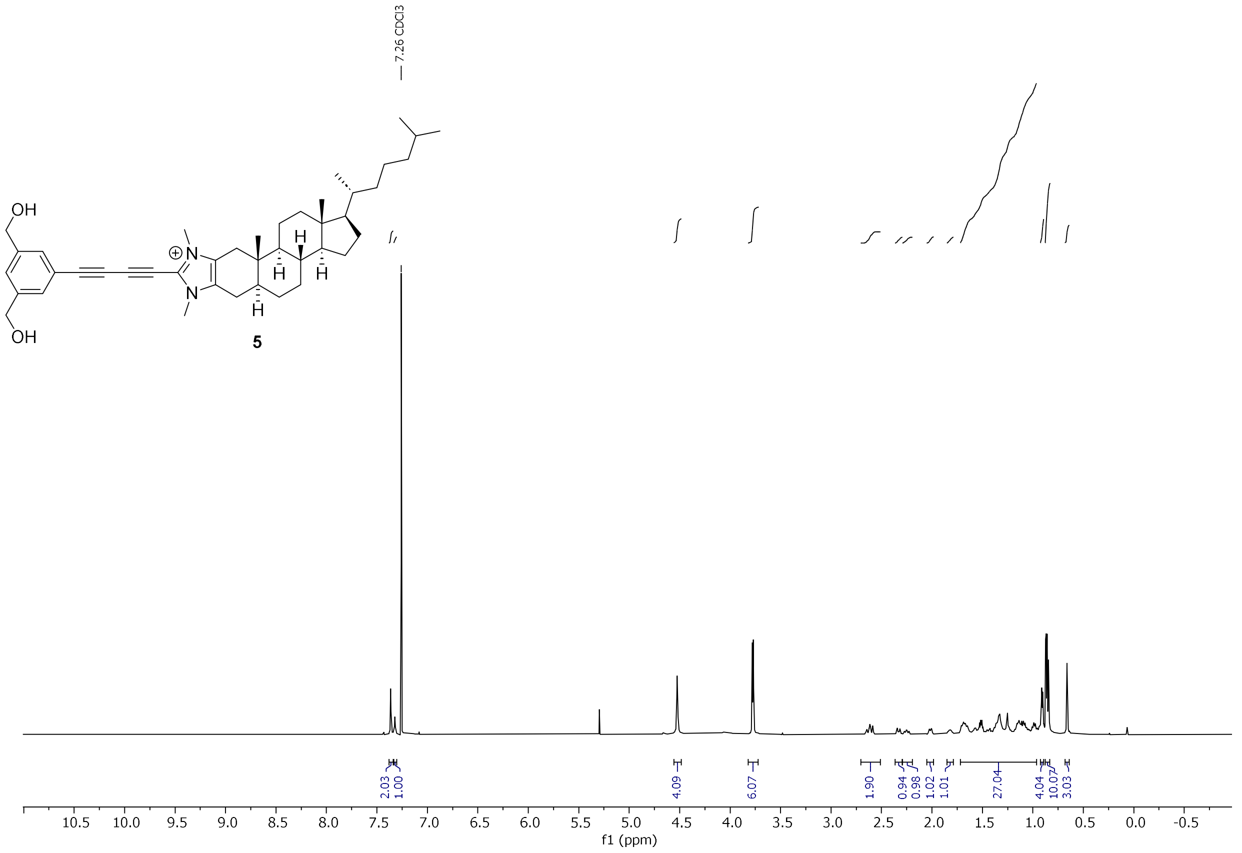
7.26 CDCl3
OH
OH
N
N
H
H
H
H
5
2.03
1.00
4.09
6.07
1.90
0.94
0.98
1.02
1.01
27.04
4.04
10.07
3.03
10.5 10.0 9.5 9.0 8.5 8.0 7.5 7.0 6.5 6.0 5.5 5.0 4.5 4.0 3.5 3.0 2.5 2.0 1.5 1.0 0.5 0.0 -0.5
f1 (ppm)

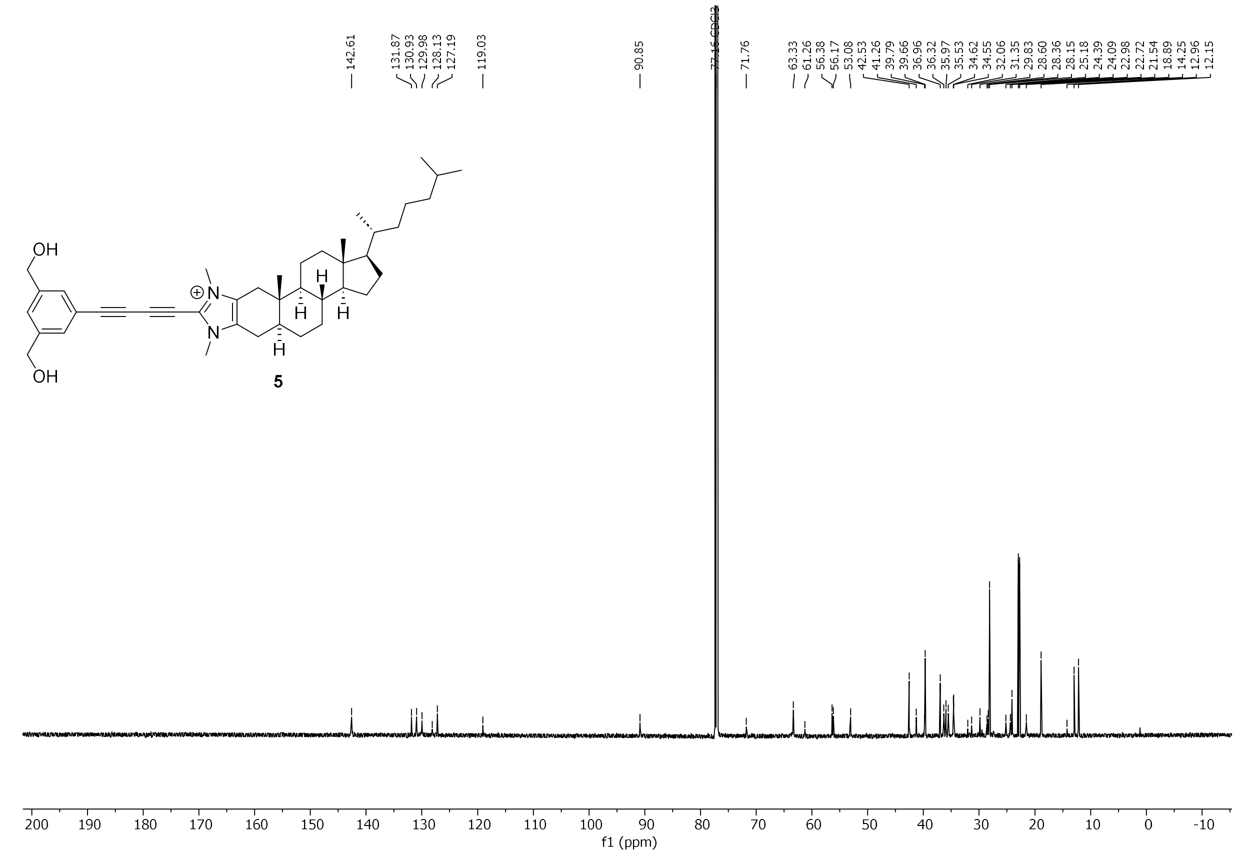
142.61
131.87
130.93
129.98
128.13
127.19
119.03
90.85
77.16 CDCl3
71.76
63.33
61.26
56.38
56.17
53.08
42.53
41.26
39.79
39.66
36.96
36.32
35.97
35.53
34.62
34.55
32.06
31.35
29.83
28.60
28.36
28.15
25.18
24.39
24.09
22.98
22.72
21.54
18.89
14.25
12.96
12.15
OH
OH
N
N
H
H
H
H
5
200 190 180 170 160 150 140 130 120 110 100 90 80 70 60 50 40 30 20 10 0 -10
f1 (ppm)

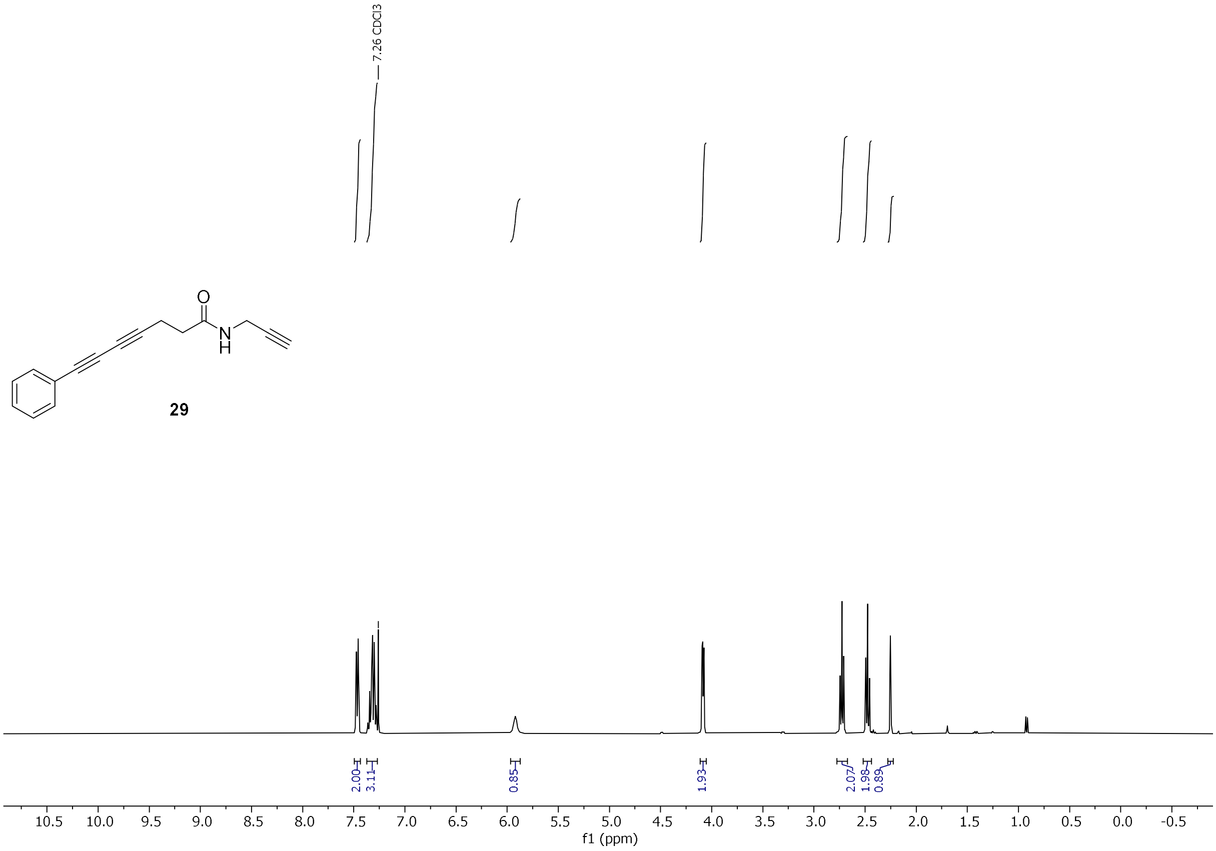
7.26 CDCl3
29
2.00
3.11
0.85
1.93
2.07
1.98
0.89
f1 (ppm)

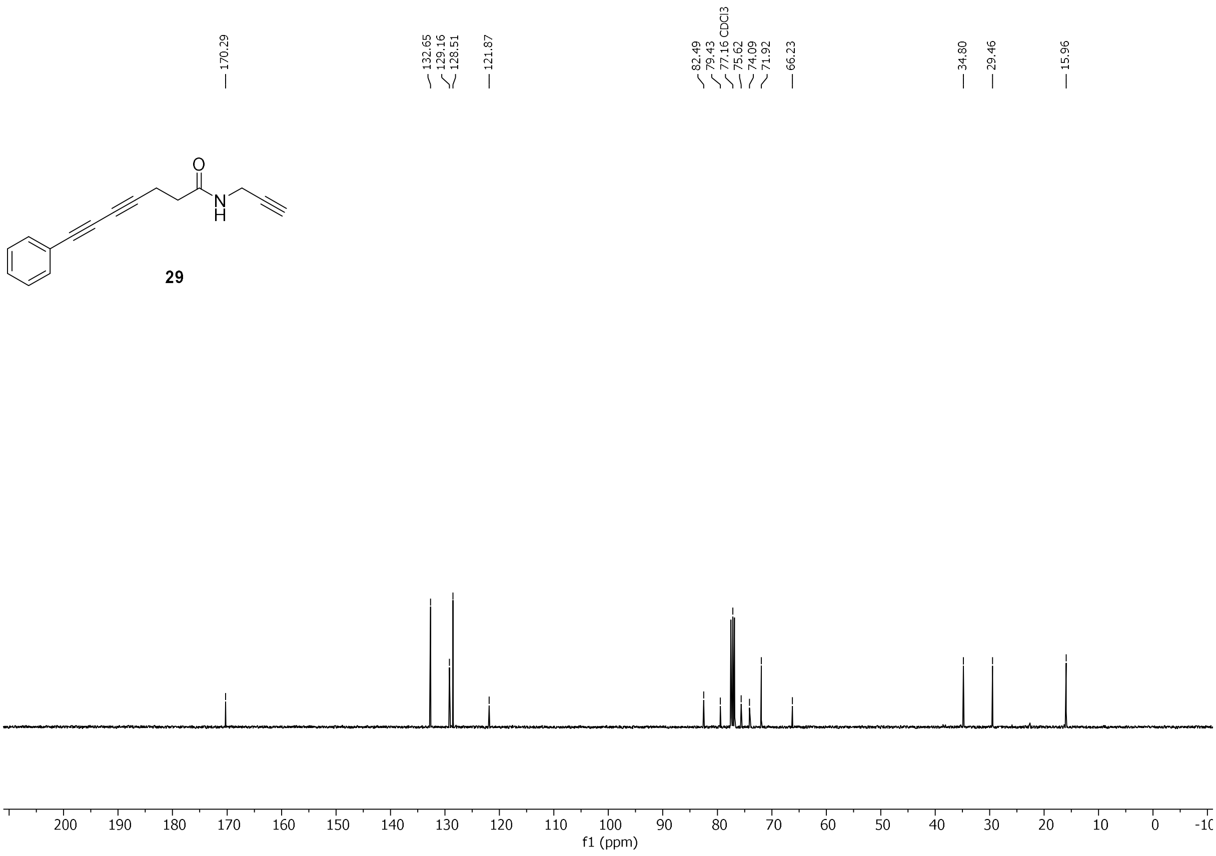
170.29
132.65
129.16
128.51
121.87
82.49
79.43
77.16 CDCl3
75.62
74.09
71.92
66.23
34.80
29.46
15.96
29
f1 (ppm)

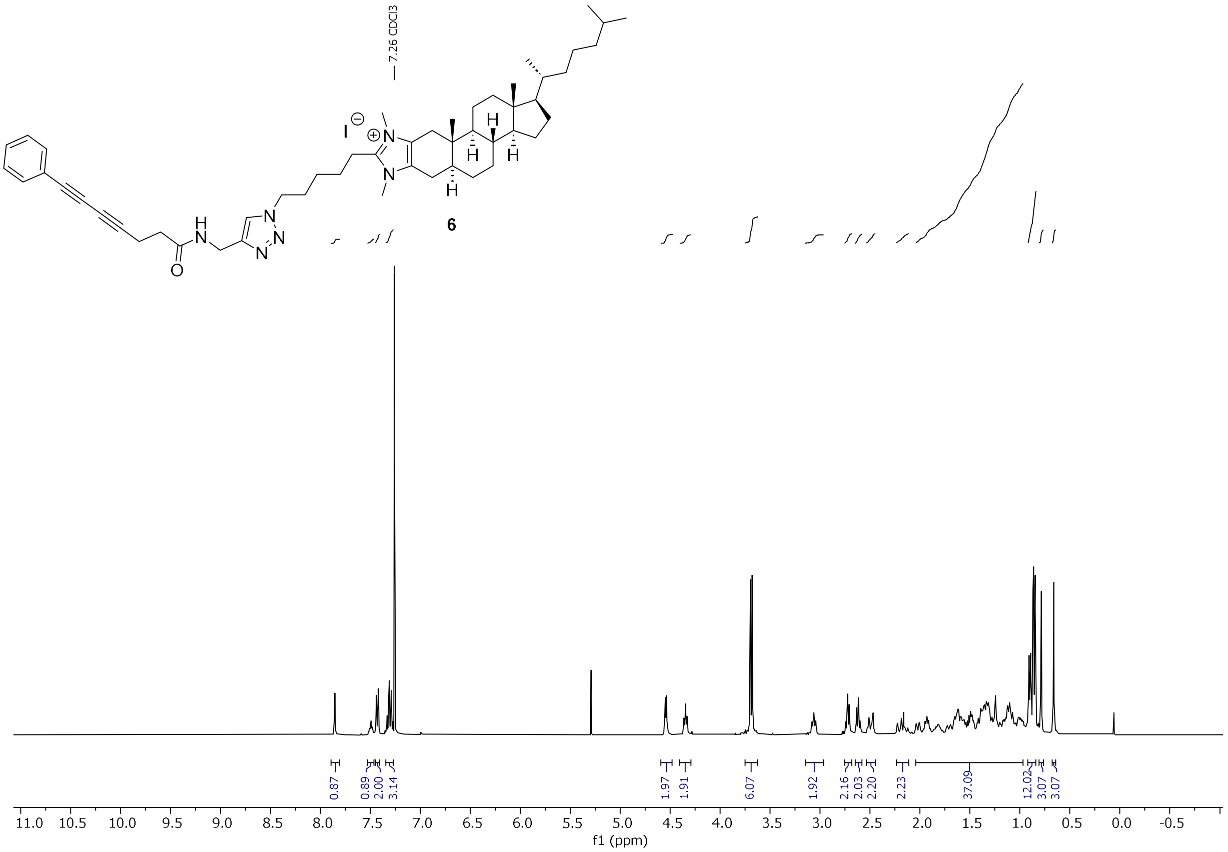
7.26 CDCl3
6
0.87
0.89
2.00
3.14
1.97
1.91
6.07
1.92
2.16
2.03
2.20
2.23
37.09
12.02
3.07
3.07
f1 (ppm)

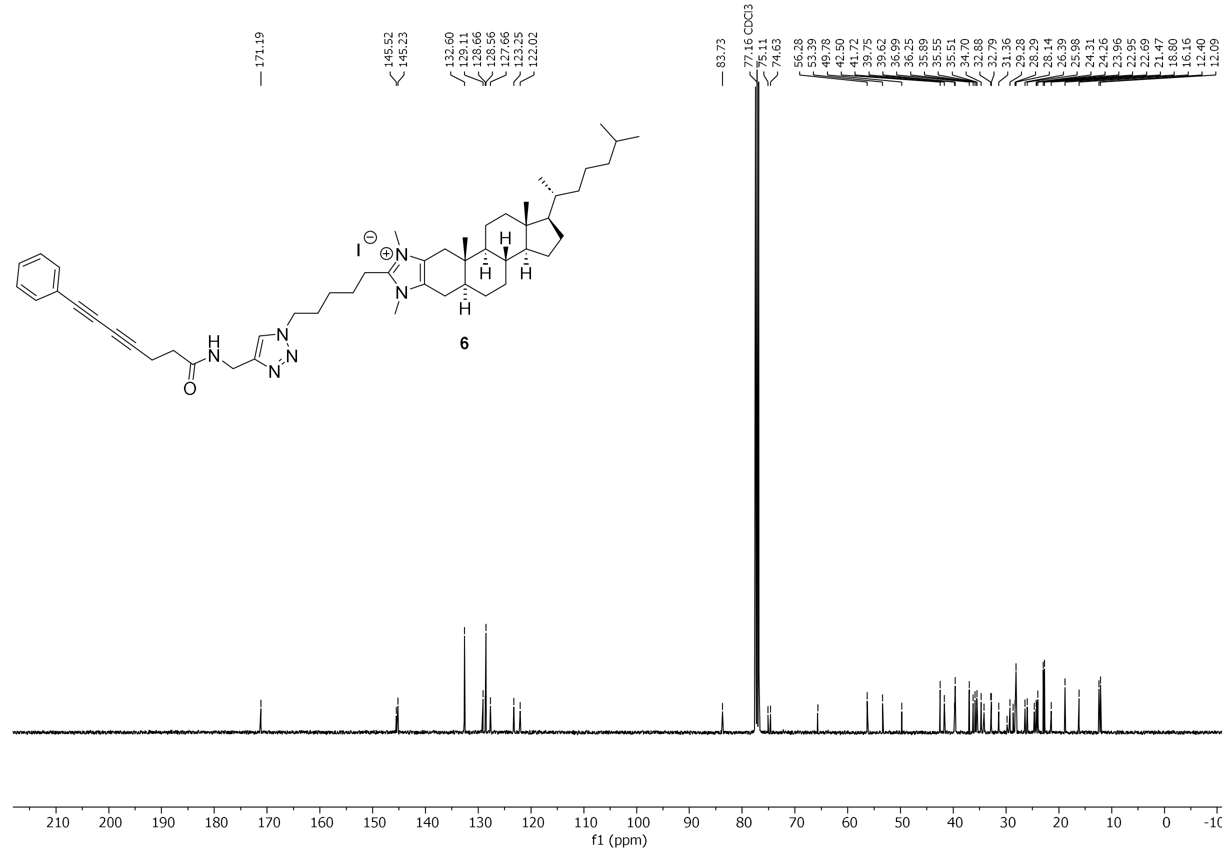
171.19
145.52
145.23
132.60
129.11
128.66
128.56
127.66
123.25
122.02
83.73
77.16 CDCl3
75.11
74.63
56.28
53.39
49.78
42.50
41.72
39.75
39.62
36.99
36.25
35.89
35.55
35.51
34.70
32.88
32.79
31.36
29.28
28.29
28.14
26.39
25.98
24.31
24.26
23.96
22.95
22.69
21.47
18.80
16.16
12.40
12.09
6
f1 (ppm)

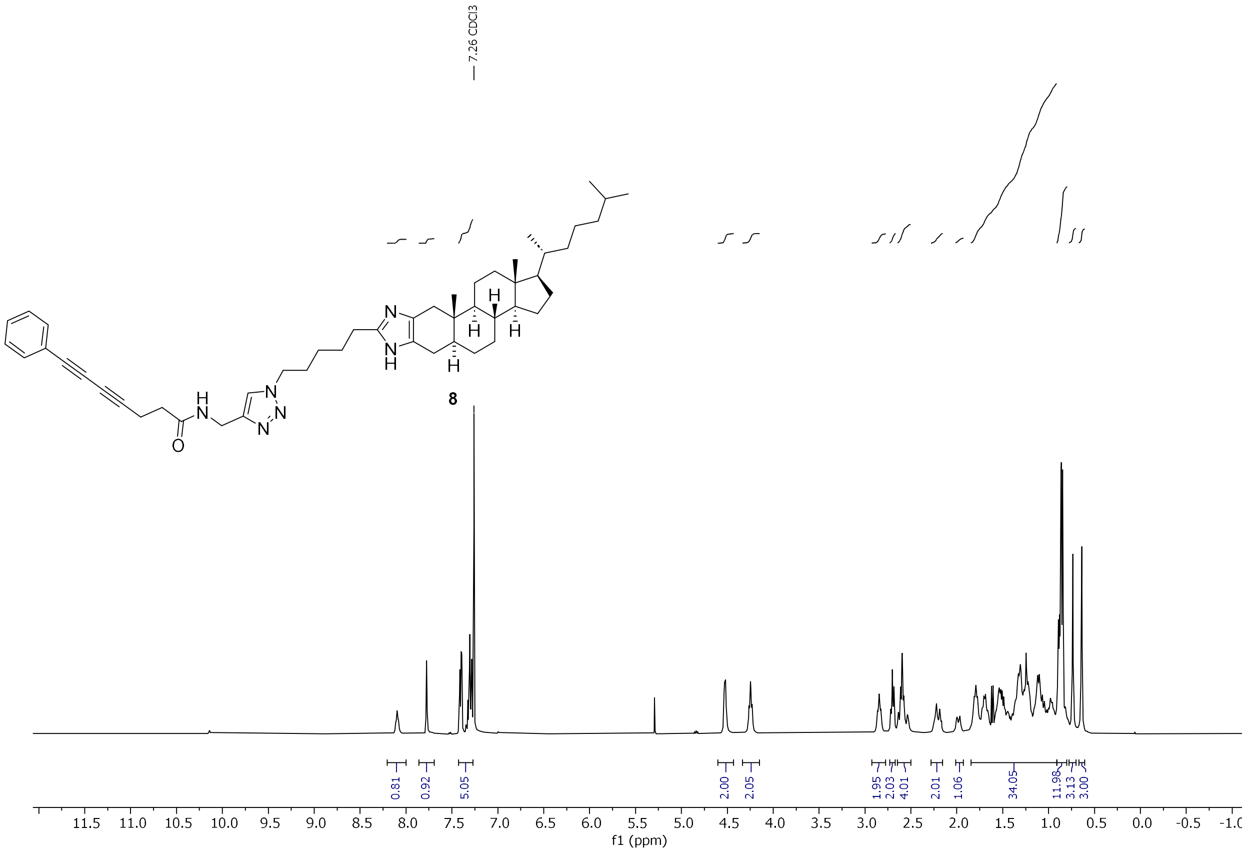
7.26 CDCl3
8
0.81
0.92
5.05
2.00
2.05
1.95
2.03
4.01
2.01
1.06
34.05
11.98
3.13
3.00
f1 (ppm)

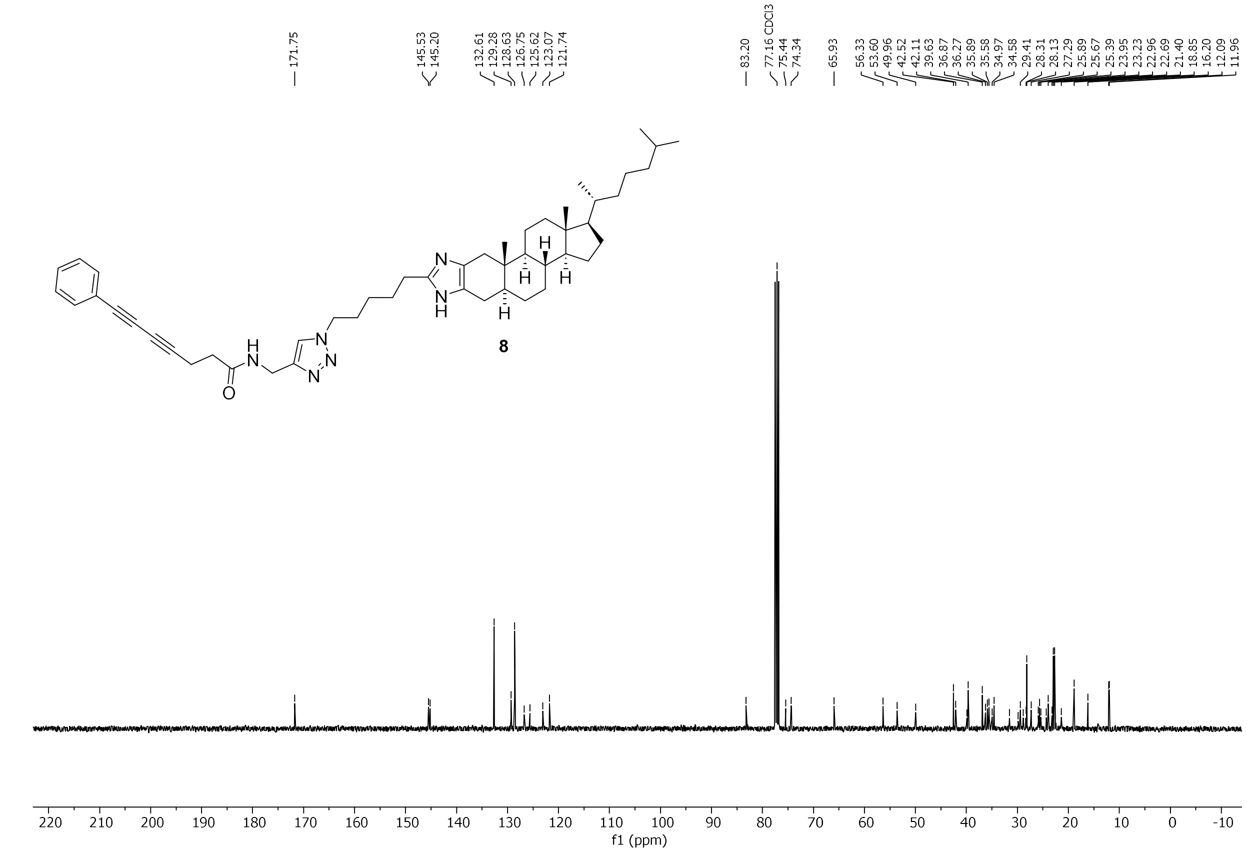
171.75
145.53
145.20
132.61
129.28
128.63
126.75
125.62
123.07
121.74
83.20
77.16 CDCl3
75.44
74.34
65.93
56.33
53.60
49.96
42.52
42.11
39.63
36.87
36.27
35.89
35.58
34.97
34.58
29.41
28.31
28.13
27.29
25.89
25.67
25.39
23.95
23.23
22.96
22.69
21.40
18.85
16.20
12.09
11.96
8
f1 (ppm)

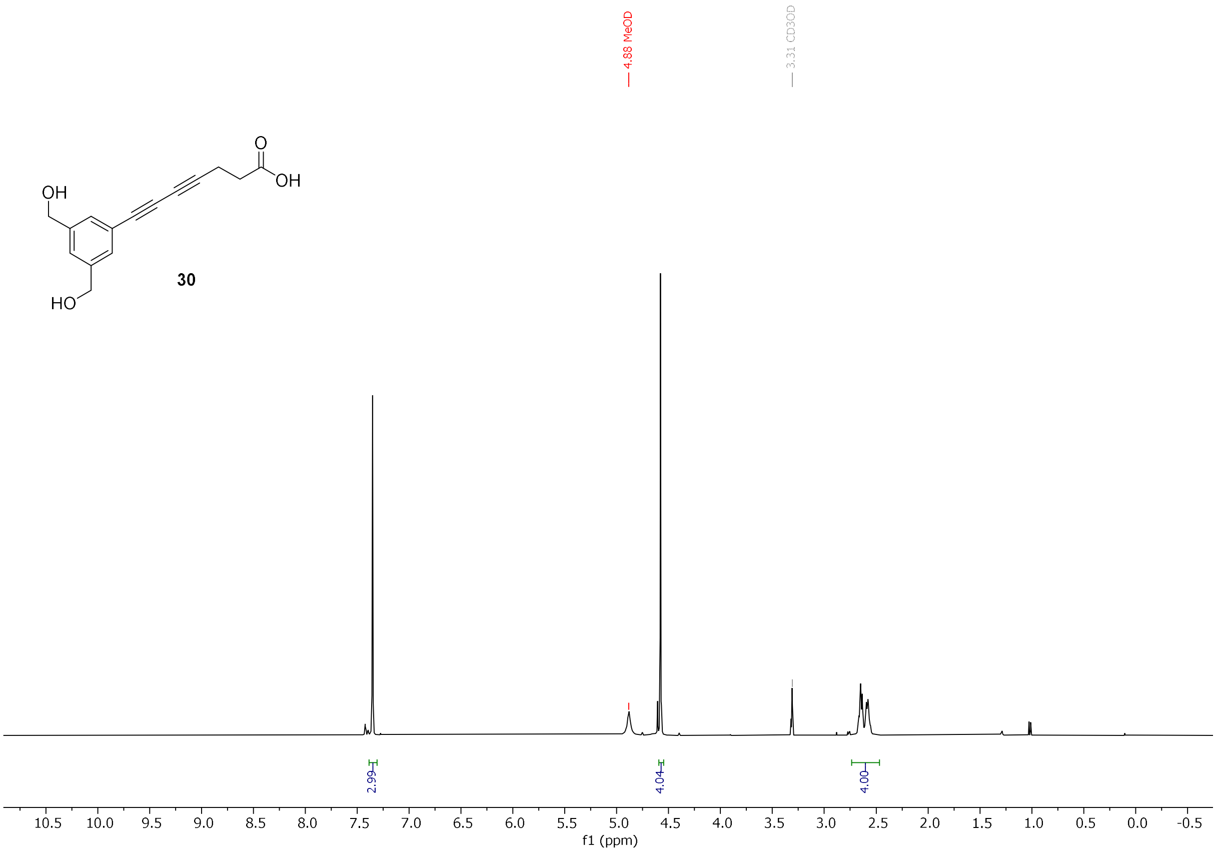
4.88 MeOD
3.31 CD3OD
OH
O
OH
HO
30
2.99
4.04
4.00
10.5 10.0 9.5 9.0 8.5 8.0 7.5 7.0 6.5 6.0 5.5 5.0 4.5 4.0 3.5 3.0 2.5 2.0 1.5 1.0 0.5 0.0 -0.5
f1 (ppm)

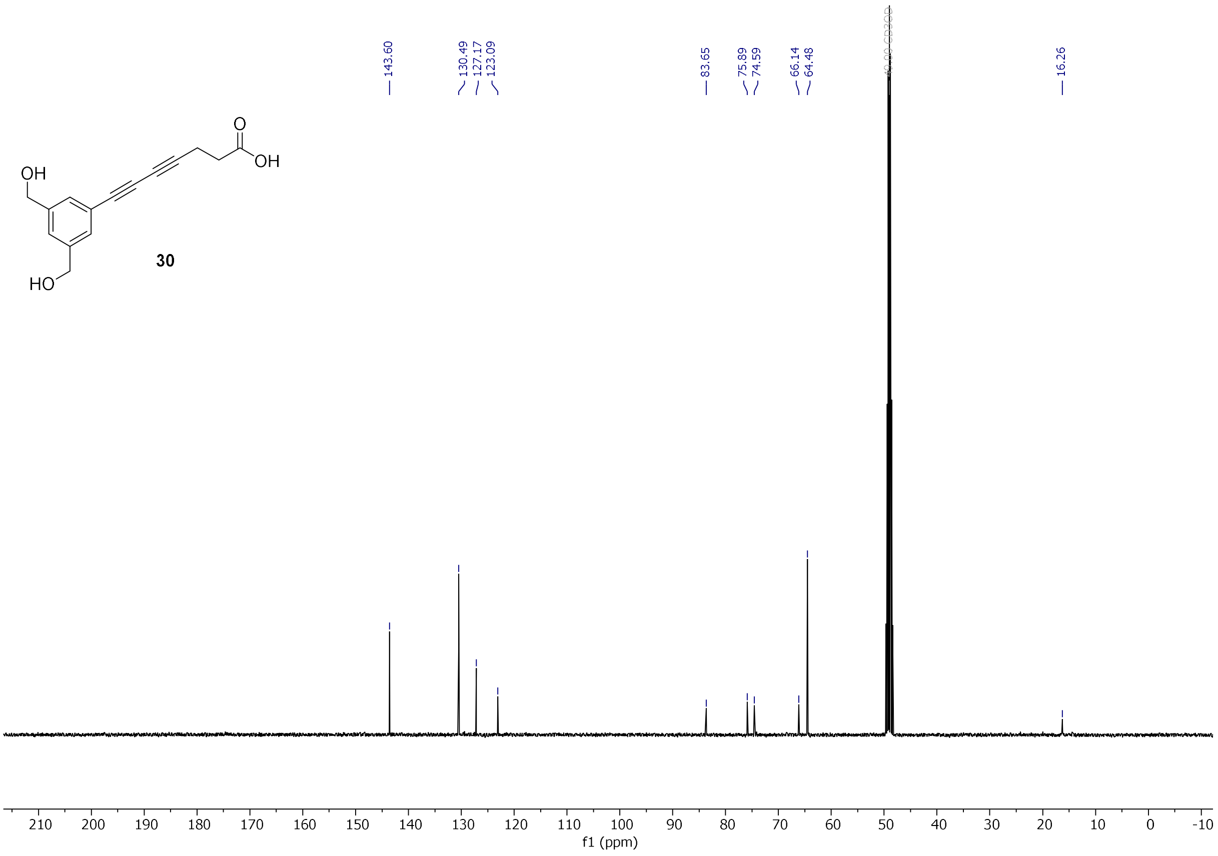
143.60
130.49
127.17
123.09
83.65
75.89
74.59
66.14
64.48
16.26
OH
O
OH
HO
30
210 200 190 180 170 160 150 140 130 120 110 100 90 80 70 60 50 40 30 20 10 0 -10
f1 (ppm)

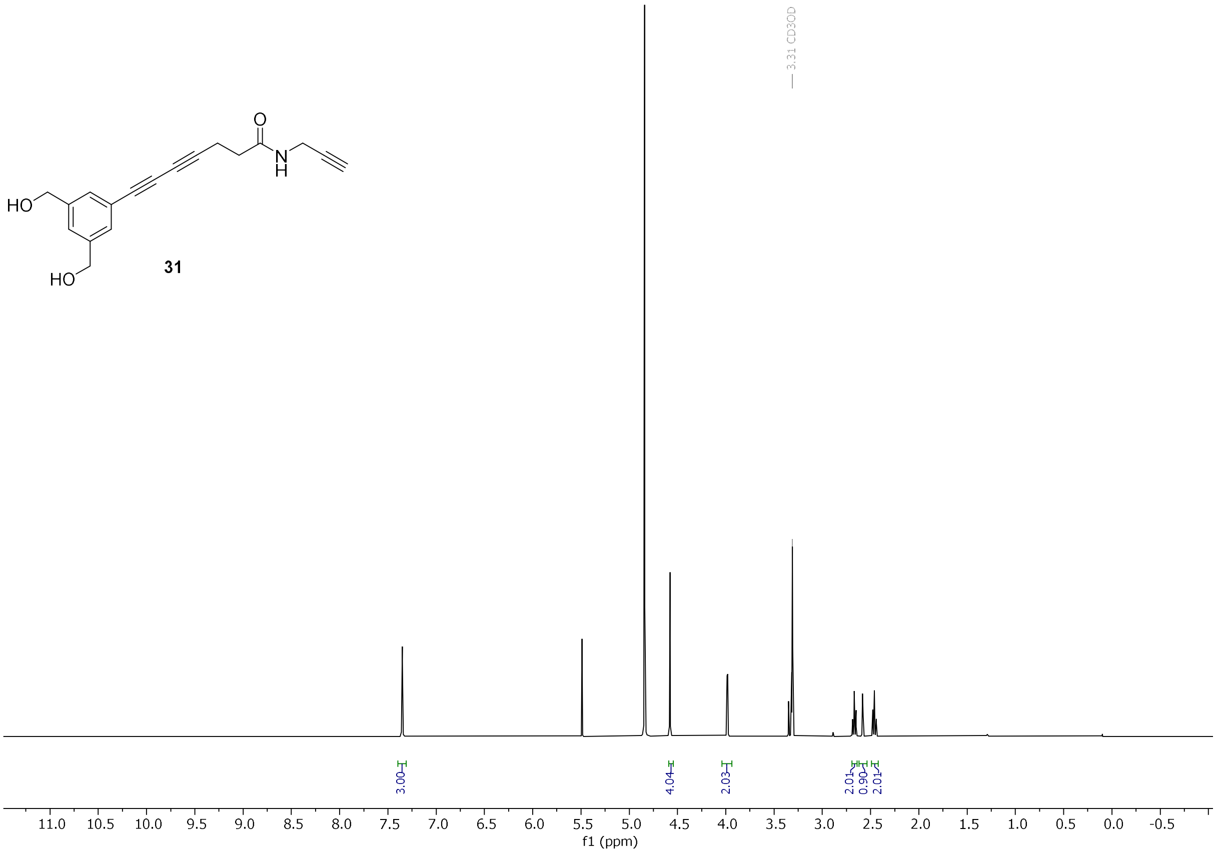

O
N
H
HO
HO
31
3.31 CD3OD
3.00
4.04
2.03
2.01
0.90
2.01
11.0 10.5 10.0 9.5 9.0 8.5 8.0 7.5 7.0 6.5 6.0 5.5 5.0 4.5 4.0 3.5 3.0 2.5 2.0 1.5 1.0 0.5 0.0 -0.5
f1 (ppm)

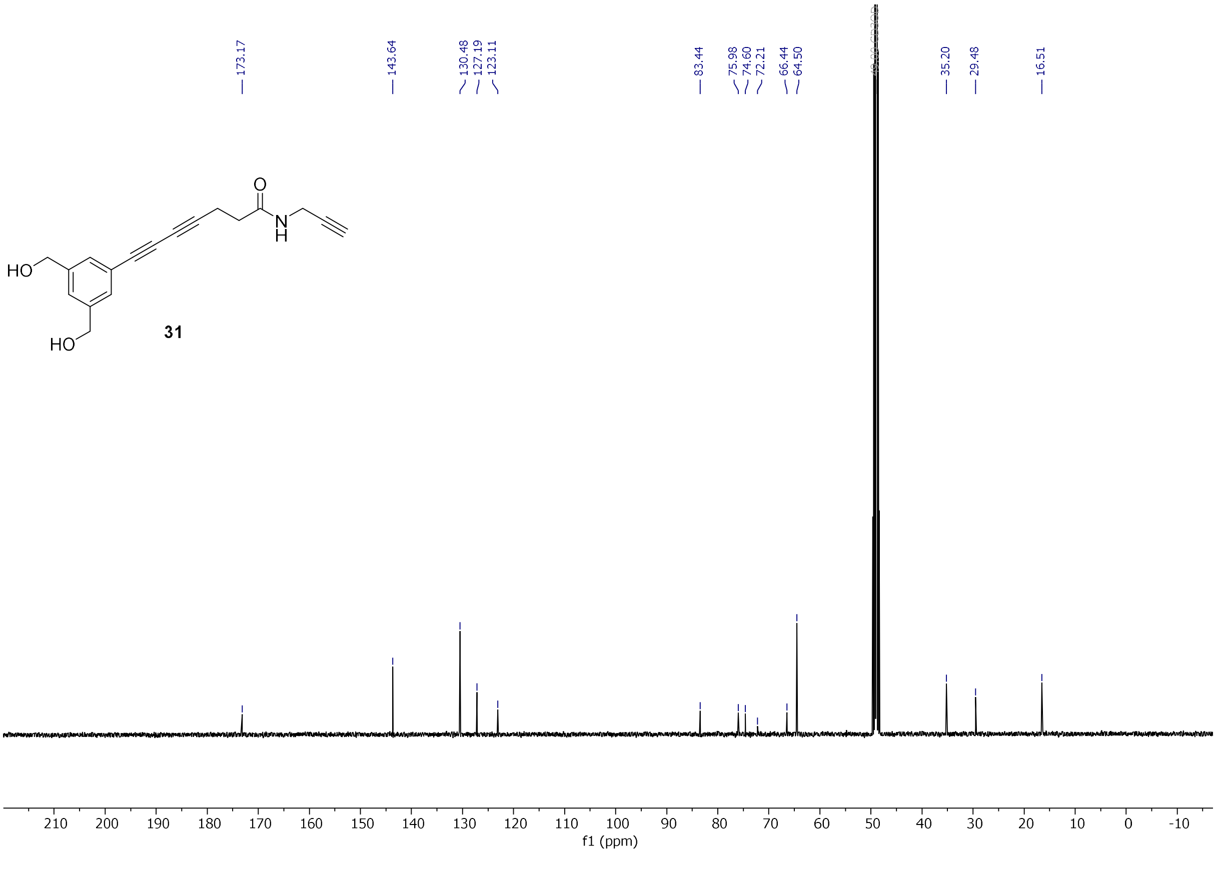

173.17
143.64
130.48
127.19
123.11
83.44
75.98
74.60
72.21
66.44
64.50
35.20
29.48
16.51
O
N
H
HO
HO
31
210 200 190 180 170 160 150 140 130 120 110 100 90 80 70 60 50 40 30 20 10 0 -10
f1 (ppm)

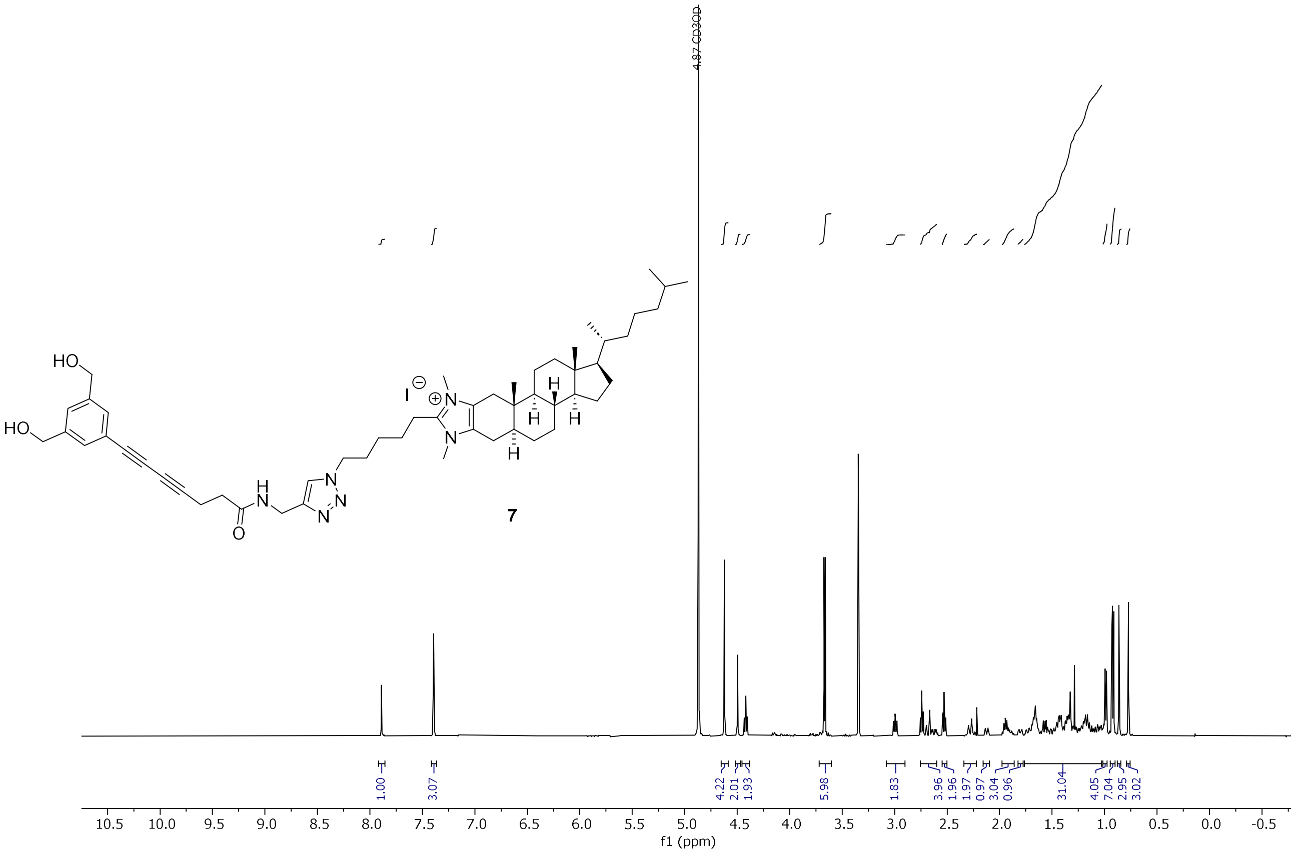
4.87 CD3OD
HO
HO
I
N
N
H
N
N
N
O
H
H
H
H
7
1.00
3.07
4.22
2.01
1.93
5.98
1.83
3.96
1.96
1.97
0.97
3.04
0.96
31.04
4.05
7.04
2.95
3.02
10.5 10.0 9.5 9.0 8.5 8.0 7.5 7.0 6.5 6.0 5.5 5.0 4.5 4.0 3.5 3.0 2.5 2.0 1.5 1.0 0.5 0.0 -0.5
f1 (ppm)

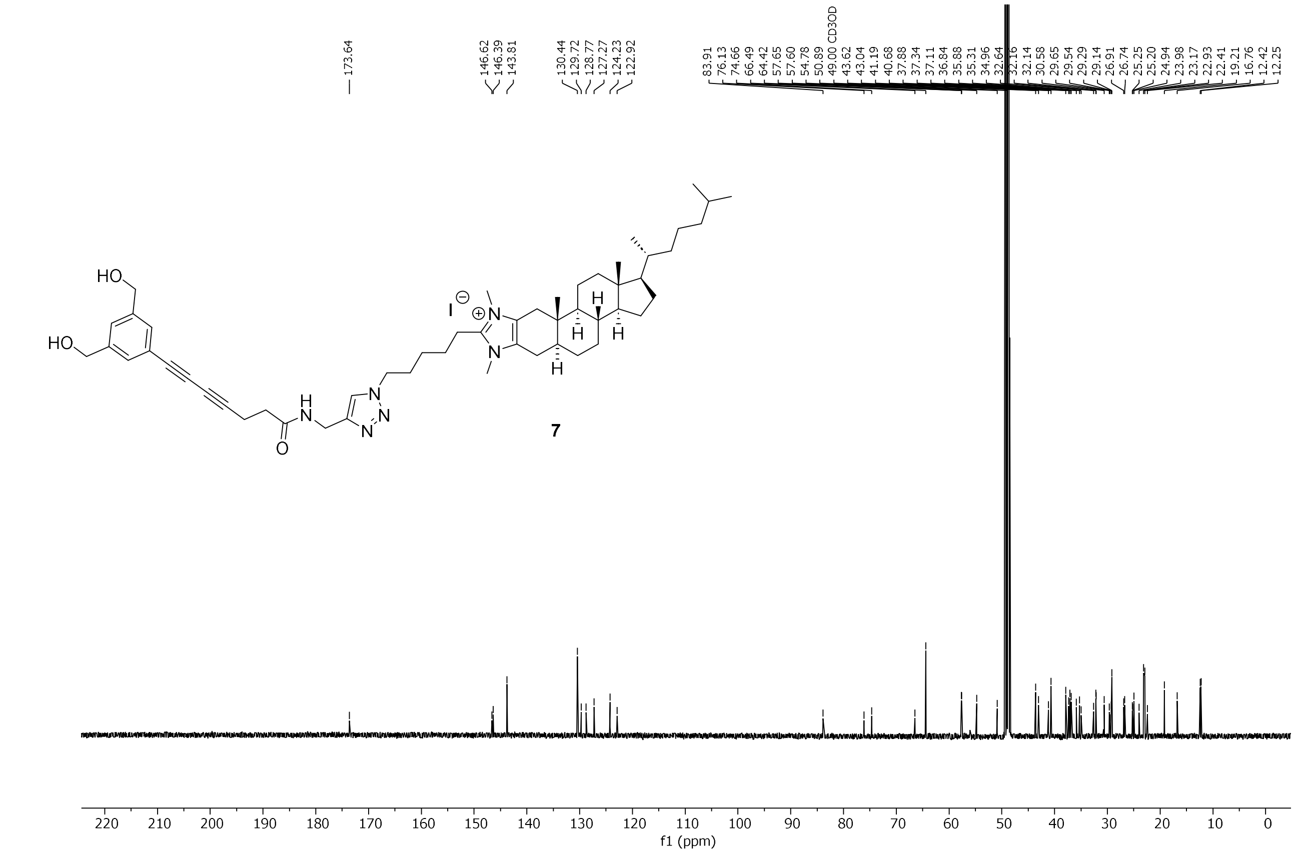
173.64
146.62
146.39
143.81
130.44
129.72
128.77
127.27
124.23
122.92
83.91
76.13
74.66
66.49
64.42
57.65
57.60
54.78
50.89
49.00 CD3OD
43.62
43.04
41.19
40.68
37.88
37.34
37.11
36.84
35.88
35.31
34.96
32.64
32.16
32.14
30.58
29.65
29.54
29.29
29.14
26.91
26.74
25.25
25.20
24.94
23.98
23.17
22.93
22.41
19.21
16.76
12.42
12.25
HO
HO
I
N
N
H
N
N
N
O
H
H
H
H
7
220 210 200 190 180 170 160 150 140 130 120 110 100 90 80 70 60 50 40 30 20 10 0
f1 (ppm)

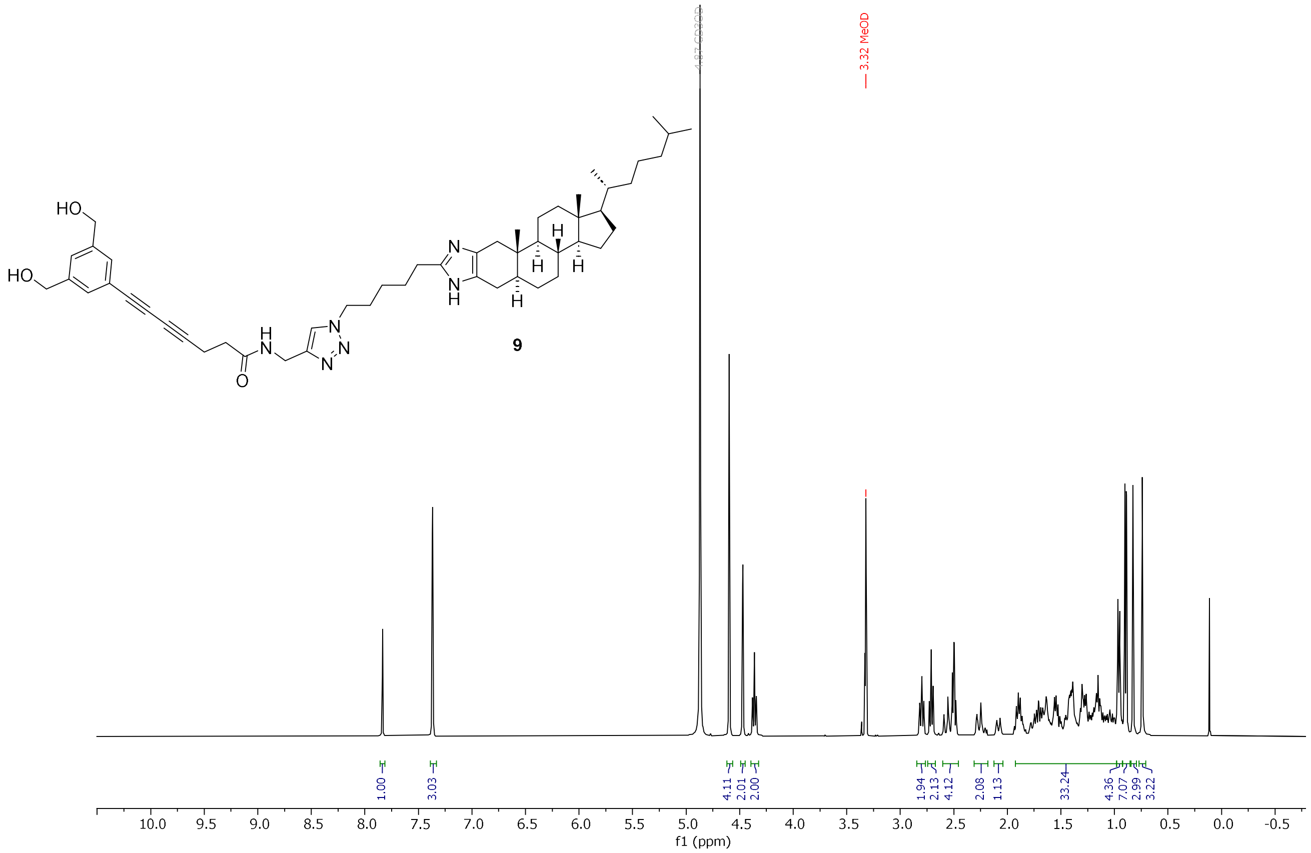

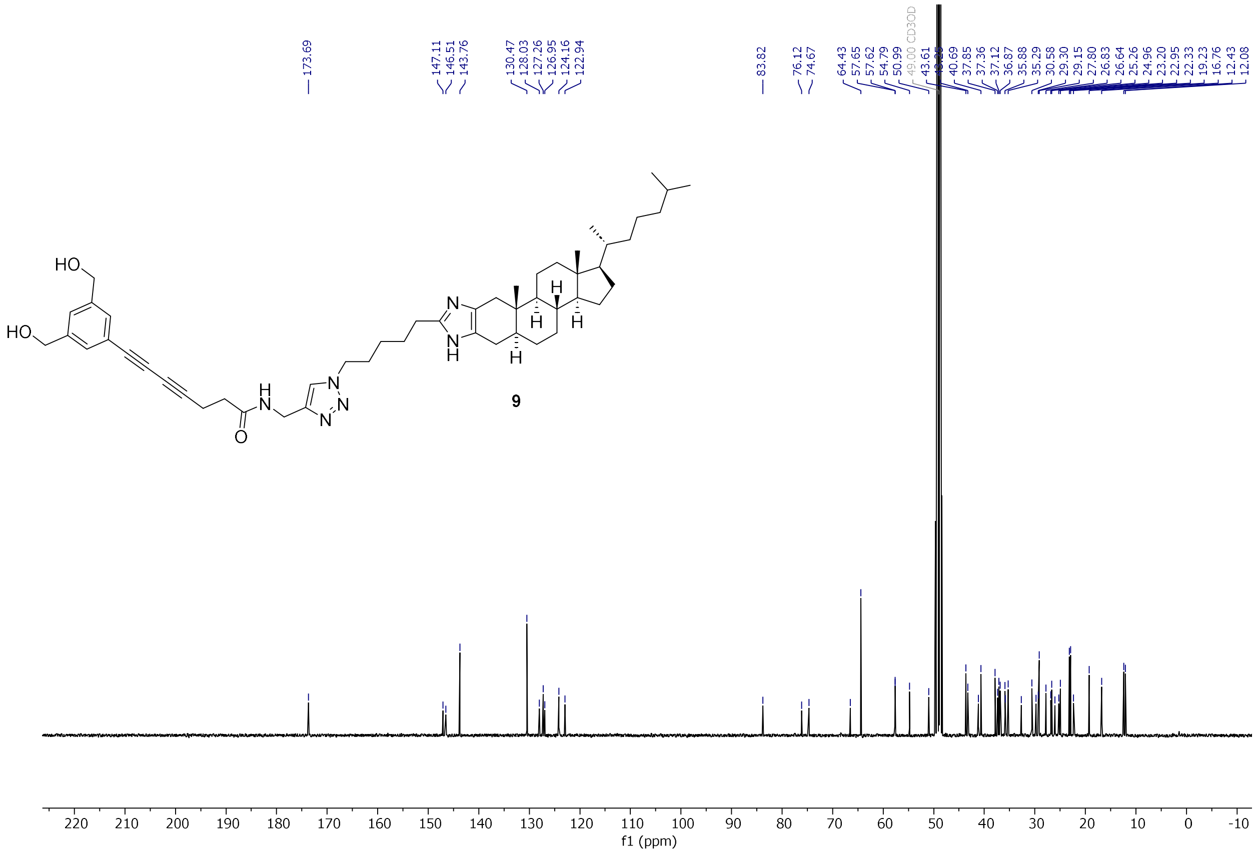

1.4 References

[1] L. Rakers, D. Grill, A. L. L. Matos, S. Wulff, Da Wang, J. Börgel, M. Körsgen, H. F. Arlinghaus, H.-J. Galla, V. Gerke, F. Glorius, *Cell Chem. Biol.* **2018**, *25*, 952-961.e12.

[2] A. S. K. Hashmi, R. Döpp, C. Lothschütz, M. Rudolph, D. Riedel, F. Rominger, *Adv. Synth. Catal.* **2010**, *352*, 1307–1314.

[3] S. Li, J. Tang, Y. Zhao, R. Jiang, T. Wang, G. Gao, J. You, *Chem. Comm.* **2017**, *53*, 3489–3492.

[4] S. Hiraoka, K. Hirata, M. Shionoya, *Angew. Chem. Int. Ed.* **2004**, *43*, 3814–3818.

[5] L. Su, J. Dong, L. Liu, M. Sun, R. Qiu, Y. Zhou, S.-F. Yin, *J. Am. Chem. Soc.* **2016**, *138*, 12348–12351.

[6] N. A. Danilkina, A. E. Kulyashova, A. F. Khlebnikov, S. Bräse, I. A. Balova, *J. Org. Chem.* **2014**, *79*, 9018–9045.

[7] A. I. Govdi, N. A. Danilkina, A. V. Ponomarev, I. A. Balova, *J. Org. Chem.* **2019**, *84*, 1925–1940.

[8] E. W. Crandall, L. Harris, *Org. Prep. Proced.* **1969**, *1*, 147–156.

[9] J. A. Wytko, J. Weiss, *Tetrahedron Lett.* **1991**, *32*, 7261–7264.

[10] Y. Gao, G. Wu, Q. Zhou, J. Wang, *Angew. Chem. Int. Ed.* **2018**, *57*, 2716–2720.

[11] X. Nie, G. Wang, *J. Org. Chem.* **2006**, *71*, 4734–4741.

II. Raman measurements

2.1 Additional details on the tags' Raman properties

2.1.1 Influence of the tag design on the stretching band frequency and band structure

The influence of the design on the spectral position of the triple bond's stretching frequency was determined by FT-Raman measurements of the solids (CHIMs **1-2** and **5-9,** Figure 2B) and BCARS measurements of DMSO-CHIM solutions (CHIMs **1-4**, Figure S 4). All measured CHIMs show bands between 2100 cm^{-1} and 2275 cm^{-1} with their largest peak located between 2210 cm^{-1} and 2250 cm^{-1}, which is a typical spectral region for alkyne-tagged molecules.[1] In the case of the diynes, a second band arises in the solid spectra (Figure 2B, **5-9**) which originates from the two possible vibrational modes; the antisymmetric and symmetric stretching vibration of the diyne structural element (Figure S1). Since the latter vibration causes larger changes in the polarizability, it yields stronger Raman intensities.

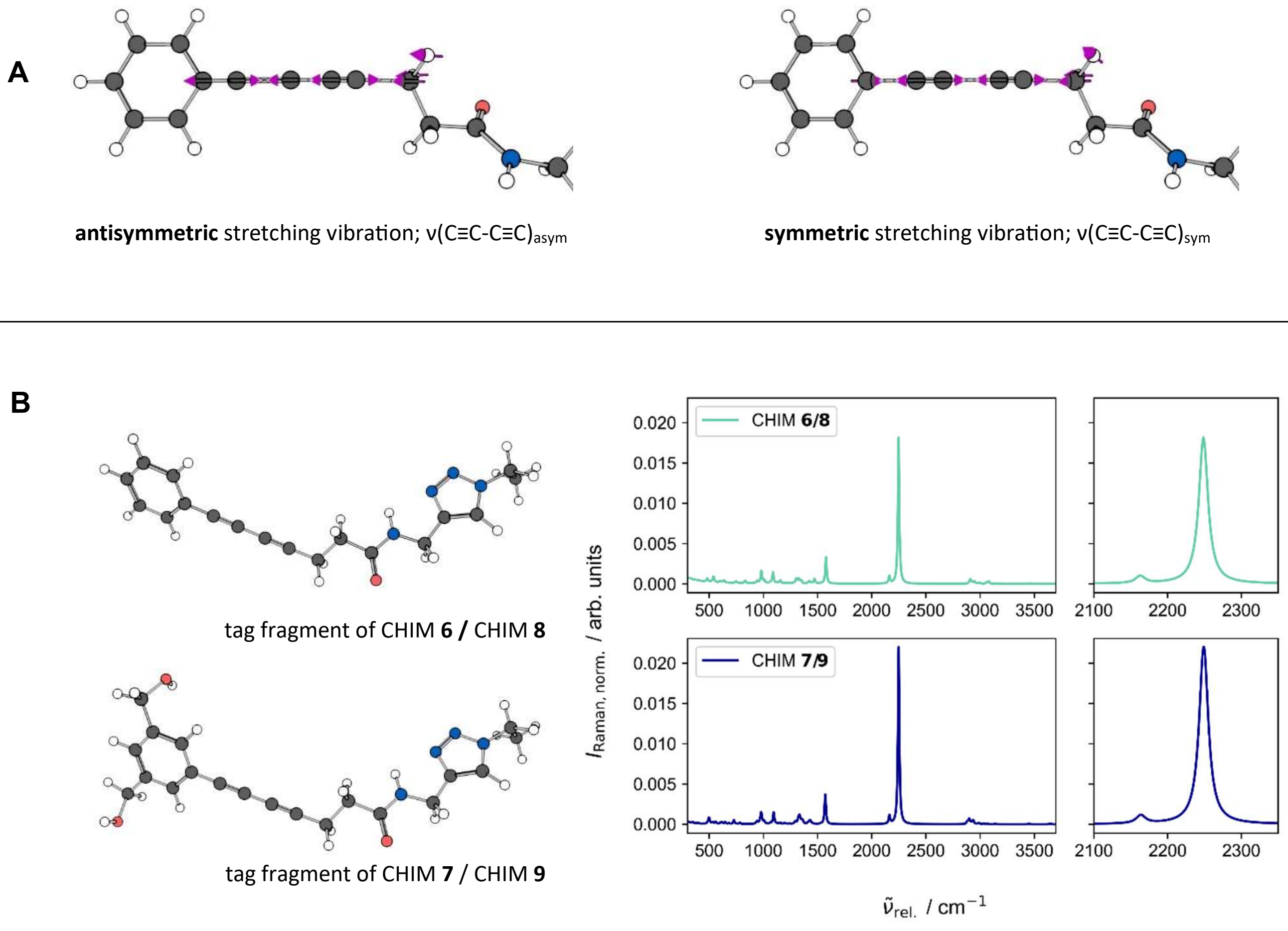

Figure S 1: Vibrational features predicted by DFT calculations on a tag fragment. (**A**) Atom movement during vibration, visualized by scaled displacement vectors. The diyne structural element possesses two vibrational modes in the silent region that are characterized by the antisymmetric and symmetric stretching vibration of the triple bonds. (**B**) Optimized tag structures and corresponding predicted Raman spectra for the CHIMs with diyne moiety and broken conjugated system. The cause for the measured differences in the bands not linked to the symmetric stretching vibration (cf. Figure 2) is not expected from quantum chemical calculations.

Based on literature reports, tuning of the band position of the symmetric alkyne stretching vibration is possible by variations in the number of conjugated triple bond units as well as by the addition of substituents, particularly with electron donating or withdrawing character in *ortho*- or *para*-position.[1,2] The negligible band shift between the alkyne-tagged CHIMs **1** and **2** (Figure 2B), however, indicates that CH_2OH decorations in *para*-position have no appreciable effect on the alkynes' stretching band position (both at 2218 cm^{-1}) which is likely due to their much weaker +M-effect compared to the earlier reported -OH and -NH_2-groups. At the same time, the incorporation of polar substituents provides a feasible toolbox for controlling and optimizing the water solubility of the molecule without the need to invent a new design from scratch. Hydroxymethyl groups are thus an optimal choice as water solubility becomes a function of the degree of substituents while spectral properties stay untouched.

In contrast, larger variations of water solubility and spectral features became apparent for alterations in conjugation length. The linear Raman spectrum of the fully conjugated diyne **5** is significantly shifted to lower wavenumbers compared with the fully conjugated alkyne version **1** and **2** (Figure 2B, 2218 cm^{-1} → 2208 cm^{-1}). CARS measurements performed for the four analogs **1**-**4** in DMSO solution revealed as well between a shift the very dark lower relative wavenumber diynes **3** and **4** and brighter higher relative wavenumber alkynes **1** and **2** of roughly 10 cm^{-1} (Figure S 4).
Similarly, an opposite direction shift is evident in the broken conjugation CHIMs **6-9**, which were developed to further increase water solubility by imparting greater tag flexibility while leaving the degree of conjugated triple bonds unaffected. Since a decreasing conjugation length leads to lower delocalization of the electrons of the triple bond unit, the bonds' spring constant *k* is significantly increased. The triple bond stretching bands therefore strongly shift to higher relative wavenumbers ($\tilde{\nu}_{vib} \propto \sqrt{k}$), now dominating at (2243 ± 3) cm^{-1}. The antisymmetric stretching band is furthermore strongly suppressed. While the frequency of the symmetric stretching vibration is only slightly affected by the additional hydroxymethyl decorations, sizably changes in the asymmetric stretching band occurred that we cannot explain from a theoretical point of view (Figure S 1).

In addition, we compared the CHIM core to a slightly modified imidazole-based scaffold (**8,9**). It was reasoned that the substitution of the permanently charged imidazolium salt by an imidazole (that exhibits a pH-dependent charge and can undergo hydrogen bonding instead of a permanent positive charge that can undergo ionic interactions) could affect solubility as well as membrane interaction and integration of the compounds. CHIMs **8** and **9** yield spectral features highly comparable with their respective imidazolium versions. As the imidazoles proved to be unsuitable in cell experiments, they are briefly shown here only for the sake of completeness.

2.1.2 Comparison of the tags’ efficiency between CHIMs

Relative scattering cross-sections between different CHIMs were determined by FT-Raman measurements of equally concentrated DMSO solutions (Table S 1). The method was validated by measurement of a concentration series between 0.0 mM and 47.0 mM, showing linear behavior, both in terms of band heights and areas under the band (Figure S 2). Three CHIMs, each representing one distinct design step (simple alkyne **1**, extension of the triple bond chain with simultaneously improved water solubility **5** and breakup of the conjugated system while retaining the diyne moiety **7**) served as the model systems for estimating the tag’s efficacy.

Estimation of the relative scattering cross-section based on the area **Estimation of the relative scattering cross-section based on height**

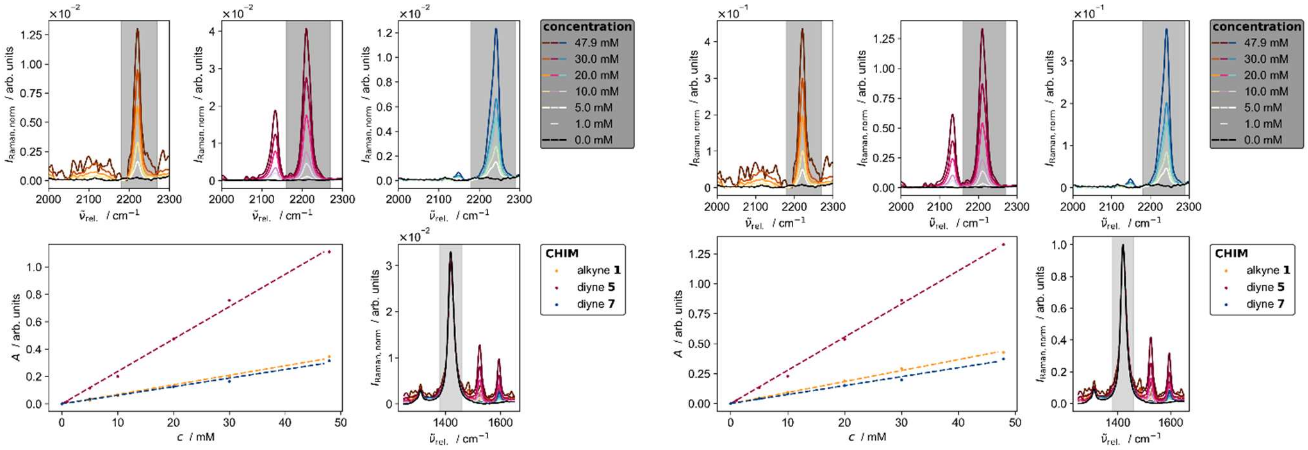

Figure S 2. Comparison of the Raman cross-section of three differently tagged CHIMs in DMSO. Relative Raman-intensities of the triple bond stretching band are shown for various concentrations of alkyne **1** (**B,** fully conjugated), diyne **5** (**A**, fully conjugated), and diyne **6** (**C**, partly conjugated) in DMSO after background correction and normalization of the area under the curve (left, integration range in gray) or band height (right) for the DMSO C-H-bending vibration at ≈1420 cm^{-1} (**D**). Plotting of the area under the curve/height of the C≡C stretching vibration in the highlighted regions against the concentration in DMSO gave rise to linear functions (**E**) enabling evaluation of the tag’s efficiency. A diyne in full conjugation shows the best results while breakage of the conjugated system or replacement of the diyne element by an alkyne both lowered the relative scattering cross section by ≈ 66%.

The relative scattering cross-sections were calculated from the quotient of the slopes of the substance in question and a reference. For comparisons between CHIMs only, the reference was chosen to be the first-developed alkyne CHIM **1** while for comparisons to other tagged substances, EdU served as the reference. The results and parameters needed for the calculation (Equation 1) and the maximum error estimate (Equation 2) are summarized in Table S 1 and Figure S 3B.

$$\sigma_{rel} = \frac{a_i}{a_{\text{ref}}} \qquad (1.)$$

$$\Delta\sigma_{rel} = \frac{1}{a_{\text{ref}}} \cdot \left(\Delta a_i + \frac{a_i}{a_{\text{ref}}} \Delta a_{\text{ref}}\right) \qquad (2.)$$

where a is the slope of in linear equation of type: $y = a\,x + n$ and the error of the slope was estimated as the square root of the corresponding entry in the covariance matrix.

Table S 1. Estimation of the relative scattering cross-section for three CHIMs, each representing one distinct design variation, in DMSO. Slope *a* and its error are used for the calculation of the relative scattering cross-section. The parameters were extracted from linear functions of type $y = a\, x + 0$ (Figure S 2) with x being the molar concentration c and y being the area under the band A or the band height h.

**Substance**	**Evaluation based on the band area A**			**Evaluation based on the band height h**		
	Slope a_i	Standard deviation of the slope $S(a_i)$	Relative scattering cross-section[a,b]	Slope a_i	Standard deviation of the slope $S(a_i)$	Relative scattering cross-section[a,b]
**CHIM 5**	0.02364	0.00043	3.40 ± 0.14	0.02771	0.00041	3.02 ± 0.10
**CHIM 1**	0.00695	0.00016	1.00 ± 0.05	0.00917	0.00015	1.00 ± 0.03
**CHIM 7**	0.00627	0.00020	0.90 ± 0.05	0.00747	0.00023	0.81 ± 0.04

[a] Errors were calculated by maximum error estimate using the standard deviation (square root of the elements of the covariance matrix) of the slope of the fitted linear function ($y = ax+0$) as the margin of error.

[b] Due to underlying noise in the case of **1** minor variations in the relative scattering cross-sections between both normalization approaches occur. The ratio of the relative cross-sections of CHIMs **5** and **7** is nevertheless matching well.

2.1.3 Comparison of the tags' efficiency to other tagged molecules

As introduced by Yamakoshi *et al*. the relative intensity versus EdU (RIE)[1] allows a fair comparison of the tag's efficiency in contrast to the often-used normalization to another intrinsic band. The use of cheap commercially available external standards, such as EdU, is widely applicable and allows a common origin to be established for a scale of label efficiency which is the foundation of comprehensible comparative evaluations. Due to the aforementioned reason, it is unfortunately not possible for us to make direct comparisons with published alkyne-tagged cholesterols.[3] Nevertheless, indirect comparisons are still possible thanks to known ranges of RIEs for various molecular designs. Our determined values for both, CHIMs and reference substances (Figure S 3), fit well into the known scale, confirming our molecular design to be among the best scattering tag arrangements. For similar reasons, it seems to be reasonable to claim that our innovative head group-modified cholesterol mimicker of type Ar-≡-R, respectively Ar-≡-Ar, is very likely superseding or at least equalizing the reported intensity of the previously published tail-tagged analog of type Ar-≡-R [3].

A

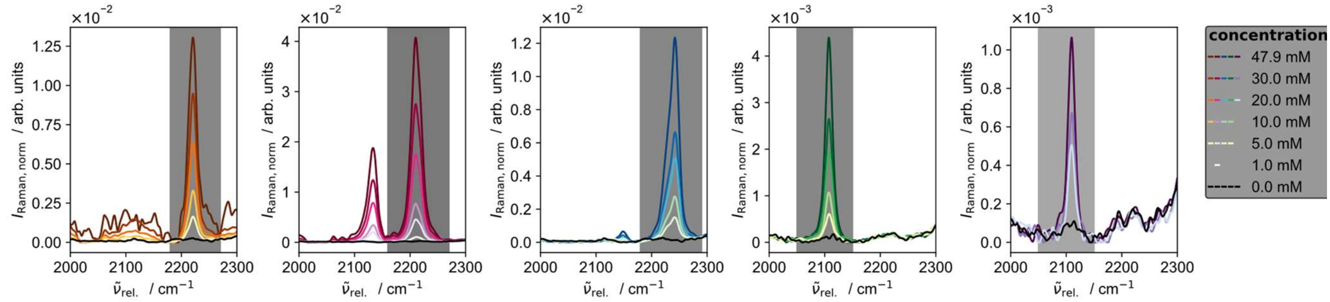

B

Substance	Evaluation based on the band area A		
	**Slope a_i**	**Standard deviation of the slope $S(a_i)$**	**RIE**
**CHIM 5**	0.02364	0.00043	14.69 ± 0.44
**CHIM 1**	0.00695	0.00016	4.32 ± 0.15
**CHIM 7**	0.00627	0.00020	3.89 ± 0.17
**EdU**	0.00161	0.00002	1.00 ± 0.02
**17-ODYA**	0.00032	0.00002	0.20 ± 0.01

C

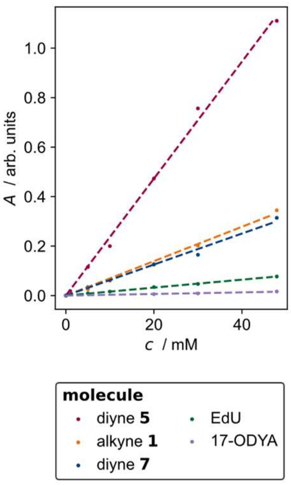

Figure S 3. Comparison of the CHIMs scattering cross-section in DMSO with one of two commercially available alkyne-tagged small molecules (nucleoside EdU and fatty acid 17-ODYA). (**A**) Raman intensity of the alkyne/diyne (symmetric stretching band) for various concentrations of the respective molecules in DMSO after background correction and normalization by area to the band of the DMSO C-H bending vibration (≈1420 cm^{-1}). For the sake of comparability with literature-reported values, the relative scattering cross-sections were calculated with respect to EdU (RIE). The calculation is based on the slope *a* of linear functions of type *A(c) = ac* (**C**). The extracted fitting parameters and the calculated RIE are summarized in the table (**C**). All synthesized CHIMs possess superb RIEs that are up to 73× higher than the one of the alkyne-tagged fatty acid 17-ODYA.

2.2 BCARS measurements

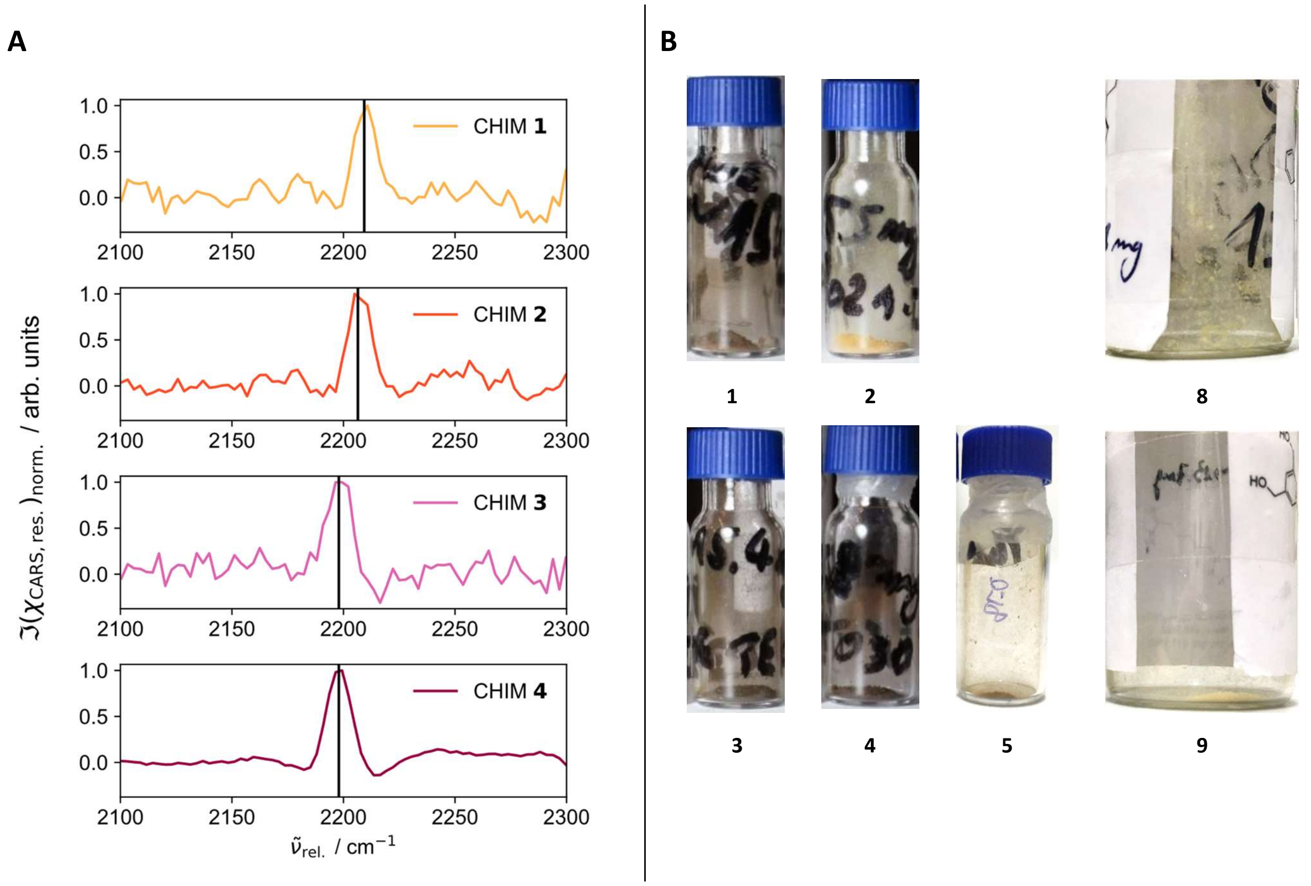

Figure S 4: A: Reconstructed BCARS spectra of CHIMs **1-4** depicting the normalized imaginary part of the nonlinear CARS susceptibility as a function of the difference frequency in the silent region. The center frequencies of the band of the diyne analogs (CHIMs **3** and **4**) are shifted by 10 cm^{-1} into the lower wavenumber range compared to their alkyne equivalents (CHIMs **1** and **2**). **B:** The synthesized CHIMs covered a large range of colors, ranging from off-white to dark brown powder. Samples **6** and **7** are not shown as being more viscid and widely spread to the glass wall. BCARS measurements proved especially beneficial for the darker diyne samples **3-4** allowing the detection of prominent vibrational bands in the darker samples, in contrast to measurements performed with linear Raman scattering.

Complementary to measurements using spontaneous linear Raman scattering (Figure 2B), vibrational spectra of CHIMs **1-4** were recorded using nonlinear coherent anti-Stokes Raman scattering (CARS) as well.

If a sample is illuminated with two spatially and temporally overlapping electromagnetic fields with their frequencies ν_{pump} and ν_{Stokes} tuned to match the beating frequency of a molecular bond vibration ($\nu_{\mathrm{vib}} = \nu_{\mathrm{pump}} - \nu_{\mathrm{Stokes}}$), oscillations of frequency ν_{vib} are efficiently driven. The efficacy depends on the degree of detuning between the bond's resonance frequency and the driving frequency. Probing the generated grid of coherently excited Raman modes with a third field (usually again the pump field) results in modulations of this third incoming field and light at the anti-Stokes frequency ($\nu_{\mathrm{CARS}} = 2\,\nu_{\mathrm{pump}} - \nu_{\mathrm{Stokes}}$) is emitted. Complex peak shapes arise due to the combination of the briefly described resonant and an additional non-resonant four-wave-mixing contribution ($\nu_{\mathrm{CARS}} + \nu_{\mathrm{Stokes}} = 2\,\nu_{\mathrm{pump}}$, independent of ν_{vib}). The imaginary part of the CARS-susceptibility's resonant contribution ($\Im(\chi_{CARS,res.})$) owns a Lorentzian profile with its center matching the spectral band position in spontaneous Raman spectra.[4] As the wavelength of the recorded signal is smaller than the excitation frequency, CARS measurements can be operated unaffected by single photon excited fluorescence (which is Stokes shifted). Hence, even the much darker samples of the diyne-tagged CHIMs **3** and **4** eventually showed prominent peaks (Figure S 4).

The measurements of the tagged-CHIMs **1-4** were conducted in quartz glass cuvettes with 1 mm path length (ρ_i=10 mg mL^{-1} in DMSO) on a previously characterized broadband CARS (BCARS) setup[5]. $\chi_{CARS,res.}$ was extracted in the spectral region between 2100 cm^{-1} – 2300 cm^{-1} by a Kramers-Kronig relation[6] followed by ALS-phase-correction[7,8] and a scaling-error-correction[7] as suggested by Cicerone *et al*. The nonresonant background required for the spectrum reconstruction was taken from a sample of pure DMSO which served as the control.

The reconstructed BCARS spectra of CHIMs **1-4** are depicted in Figure S 4A showing a shift of the center wavenumber between the alkyne- and diyne-tagged CHIMs.

2.3 Remarks on the prediction of the vibrational Raman spectra

DFT calculations on cholesterols were done with ORCA 5.0[14]. For the optimization and numeric frequency calculation of cholesterol-d$_6$, a Karlsruhe valence triple-zeta basis set with one set of polarization functions (def2-TZVP)[15] combined with a B3LYP-hybrid functional[16] and Grimme's DFT-D3(BJ) Becke-Johnson dispersion correction[17] was used. For the smaller tag fragments of CHIMs 7 and 9, the basis set was enlarged to a more costly Karlsruhe valence triple-zeta basis set with two sets of polarization functions and one set of diffuse functions (def2-TZVPPD)[15] paired with conducting the calculation additionally in Cartesian coordinates to cope for the 180° dihedral angles of the diyne structure. To speed up calculation, coulomb and Hartree-Fock exchange integrals were approximated by RIJCOSX[18] utilizing Weigend's def2/J auxiliary basis sets[19].

The calculated Raman activities (S_i) were transformed into intensities applying Equation (3) which is valid for plane-polarized light with the polarization axis of the incident beam of wavenumber $\tilde{\nu}_0$ being perpendicular to the scattering plane[20].

$$\frac{\left(\frac{\partial \sigma_i}{\partial \Omega}\right)}{(\tilde{\nu}_0 - \tilde{\nu}_i)^4} = \frac{2^4 \pi^4}{45} \cdot \frac{h}{8\pi^2 c \tilde{\nu}_i \cdot \left[1 - \exp\left(-\frac{hc\tilde{\nu}_i}{k_B T}\right)\right.} \cdot S_i \qquad (3.)$$

In this equation, $\left(\frac{\partial \sigma_i}{\partial \Omega}\right) \cdot (\tilde{\nu}_0 - \tilde{\nu}_i)^{-4}$ is the normalized differential scattering cross-section, c, h and k_B are natural constants (speed of light, Planck's and Boltzmann's constant), and T was assumed to be room temperature (T= 298.15 K, ϑ= 25 °C). The normalized differential scattering cross-section is independent of the wavenumber of the incident laser beam ($\tilde{\nu}_0$ = 9398.5 cm^{-1}, λ = 1064 nm, ν=281.8 THz for our experimental setup). The calculated frequencies were broadened to a Lorentzian peak profile with a full width at half maximum (FWHM) of 15 cm^{-1}. The calculated wavenumbers were corrected for errors caused by approximated electron correlation and unconsidered anharmonicity by multiplication with an empiric scaling factor (0.965 for B3LYP/def2-TZVP and 0.963 assumed for B3LYP/def2-TZVPPD).[21]

2.4 Labeling experiments with a commercially available tagged cholesterol

For comparison of the CHIMs' distribution in the cell to native cholesterol, the uptake experiments were repeated with a deuterated analog. Due to the large isotopic effect of deuterium, the corresponding vibrational frequencies are tremendously shifted compared to the ^{1}H-analog, now arising as well in the silent region. However, the overall molecular size remains the same. If deuterium is inserted as a part of the side chain severe changes in properties and membrane fluidity can be ruled out to the best of our knowledge[9], thus retaining the biological and amphiphilic behavior of natural cholesterol. Compared to the radiolabeled cholesterol versions, which are otherwise used as the most unperturbed and trackable reference substances[10], the multi-deuterated versions can still be detected by vibrational spectroscopy, permitting a straight comparison to the CHIM experiments.

Deuterated versions of cholesterol are commercially available with no custom synthesis until up to 7 labels per molecule. Tail-labeled cholesterol-26,26,26,27,27,27-d_6 was elicited as most suitable for our purpose by quantum chemical calculation. Due to symmetry reasons only a few clearly distinguishable bands are provided in the silent region, making this variant most promising for detecting even low signal strengths on a background (Figure S 5).

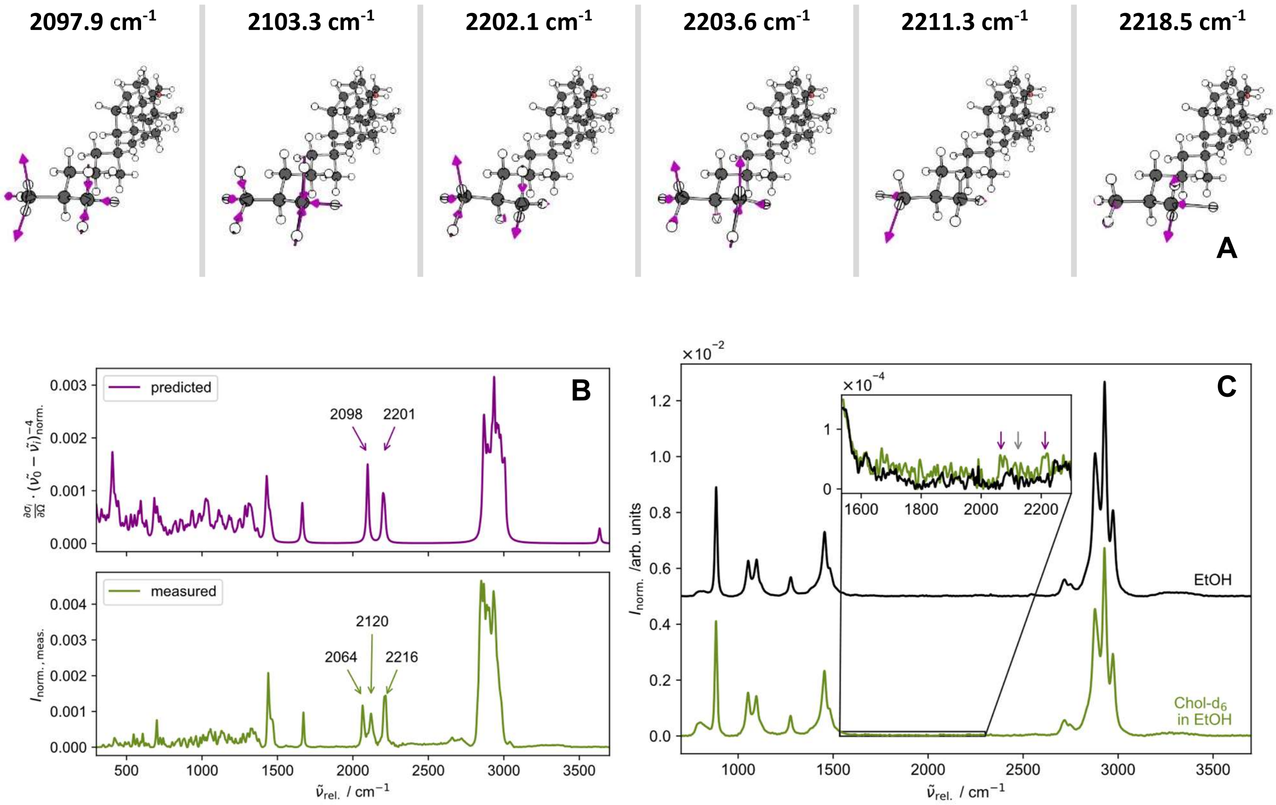

Figure S 5. Raman properties of cholesterol-26,26,26,27,27,27-d_6 with excitation at 1064 nm (A) Atom displacement (purple) of calculated Raman modes with frequencies in the silent region. (B) Raman spectra, retrieved from the measurement of the solid (green) and theoretically predicted spectrum (purple) and (C) obtained from a 47.9 mM solution in spectroscopy grade EtOH (green). The spectrum of pure EtOH is depicted in black. The silent region is enlarged in the inset, revealing slightly higher intensities at two of the three positions where bands of the C-D stretching vibration in the solid spectrum are observed (arrows). All spectra were normalized by a vector norm.

Due to the comparably low signal strength of deuterium compounds in comparison to alkynes[1,11] and particularly CHIMs, detection of the deuterium tag with Raman microspectroscopy inside cells was not possible for incubations with a 100 µM cholesterol solution. The limited solubility of cholesterol in an aqueous environment and the restricted tolerance of cells against non-aqueous solutions demanded careful modifications to our usual procedure (Experimental Section) to ensure enough uptake in a set time. As we intended to study the early distribution of cholesterols after uptake, long-term incubation

of several hours with low-concentrated treatment solutions as used for the investigation of cholesterol storage conditions is not appropriate[3,10]. Fast cholesterol incorporation is known to succeed within minutes for complexation with methyl-β-cyclodextrin (MβCD)[10]. Modifying the general complexation procedure of Leppimäki *et al.*[12] we pushed the uptake to a limit using a 500 µM cholesterol solution with a molar sterol/cyclodextrin ratio of 1 / 10 in serum-deficient medium (FluoroBrite™ DMEM) and an incubation time of 10 min at 37 °C, 5 % CO_2 which is the maximum we could afford in terms of cell response and toxicity effects. The treatment solution was sonicated for 1 h at 37°C and centrifuged for 5 min (18000 rpm) showing full complexation and no precipitation. The cells were starved for 45 min in serum deficient medium before the cholesterol treatment. The amount of ethanol as a cholesterol-dissolving solvent in the treatment solution (1 % v/v) can still be considered tolerable but should not be exceeded[13].

The distribution of the deuterium-labeled cholesterol in a cell is depicted in Figure S 6. Although the signal is much weaker than for our synthesized CHIMs, high accumulation of cholesterol can be specially identified in areas including the plasma membrane.

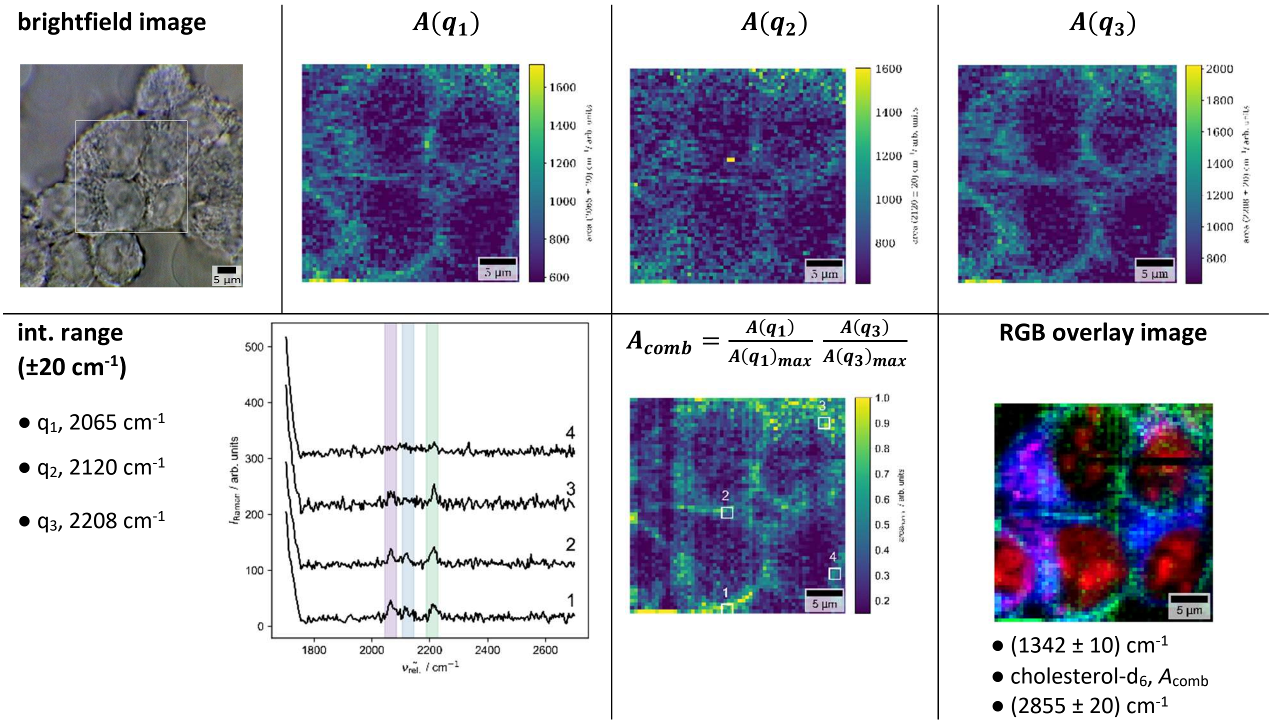

Figure S 6. Distribution of deuterated cholesterol in HEK cells. The cells were treated with a cholesterol-d_6-MβCD complex of 500 µM total sterol concentration and incubated for 10 min at 37 °C, 5 % CO_2. The areas of cholesterol accumulation were identified by integrating the area under the Raman spectrum (λ_{ex} = 514.6 nm) that corresponds to the C-D-stretching vibrations ($A(q_1)$, $A(q_2)$, $A(q_3)$). As the contrast is very low, a combined image was created by multiplying the normalized images of the two most intense bands (q_1 (symmetric) and q_3 (anti-symmetric)) eventually revealing the distribution of the cholesterol along the cell border. Average spectra of 4 representative positions at the cell border were generated from a 3 px × 3 px grid as indicated and show bands within the chosen integration ranges.

2.5 Supporting information for Figure 3

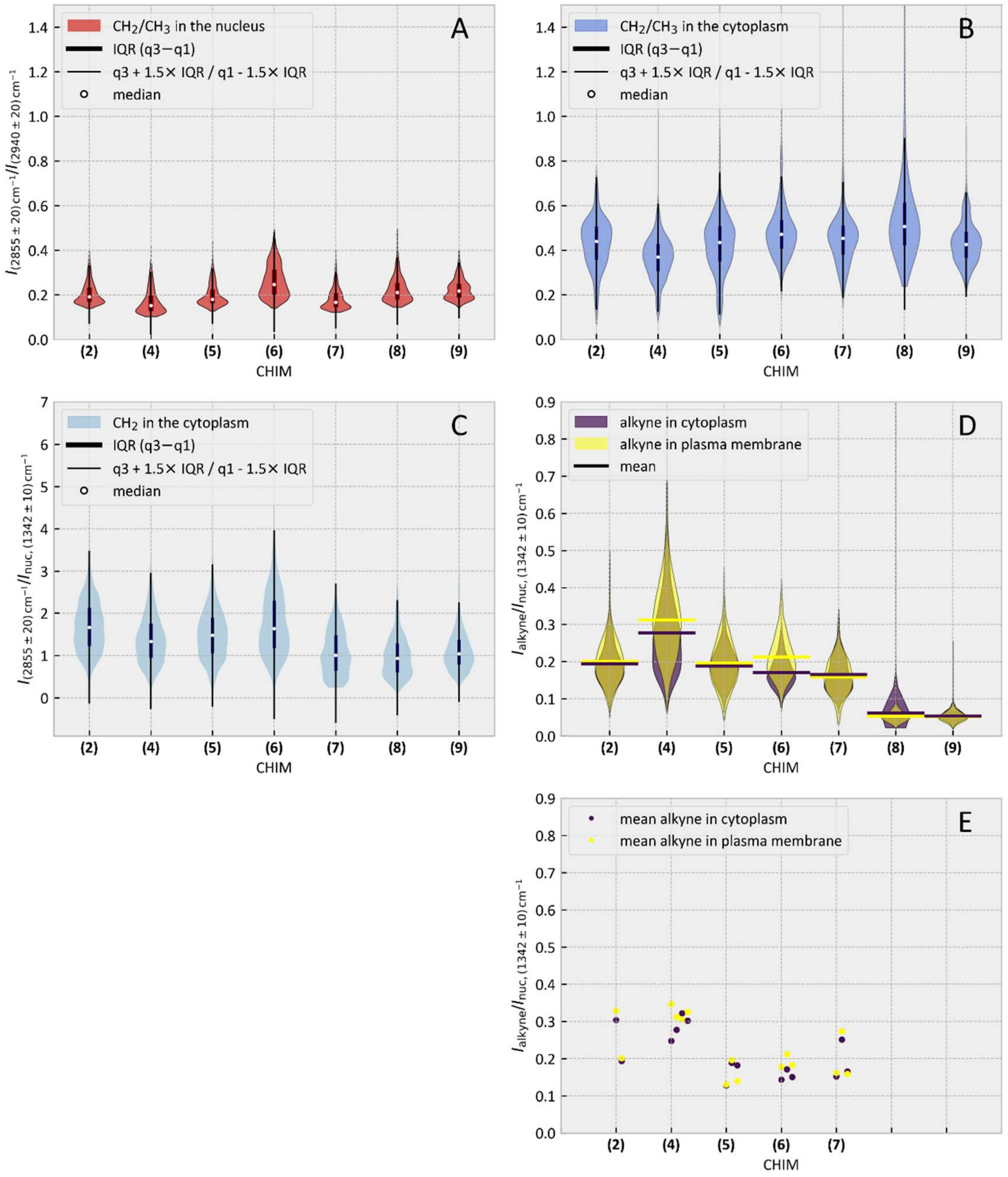

Figure S 7. Comparison of alkyne accumulation and validation of the signal content of the showcased examples. (A-B): Evaluation of the CH_2-to-CH_3-ratio per pixel in the nucleus (A) and the cytoplasm (B) of the cells showcased in Figure 3. C: Evaluation of the CH_2 content in the cytoplasm per pixel with reference to the mean nucleus signal as used for scaling in Figure 3. D: Evaluation of the local alkyne accumulation in the outer plasma membrane per pixel. For referencing purposes, the obtained counts were referenced to the average nucleus signal as used for scaling in Figure 3. E: Intrabatch variation of mean values for the measured positions, for more information cmp D. Marker at the same x-position belong to the same position analyzed. CHIM **6** shows the strongest confinement to the plasma membrane, while the overall signal per pixel of CHIM **3** is highest. CHIMs **8** and **9** do not accumulate in the cells. The following integration ranges were used for extraction of the alkyne signal: CHIM **2**: (2221±20) cm^{-1} CHIM **3-4**: (2212±20) cm^{-1}, CHIM **6-7**: (2243±20) cm^{-1}, CHIM **8-9**: (2245±20) cm^{-1}, IQR: inter quantile range, q3: upper quantile, q1: lower quantile.

2.6 Determination of the *in-situ* limit of detection

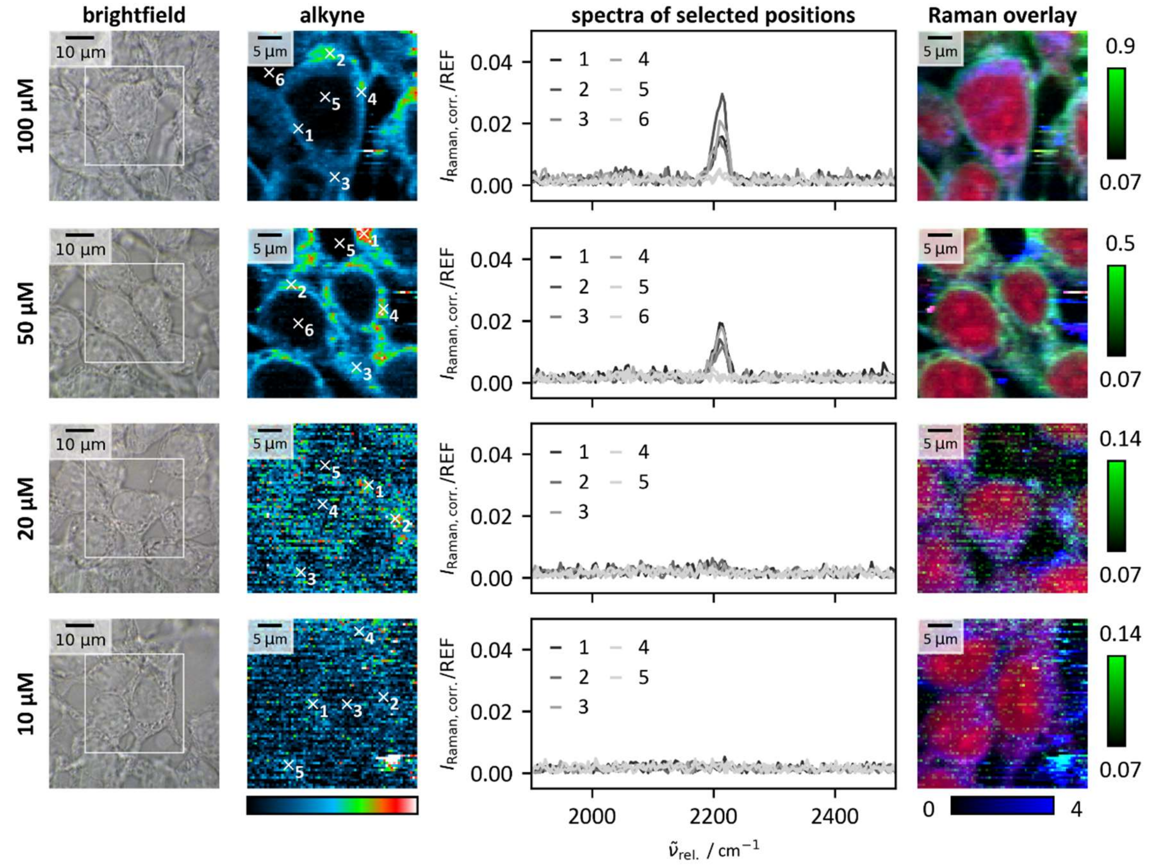

Figure S 8. Raman spectroscopic detection of CHIM 4 inside of HEK 293T cells after treatment with different stock concentrations. The cells were treated after starvation on ice as described in the Experimental Section. Only the CHIM concentration was changed between different dishes of the same batch (100 µM, 50 µM, 20 µM, 10 µM). The figure shows the brightfield camera images with the measured area indicated (left), the distribution of the alkyne (2nd from left, normalization between the 10th and 99.9th quantile in the lower left 55 px × 55 px area), the background corrected single pixel spectra in the silent region (2nd from right, the pixel positions of the selected spectra are indicated in the alkyne image) and the overlay RGB images (right, red: (1342±10) cm^{-1} (nucleus), green: (2212±20) cm^{-1} (CHIM **4**), blue: (2855±20) cm^{-1} (CH_2)). To enable a better comparison, all channels in the RGB image were referenced to the nucleus signal (compare Figure 3). This correction factor was used as well as division factor to bring the selected spectra (2nd from right) to a common scale.

2.7 Cell viability of selected CHIMs

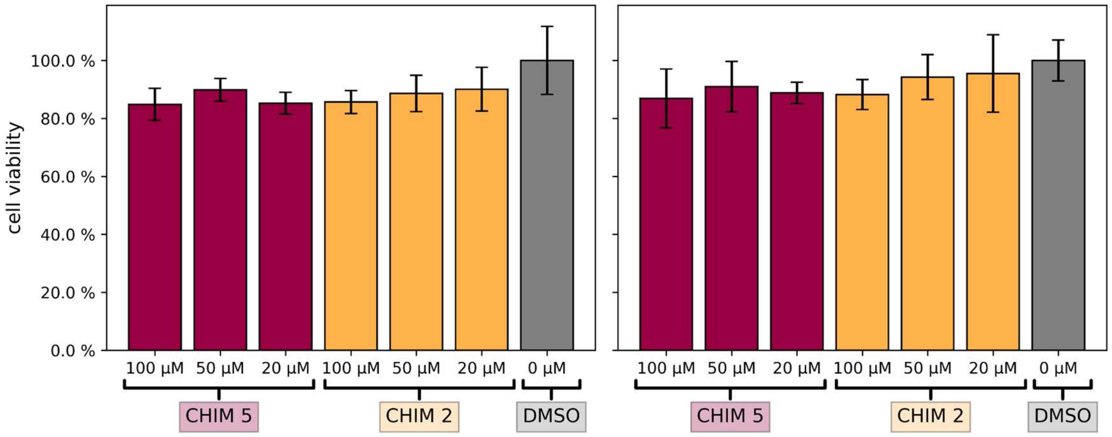

Figure S 9. The toxicity of two selected CHIMs was assessed by an MTT cell viability assay on HEK 293 cells. The derived cell viability values with respect to the DMSO control (DMSO) for two repeats (left and right panel) are shown. Each concentration was analyzed within 6 replicates per repeat. The error bars indicate the standard deviation of the replicates. To specifically assess the toxicity of the CHIMs but not the pre-treatment conditions, the control cells were treated in the same way, only without the addition of the CHIMs, but with all starvation steps and solvent additions. A detailed description of the protocol can be found in the experiments section.

2.8 Selected additional data on incubation experiments with different treatment conditions

2.8.1 Single CHIM incubation experiments with BSA-shuttling

Albumin has also been shown to act as a shuttle for cholesterol, although the mechanism is not fully understood as specific binding sites are lacking.[22] Complexation with BSA in the molar ratios BSA:CHIM 1:2 and BSA:CHIM 1:1 showed no effect on the uptake of the imidazole-version **8** for our short incubation times, whereas it didn't affect cellular uptake of the reference sample **5** (Figure S 10). The procedure described in the Experiments section was used to treat the cells, replacing the composition of the treatment solution (TS) with the following procedure:

TS 2 2.1 µL of a 47.9 mM CHIM stock solution in DMSO was mixed with 0.5 eq. – 1 eq. FAF-BSA in medium (DMEM-F12 + 10 % FCS). The resulting mixture was stocked up with medium to yield a total volume of 1 mL of a solution containing 100 µM of the respective CHIM and 50 µM to 100 µM BSA. The solution was sonicated for 15 min at room temperature.

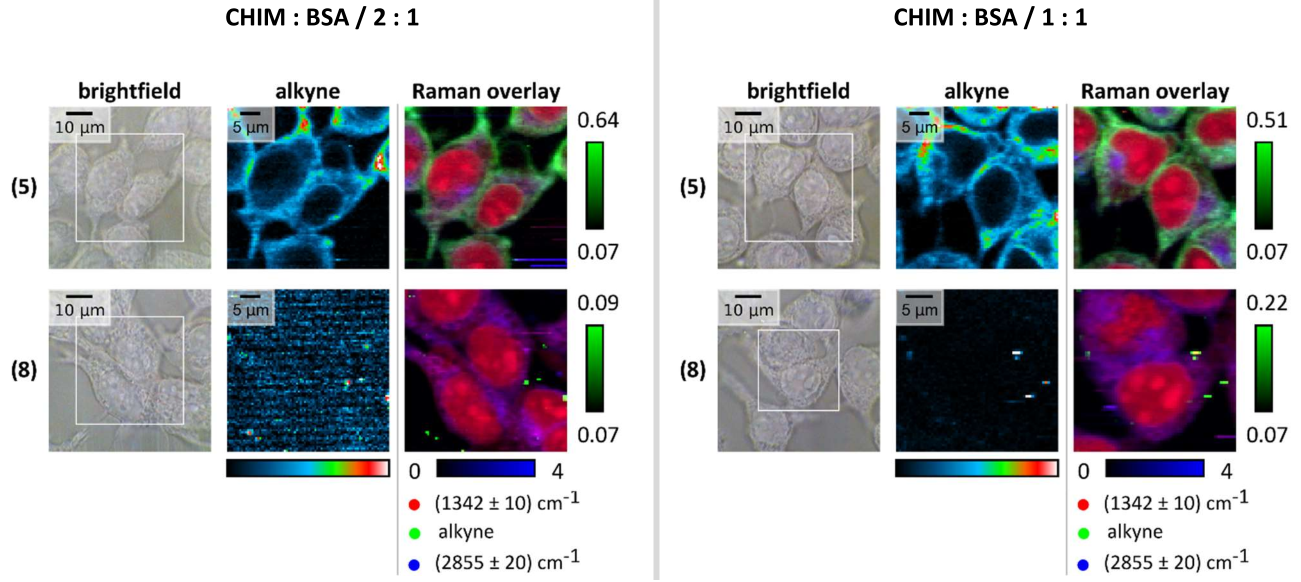

Figure S 10. Visualization of the spatial distribution of the two diyne-tagged CHIMs **5** and **8** in starved HEK 293T cells after a 20 min incubation on ice when using fatty acid-free BSA as a lipid shuttle in molar CHIM:BSA-ratios of 2:1 and 1.1 (CHIM concentration: 100 µM). While the imidazole-based CHIM **8** is still not being taken up, CHIM **5** is distributed across the cell as expected and previously observed without BSA (Figure 3). For RGB normalization see Experimental Section. Alkyne integration ranges: CHIM **5**: (2212±20) cm^{-1}, CHIM **8**: (2245±20) cm^{-1}.

2.8.2 CHIM 8 incubation experiments with MβCD-shuttling

HEK cells were seeded on glass bottom dishes following the procedure in the Experimental Section for the single CHIM incubation experiment and treated, without preceding starvation, for 30 min at 37°C, 5 % CO_2 with a 100 µM CHIM solution containing MβCD (preparation see below). The after-incubation routine is again described in detail in the Experimental section.

TS 3 100 µL MβCD (10 mM in DPBS) was mixed with 10 µL CHIM **8** (10 mM in DMSO) and the resulting solution was homogenized by ultrasonication for 1h at 65 °C to ensure complexation. The MβCD-CHIM was then stocked up with DPBS to 1 mL total volume (final CHIM concentration in the treatment solution: 100 µM) and used instead of the medium during incubation.

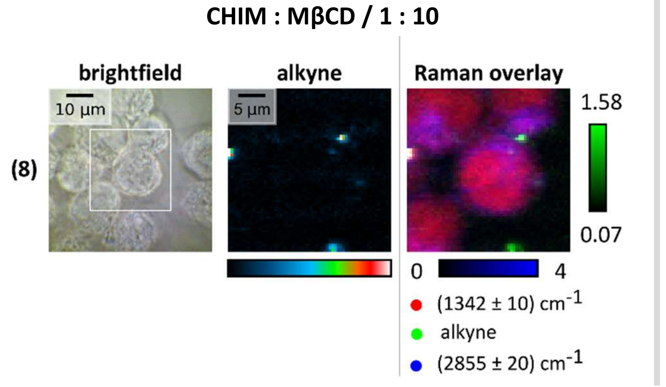

Figure S 11. Exemplary visualization of the spatial distribution of diyne **8** when complexed with MβCD (molar ratio 1 : 10). The complexation did not improve the solubility in an aqueous medium and particles are still visible. During the treatment, significant cell rounding becomes visible. For RGB normalization see Experimental Section. Alkyne integration range: CHIM **8**: (2245±20) cm^{-1}.

2.8.3 Prolonged incubation experiments with continuous CHIM supply

HEK cells were seeded on glass bottom dishes as described in the Experimental Section and treated, without preceding starvation, for 1 h at 37 °C, 5 % CO_2 with a 100 µM CHIM solution (3.1 µL CHIM **6** (47.9 mM in DMSO) + 1.497 mL medium).

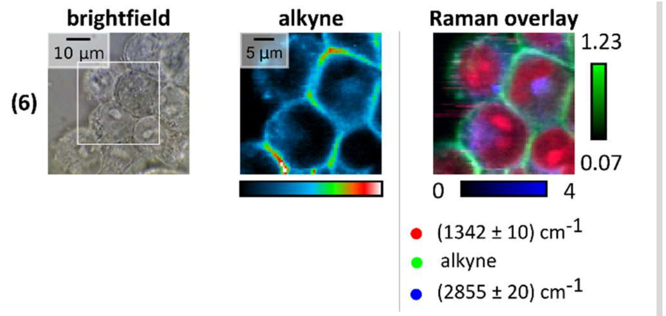

Figure S 12. Visualization of the spatial distribution of diyne-CHIM **6** after incubation with a 100 µM for 1h, 37 °C, 5 % CO_2. Alkyne integration range: CHIM **6**: (2243±20) cm^{-1}.

2.8.4 Prolonged incubation experiments with pulsed CHIM addition

HEK cells were seeded on glass bottom dishes as described in the Experimental Section and treated, without preceding starvation, for 20 min at 37 °C, 5 % CO_2 with a 100 µM CHIM solution. The solution was then replaced by 2 mL of normal culture medium (DMEM + F12 + 10 % BSA) and the cells remained in the incubator for 4 h. Afterward, the cells were washed and fixed as stated in the Experimental section.

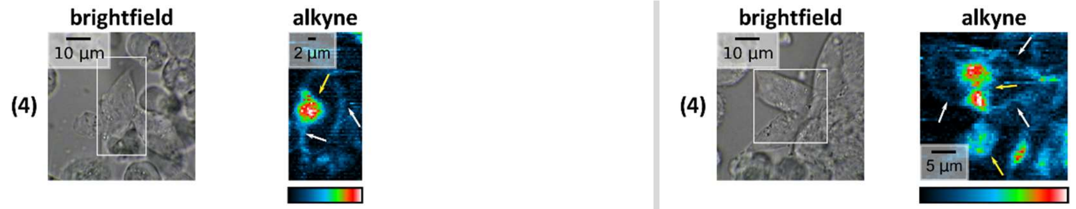

Figure S 13. Visualization of the spatial distribution of diyne-CHIM **4** after incubation with a 100 µM for 20 min in the incubator, subsequent replacement by DMEM + F12 (10 % BSA) and further incubation for 4 h at 37 °C, 5 % CO_2. White arrows: cell membrane localization, yellow arrows: localization in granular structures.

2.9 Additional data concerning the spatial distribution of CHIMs

2.9.1 Distribution of CHIMs along the z-axis:

Complementary to the spatial distribution of the CHIM in the xy-plane, the distribution in the z-direction can also be mapped (Figure S 14). Due to the worse axial resolution compared to the lateral one, the images do not provide detailed information on whether the substance is located only in the membrane or also in parts of the cytoplasm. Since depth profiling requires repeated imaging of the same position, heat- or light-induced destruction of the CHIM must also be considered. Briefly varying the focus after conducting a stack scan often showed a diminished or absent band in the silent region. However, the three images in Figure S 14 show that the triple bond stretching vibration produces a band whose height strongly depends on the depth position, whereas the band-free regions of the silent region just show a noise pattern. Fluctuations in the signal intensity of the upper and lower accumulation spots are most likely due to the aforementioned degeneration or to the increasing glass background.

Supporting the lateral images (Figure S 10), the CHIM can't be found in the nucleus but in adjacent regions including the membranes. The signal of the triple bond thereby colocalizes in the z-direction with the distribution of the CH_2-stretching vibration. Additionally, the depth profiles verify that the CHIM was taken up by the cell. Since HEK cells grow adherently on glass, the appearance of a signal close to the glass supports the idea of incorporation instead of just sticking to the outside.

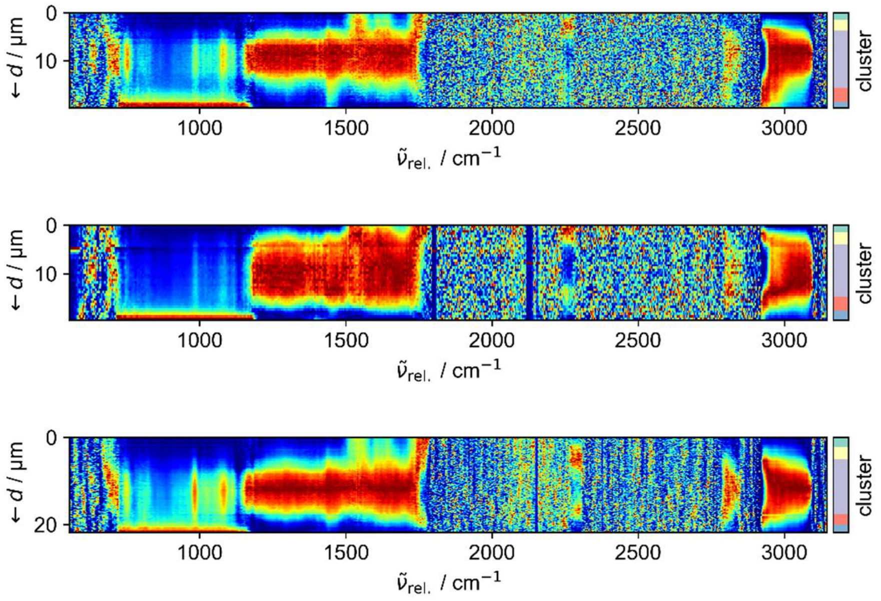

Figure S 14. Depth profile of the CHIM distribution using the examples of a HEK cell treated with CHIM **5** / BSA : 100 µM / 50 µM (top, Δd = 0.25 µm), CHIM **5** / BSA : 100 µM / 100 µM (middle, Δd = 0.5 µm) or CHIM **7** (bottom, Δd = 0.2 µm). The spectral data were obtained by averaging a 2 px × 2 px grid (Δx = Δy = 0.5 µm) in the center of a cell. For each spectral position, the obtained band heights were normalized on a scale with the lowest value being set to 0 and the highest to 1. The band of the diyne-stretching vibration is the only characteristic band in the silent region that shows accumulation at certain depth positions. The averaged spectra were clustered by a k-means cluster analysis with 5 clusters.

2.9.2 The fitting procedure used in Figure 4

The contribution of up to three alkyne-tagged molecules to each pixel spectrum of the hyperspectral grid was extracted by a fitting approach in the wavenumber silent region between 1800 cm^{-1} and 2600 cm^{-1}. To avoid disturbances by the background in the silent window, an arbitrary spectrum taken from a cell-free region in the measured field of view was subtracted first. The remaining baseline shifts were removed by a rubber band correction on a smoothed spectrum in the given region as it behaved more robustly against noise than the previously used SNIP procedure under the same conditions.

Equation (4) consists of a constant term and up to three numeric approximations of Voigt profiles and was used as the model function for fitting in *Python* using the Levenberg-Marquardt algorithm of the *lmfit* module[23].

$$I(x) = c + \sum_{i}^{\leq 3} \frac{A_i \cdot Re[w(z_i)]}{\sigma_i \sqrt{2\pi}} \quad (4.)$$

With:

$$w(z_i) = e^{-z_i^2} \operatorname{erfc}(-iz_i) \quad (5.)$$

$$z_i = \frac{x - \mu_i + i\gamma_i}{\sigma_i \sqrt{2}} \quad (6.)$$

where c is a constant, A the amplitude of the Voigt profile and μ its center position, σ denotes the standard deviation of the Gaussian profile, and 2γ the full width at half maximum of the Lorentzian peak profile.

While the constant term corrects for the offset between the expected zero-line and the determined one that is located at the lower boundary of the smoothed noise, a variable number of Voigt profiles was used for the approximation of the Raman bands of the symmetric alkyne stretching vibration. The fitting thereby proceeded iteratively starting with the number of Voigt profiles equalling the number of triple-bond bearing components in the treatment solution. Overfitting was avoided by constraining the amplitudes to $A_i > 0$ and function widths ($\sigma_i, \gamma_i \in \mathbf{Q}^+$, $\gamma = \sigma$). Additionally, Voigt profiles that parameters (μ, σ, γ) matched the borders of the user-defined fitting interval (μ-6 < μ < μ+6, σ < 20) were removed for the next iteration until a stable state was reached. The result of a fitted spectrum of a membrane pixel is shown in Figure S 15. The spatial distribution was eventually related to the area under the band.

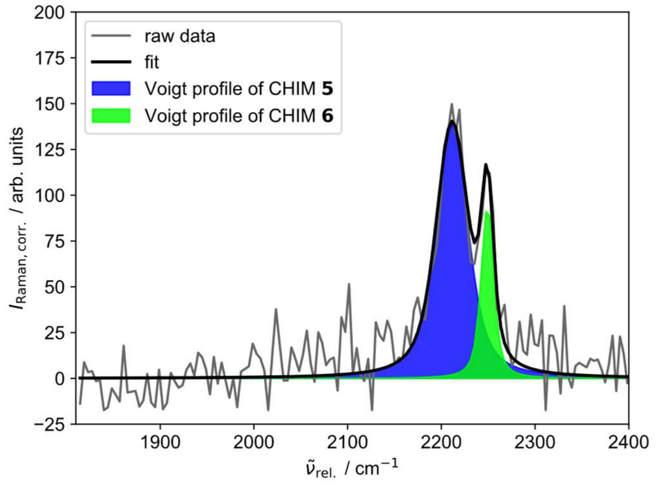

Figure S 15. Visualization of the separation of triple bond components in the silent region by a fitting approach (Equation (4)). The spectrum belongs to a pixel at the outer cell membrane and is associated with a cell treated with two CHIMs and EdU. Thus, the fit model was initialized with three Voigt profiles each corresponding to one component. The number of Voigt profiles required was automatically reduced during the fitting routine, omitting the EdU band since it is not present in the membrane.

2.9.3 Colocalization analysis

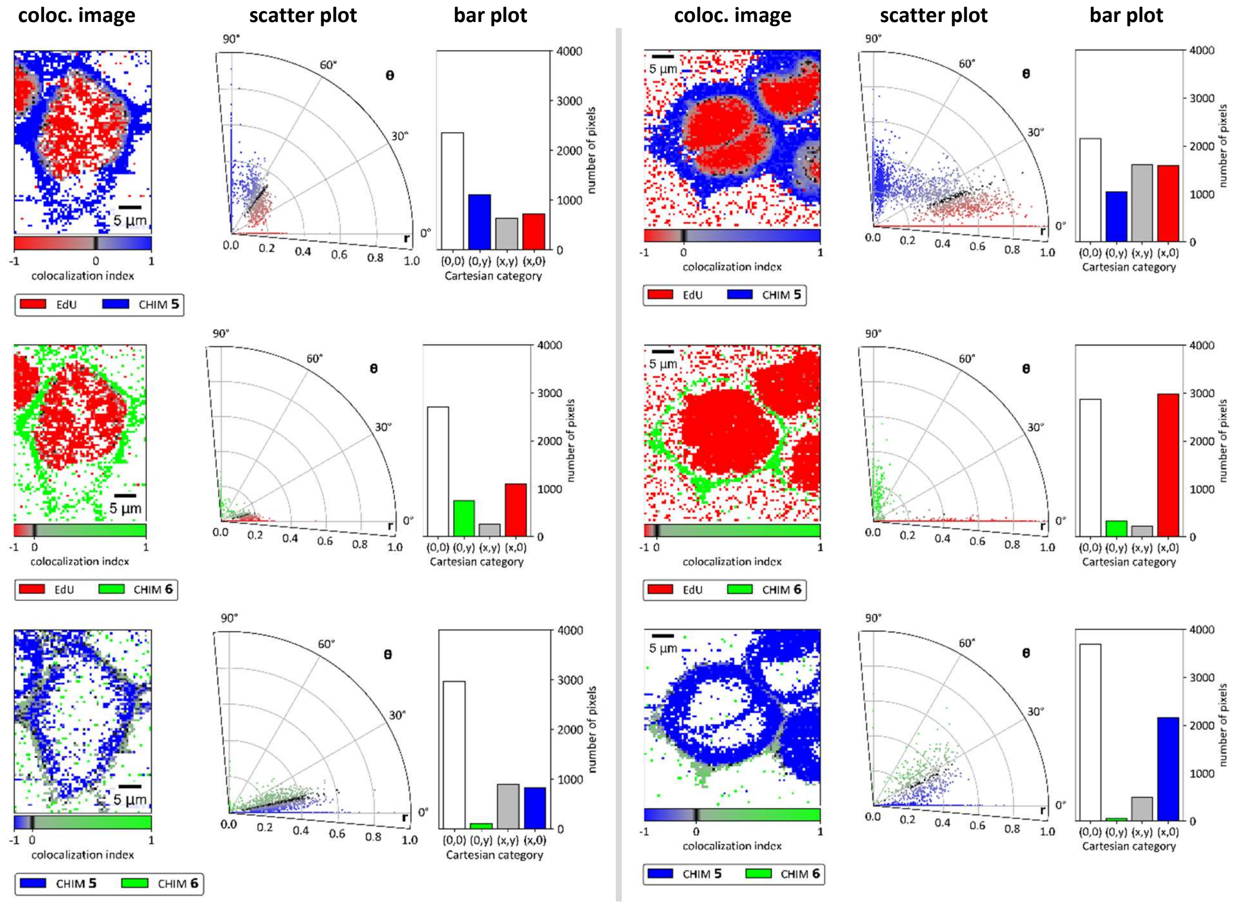

Figure S 16. Results of the colocalization analysis for two different positions in the cell dish (left and right column). Each panel shows a color-coded colocalization map with both analyzed components represented by their colors as used in Figure 4A (left). Black pixels denote the reference point for colocalization obtained by a linear fit. Grayish pixels describe the zone of colocalization, whereas reddish, blueish and greenish shades state a variation of the intensity ratio towards a certain component compared to the fully black reference zone. The polar colocalization scatter plot is depicted in the middle while the bar plot on the right compares the number of pixels that were classified as background (white), only compound **1** (red/blue/green), only compound **2** (red/blue/green) or colocalized (gray).

To determine the areas of colocalization, the pixel intensities of each channel (*x*, *y*) were normalized to the range 0 to 1 and transformed to polar coordinates using Equations (7) and (8).

$$r = \sqrt{x^2 + y^2} \tag{7.}$$

$$\theta = \arctan\left(\frac{y}{x}\right) \tag{8.}$$

The angular coordinate (ϑ) thereby represents the ratio of intensities between the two channels. Pixels located at $\vartheta=0°$ fully belong to component 1 (colocalization index -1, blue), and pixels located at $\vartheta = 90°$ mark positions with only component 2 being present (colocalization index 1, red). A signal from both channels was registered in pixels fulfilling the condition $0° < \vartheta < 90°$. The degree of colocalization was visualized by color coding. The zone possessing a mean colocalization (colocalization index 0, green) was extracted by a fitting approach in Cartesian coordinates using a linear function of type *y = ax*. Pixels that are reddish gray are colocalized but obtain more of component 2 compared to the aforementioned arbitrary zone, while for the bluish gray pixels, the behavior is vice versa.

2.10 Counterstaining experiments with filipin III

2.10.1. Spectral properties of filipin III

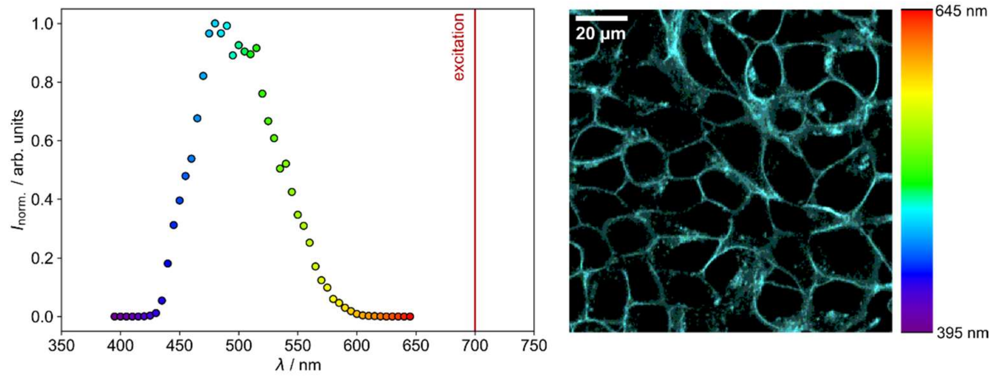

Figure S 17. Spectral properties of filipin III-stained cells. Left: Wavelength resolved emission scan (step width: 10 nm, bandwidth: ±5 nm, center wavelength: 395 nm – 645 nm) of the TPEF signal after excitation with 700 nm. Right: Maximum intensity projection across a stack of images, each colored by center wavelength of the emission window. The spectral position of the maximum of the emission curve is spatially distributed homogeneously.

2.10.2. Localization of filipin III accumulation in 3D-space

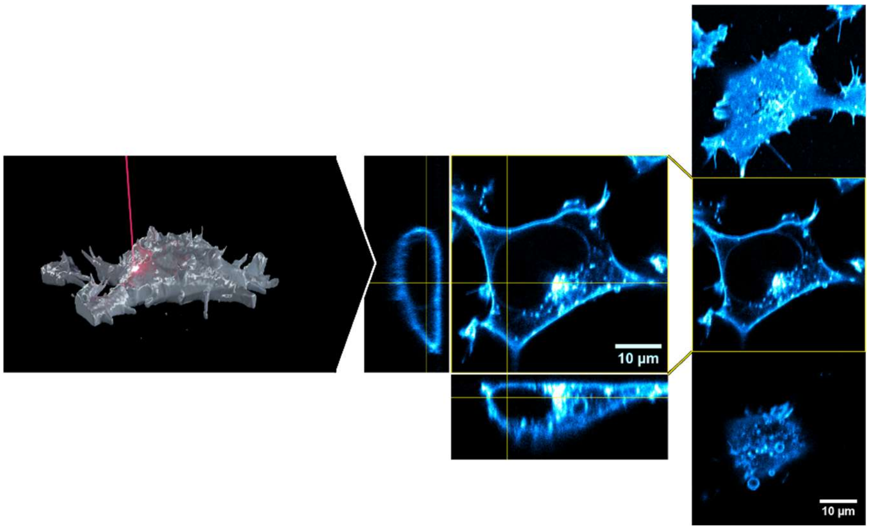

Figure S 18. Filipin staining of untreated HEK cells reveals the native cholesterol distribution to be mainly located in the membranes as well as lipid droplet regions. Pronounced plasma membrane staining is observed in different z-slices (right from top to bottom: slice 54, slice 26, slice 12 of a stack imaged with a z-spacing of 0.2 µm). The orthographic view (middle) confirms full staining of the cell with high accumulation areas in the membranes and lipid droplet regions. Finally, the continuous staining of the membranes allows for a 3D reconstruction of the measured cell (left).

2.10.3. Verification of the cholesterol-like distribution of the CHIMs in short term incubation experiments

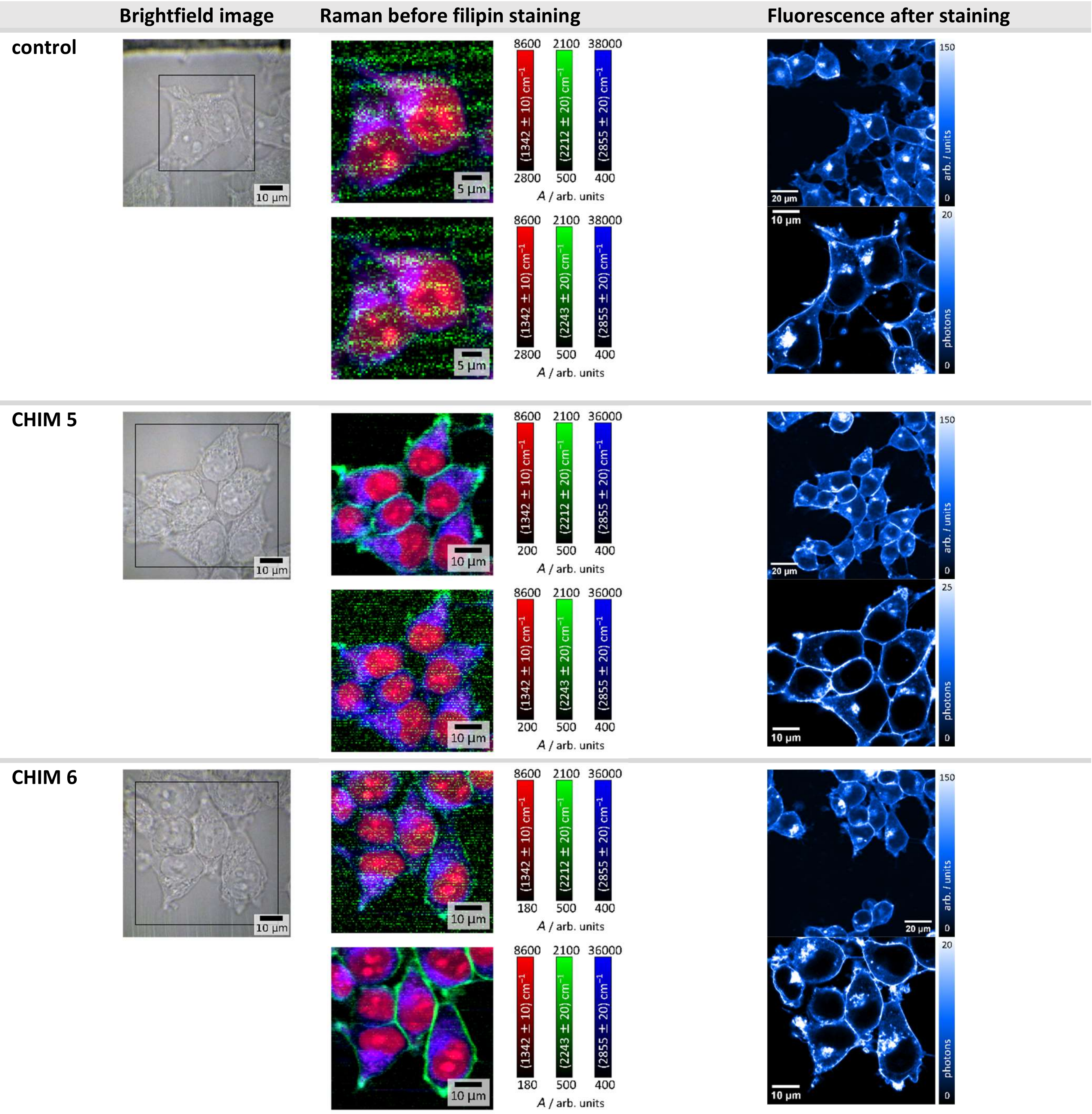

Figure S 19. Comparison of the CHIM distribution after short incubation (20 min on ice, cf. Experimental section) with the cells' total cholesterol contents. The distribution of the CHIMs was analyzed by Raman imaging (integration of the C≡C stretching band in the wavenumber silent region) prior to filipin staining. Shown are the results for the expected regions for CHIM 5 and CHIM 6 for all measured samples, emphasizing the orthogonality of the tag. The total cholesterol content can be visualized by fluorescence imaging after filipin III staining. Overview images (top) were recorded with the Leica Stellaris 8 (400 Hz scan speed, 6 frame averages, 1040 px × 1040 px, 122.85 µm × 122.85 µm, zoom 1.5), detailed views (bottom) with Leica SP8 Falcon (100 Hz scan speed, 16 line averages, 872 px × 872 px, 61.4 µm × 61.4 µm, zoom 3,). For the sake of comparability, the same spot was analyzed with both modalities. Localization of the area of interest was facilitated by a grided petri dish. Due to several washing steps and transport of the dishes between both devices, some cells in the imaged area might get lost. Shown are representative images of 2 biological replicates with three imaged positions within the dish each. Intense staining was particularly observed for the OH bearing CHIM 5.

2.10.4. Raman based colocalization analysis of CHIMs and filipin III

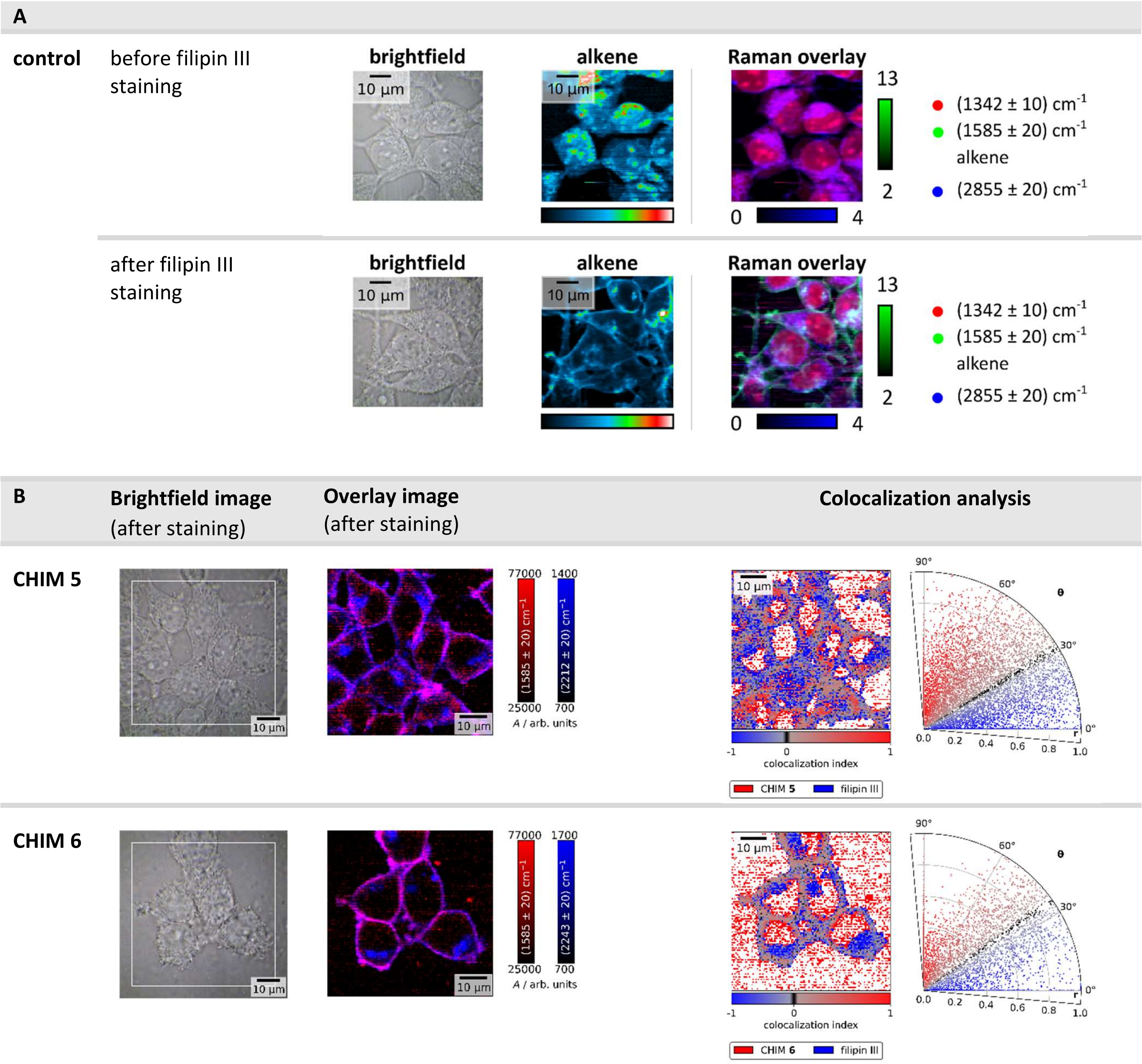

Figure S 20. A: Raman based colocalization analysis of CHIMs and filipin. Filipin III can be located inside the cells by the stretching vibration of the C=C double bonds. Integration of the expected spectral position ((1586±20) cm^{-1}) and scaling to the same range shows a prominent signal in the green channel of the untreated control cells' overlay image that only occurs after filipin III staining and mainly coincides with cellular membranes. Color scaling of the RGB overlay image as with respect to the nuclei as described in the Experimental Section. For better comparability, the upper and lower border for color scaling of the alkene channel in the Raman overlay were set manually. The alkene image in the middle of each panel shows the signal content of the (1586±20) cm^{-1} band in stained and unstained cells within the measured area (the color map scaled individually for each example). **B**: The Raman based localization of filipin III by its inherent molecular vibrations yields staining results that agree with the traditional evaluation of filipin accumulation by fluorescence imaging (Figure S 19). Overlay images of the CHIM distribution of two different CHIMs (red, C≡C) and filipin III (blue, C=C), both evaluated by Raman spectroscopy, show a high degree of colocalization. The colocalization analysis identified the areas of strong colocalization to the membranes as the main accumulation spot after short incubation with cholesterol. For filipin III, staining of lipid reservoirs is also observed, which can be assigned to the place of storage as also discussed in detail elsewhere[24]. Randomly scattered red pixels in the colocalization analysis image can be assigned to noise, possessing much lower intensities in the integration images as the actual signal in the membranes (cf. polar plot).

2.11 Experiments with primary cells

2.11.1. Raman microscopy of primary cells in presence or absence of CHIM 4

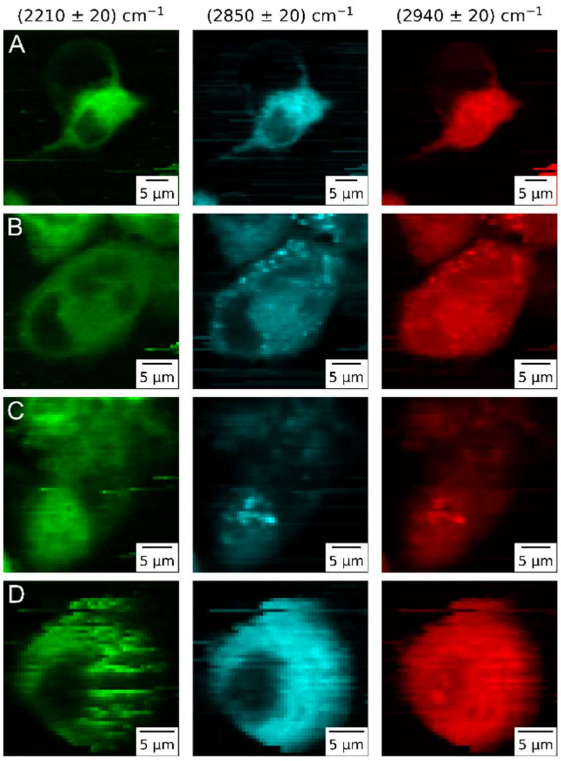

Figure S 21. Raman microspectroscopic integration images of primary macrophages incubated with CHIM **4**. Shown are results from two donors: donor 1 (**A, B**), donor 2 (**C,D**). CHIM 4 can be localized by its band in the wavenumber silent region of the Raman spectrum (2210 cm^{-1}). The bands at 2850 cm^{-1} and 2940 cm^{-1} help to visualize the distribution of lipids and proteins respectively as an indicator for the cell area.

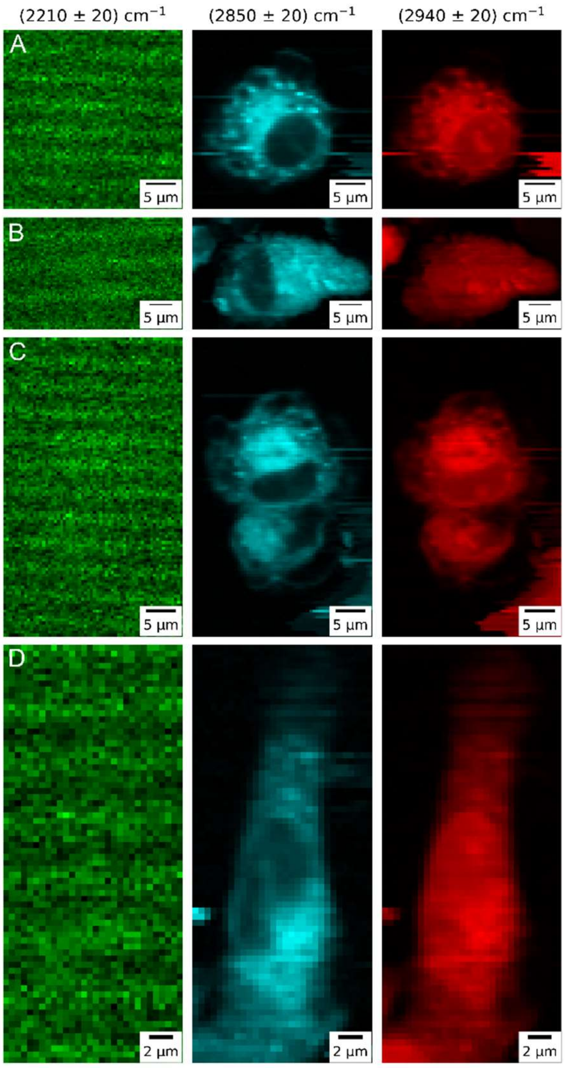

Figure S 22. Raman microspectroscopic integration images of primary macrophages incubated without CHIM **4**. Shown are results from two donors: donor 1 (**A, B**), donor 2 (**C,D**). No natively occurring compounds with Raman bands in the wavenumber silent region of the Raman spectrum are present. The bands at 2850 cm^{-1} and 2940 cm^{-1} help to visualize the distribution of lipids and proteins respectively as an indicator for the cell area.

2.11.2. SRS spectral profile in the wavenumber silent region.

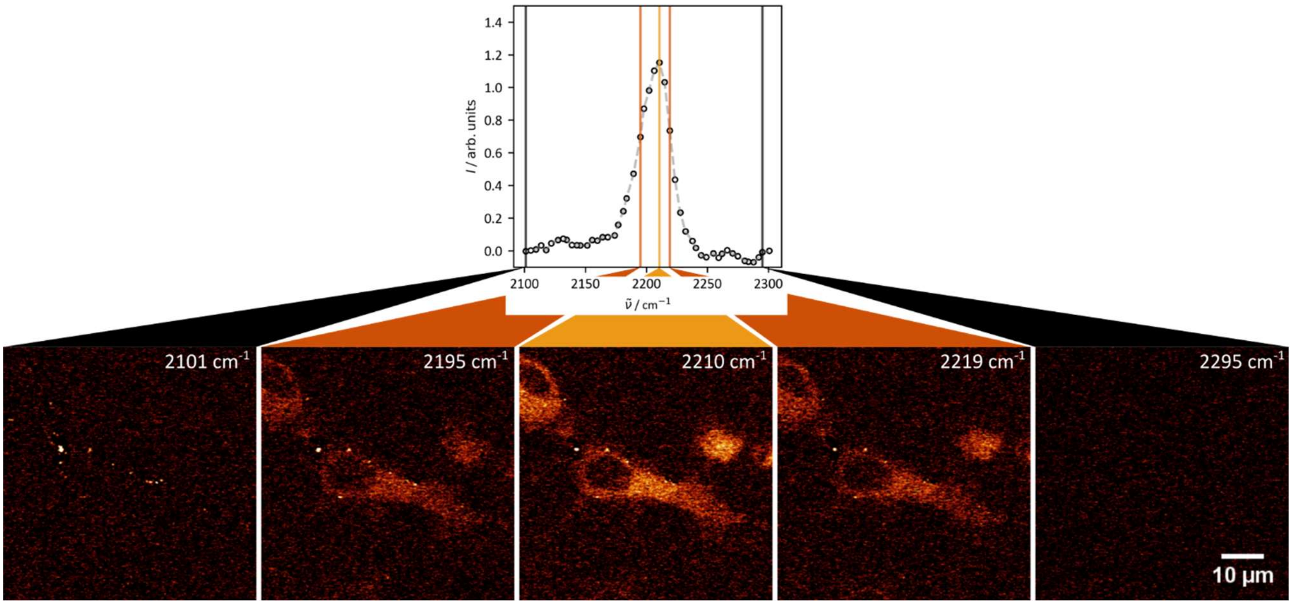

Figure S 23. SRS excitation scan of a primary macrophage. **Top**: SRS spectrum in the wavenumber silent region in the cell region (compare **Figure 5** in the main manuscript). **Bottom**: SRS images after excitation power correction and subtraction of the off-resonance signal at 2300 cm^{-1}. Imaging time: ≈ 8 s per frame (including 4 frame averages).

2.11.3. Localization of CHIM 4 distribution in 3D-space by spontaneous Raman and SRS

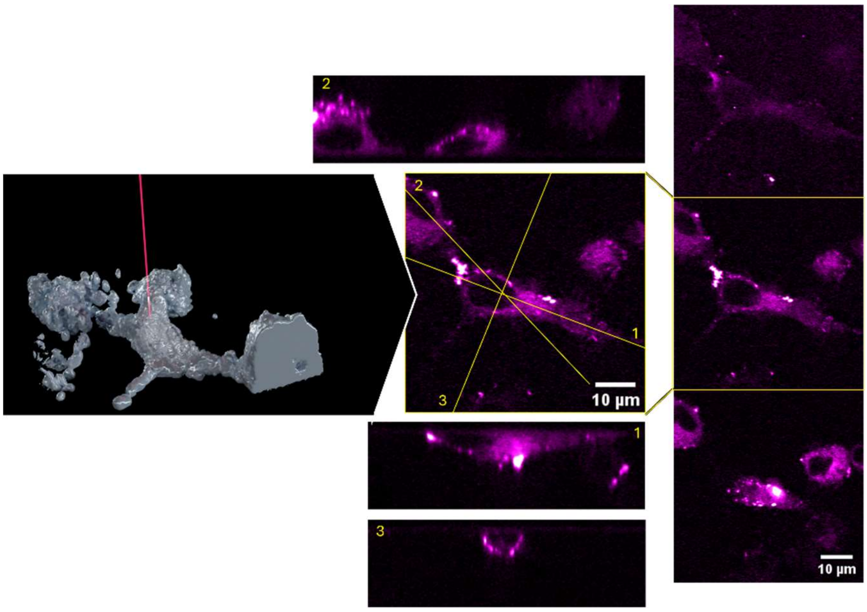

Figure S 24. SRS imaging of CHIM **4**-treated primary macrophages enabled a 3D-reconstruction of the cells based on the presence of the C≡C-stretching mode. By SRS imaging the same visualization tools as for TPEF imaging of fluorescently tagged cells are feasible. For this aspect, we refer to supplementary Figure S18. The presence of CHIM **4** is observed in different z-slices (right from top to bottom: slice 11, slice 19, slice 33 of a stack imaged with a z-spacing of 0.2 µm). The orthographic view (middle) of selected lines confirms full staining of the cell. Finally, the creation of a 3D reconstruction of the measured cell (left) became possible as well.

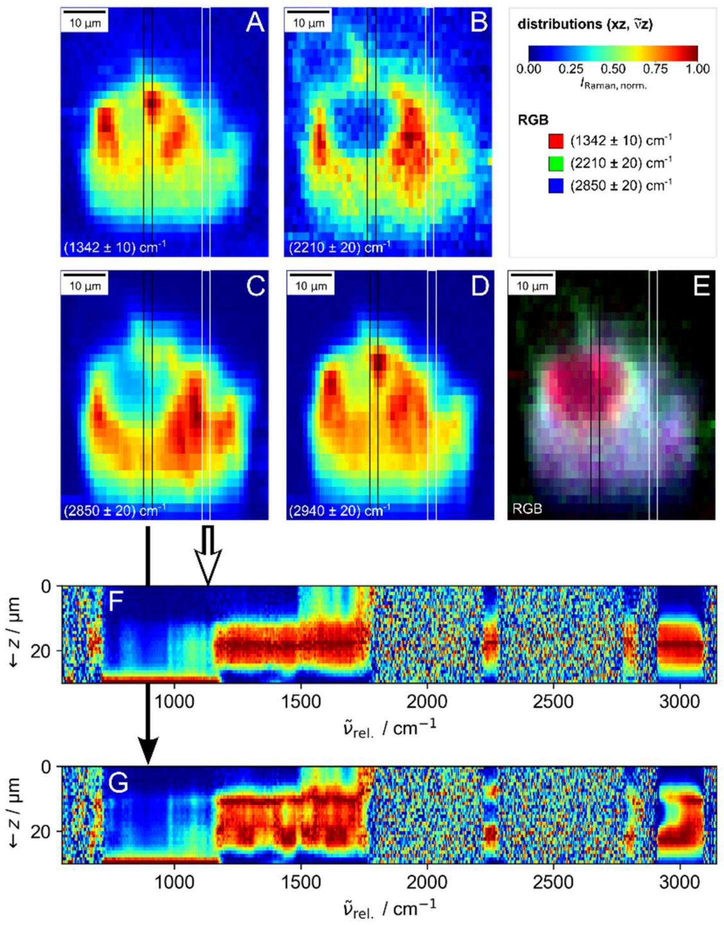

Figure S 25. Creation of z-profile of a CHIM **4**-tagged primary macrophage via Raman microspectroscopy. **A-E**: xz-profiles from a line scan across a cell. Shown are integration maps (color scaling see legend) for the Raman bands at (1342±10) cm^{-1} (**A**, DNA), (2210±20) cm^{-1} (**B**, CHIM **4**), (2850±20) cm^{-1} (**C**, CH_2), and (2940±20) cm^{-1} (**D**, CH_3). **E** is an overlay of **B-D**. **F-G**: Distribution of Raman vibrational frequencies across the depth of the cell at two positions. **F** originates from a position where no nucleus is present and **G** from a position including the nucleus. CHIM **4** is present in the cell, but not the nucleus.

2.11.4. Large area screening with SRS

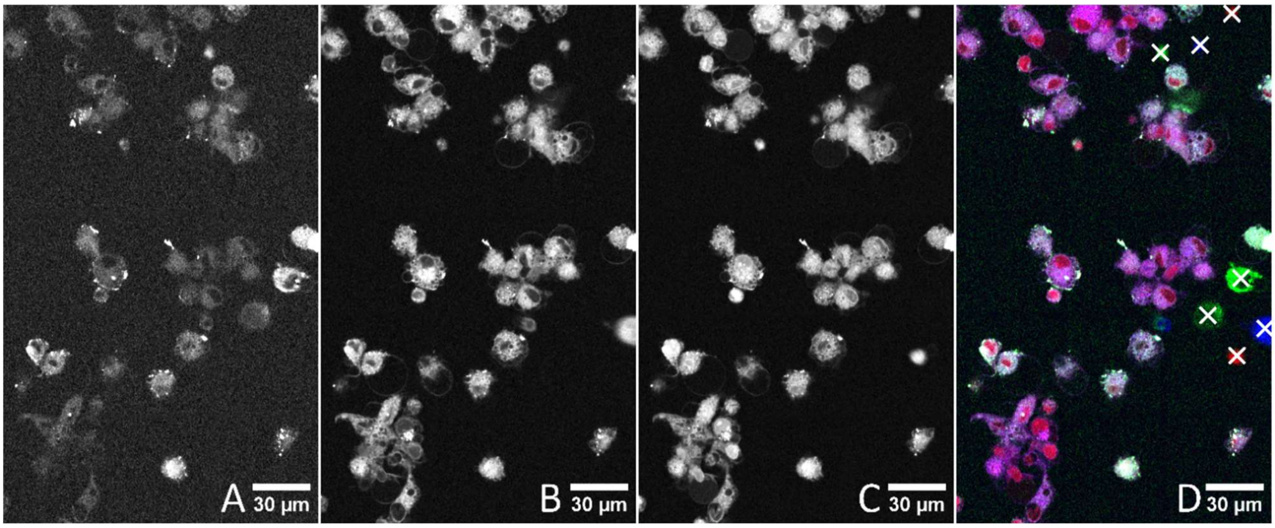

Figure S 26. Large area scan of primary macrophages of donor 1 in a cell dish recorded via SRS imaging. Shown is the SRS signal at 2210 cm^{-1} (**A**, green in **D**), 2855 cm^{-1} (**B**, blue in **D**), 2940 cm^{-1} (**C**, red in **D**) and an RGB overlay of all three (**D**). Crosses refer to positions where the overlay did not match due to movement artefacts of loose cells.

2.12 References

[1] H. Yamakoshi, K. Dodo, A. Palonpon, J. Ando, K. Fujita, S. Kawata, M. Sodeoka, *J. Am. Chem. Soc.* 2012, **134**, 20681.

[2] F. Hu, C. Zeng, R. Long, Y. Miao, L. Wei, Q. Xu, W. Min, *Nat. Methods* 2018, **15**, 194.

[3] H. J. Lee, W. Zhang, D. Zhang, Y. Yang, B. Liu, E. L. Barker, K. K. Buhman, L. V. Slipchenko, M. Dai, J.-X. Cheng, *Sci. Rep.* 2015, **5**, 7930.

[4] E. M. Vartiainen, H. A. Rinia, M. Müller, M. Bonn, *Opt. Express* 2006, **14**, 3622.

[5] R. Houhou, P. Barman, M. Schmitt, T. Meyer, J. Popp, T. Bocklitz, *Opt. Express* 2020, **28**, 21002.

[6] Y. Liu, Y. J. Lee, M. T. Cicerone, *Opt. Lett.* 2009, **34**, 1363.

[7] C. H. Camp Jr., Y. J. Lee, M. T. Cicerone, *J. Raman Spectrosc.* 2016, **47**, 408.

[8] P. H. Eilers, H. F. Boelens, *Leiden-. Univ. Med. Cent. Rep* 2005, **1**.

[9] a) A. Alfonso-García, S. G. Pfisterer, H. Riezman, E. Ikonen, E. O. Potma, *J. Biomed. Opt.* 2016, **21**, 61003; b) S. Hanashima, Y. Ibata, H. Watanabe, T. Yasuda, H. Tsuchikawa, M. Murata, *Org. Biomol. Chem.* 2019, **17**, 8601.

[10] M. Hölttä-Vuori, R.-L. Uronen, J. Repakova, E. Salonen, I. Vattulainen, P. Panula, Z. Li, R. Bittman, E. Ikonen, *Traffic* 2008, **9**, 1839.

[11] a) L. Wei, F. Hu, Y. Shen, Z. Chen, Y. Yu, C.-C. Lin, M. C. Wang, W. Min, *Nat. Methods* 2014, **11**, 410; b) L. Wei, F. Hu, Z. Chen, Y. Shen, L. Zhang, W. Min, *Acc. Chem. Res.* 2016, **49**, 1494.

[12] P. Leppimäki, J. Mattinen, J. P. Slotte, *Eur. J. Biochem.* 2000, **267**, 6385.

[13] N. Kar, D. Gupta, J. Bellare, *Toxicol. Rep.* 2021, **8**, 1054.

[14] a) F. Neese, *WIREs Comput. Mol. Sci.* 2012, *2*, 73; b) F. Neese, *WIREs Comput. Mol. Sci.* 2022, **12**, e1606.

[15] F. Weigend, R. Ahlrichs, *PCCP* 2005, **7**, 3297.

[16] a) A. D. Becke, *J. Chem. Phys.* 1993, **98**, 5648; b) C. Lee, W. Yang, R. G. Parr, *Phys. Rev. B* 1988, **37**, 785.

[17] a) S. Grimme, S. Ehrlich, L. Goerigk, *J. Comput. Chem.* 2011, **32**, 1456; b) S. Grimme, J. Antony, S. Ehrlich, H. Krieg, *J. Chem. Phys.* 2010, **132**, 154104.

[18] F. Neese, F. Wennmohs, A. Hansen, U. Becker, *Chem. Phys.* 2009, **356**, 98.

[19] F. Weigend, *PCCP* 2006, **8**, 1057.

[20] P. L. Polavarapu, *J. Chem. Phys.* 1990, **94**, 8106.

[21] National Institute of Standards and Technology (NIST), "Computational Chemistry Comparison and Benchmark DataBase. Vibrational frequency scaling factors", can be found under https://cccbdb.nist.gov/vsfx.asp, Release 22 (2022). Accessed 02/24/2023.

[22] a) D. M. Charbonneau, H.-A. Tajmir-Riahi, *J. Phys. Chem. B* 2010, **114**, 1148; b) Y. Zhao, Y. L. Marcel, *Biochemistry* 1996, **35**, 7174.

[23] M. Newville, T. Stensitzki, D. B. Allen, A. Ingargiola 2014.

[24] H. J. Lee, W. Zhang, D. Zhang, Y. Yang, B. Liu, E. L. Barker, K. K. Buhman, L. V. Slipchenko, M. Dai and J.-X. Cheng, *Sci. Rep.*, 2015, **5,** 7930; b) M. Hölttä-Vuori, R.-L. Uronen, J. Repakova, E. Salonen, I. Vattulainen, P. Panula, Z. Li, R. Bittman and E. Ikonen, Traffic, 2008, **9**, 1839–1849.
